# A roadmap of brain recovery in a mouse model of concussion: insights from neuroimaging

**DOI:** 10.1186/s40478-020-01098-y

**Published:** 2021-01-06

**Authors:** Xuan Vinh To, Fatima A. Nasrallah

**Affiliations:** The Queensland Brain Institute, The University of Queensland, Building 79, Upland Road, Saint Lucia, Brisbane, QLD 4072 Australia

## Abstract

Concussion or mild traumatic brain injury is the most common form of traumatic brain injury with potentially long-term consequences. Current objective diagnosis and treatment options are limited to clinical assessment, cognitive rest, and symptom management, which raises the real danger of concussed patients being released back into activities where subsequent and cumulative injuries may cause disproportionate damages. This study conducted a cross-sectional multi-modal examination investigation of the temporal changes in behavioural and brain changes in a mouse model of concussion using magnetic resonance imaging. Sham and concussed mice were assessed at day 2, day 7, and day 14 post-sham or injury procedures following a single concussion event for motor deficits, psychological symptoms with open field assessment, T2-weighted structural imaging, diffusion tensor imaging (DTI), neurite orientation density dispersion imaging (NODDI), stimulus-evoked and resting-state functional magnetic resonance imaging (fMRI). Overall, a mismatch in the temporal onsets and durations of the behavioural symptoms and structural/functional changes in the brain was seen. Deficits in behaviour persisted until day 7 post-concussion but recovered at day 14 post-concussion. DTI and NODDI changes were most extensive at day 7 and persisted in some regions at day 14 post-concussion. A persistent increase in connectivity was seen at day 2 and day 14 on rsfMRI. Stimulus-invoked fMRI detected increased cortical activation at day 7 and 14 post-concussion. Our results demonstrate the capabilities of advanced MRI in detecting the effects of a single concussive impact in the brain, and highlight a mismatch in the onset and temporal evolution of behaviour, structure, and function after a concussion. These results have significant translational impact in developing methods for the detection of human concussion and the time course of brain recovery.

**Electronic supplementary material:**

The online version of this article (10.1186/s40478-020-01098-y) contains supplementary material, which is available to authorized users.

## Background

Concussion, or mild traumatic brain injury (mTBI), is a physical trauma-induced pathophysiological process affecting the brain, resulting in rapid onset of typically transient neurological dysfunction, with or without loss of consciousness [1]. Concussions are inherently diverse in nature and of unpredictable outcome. The cognitive sequelae from seemingly minor head injuries incurred during sports can be severe and persistent. Conspicuous cognitive, physical, and emotional disturbances manifest within the first 24 h of the injury, and last for several weeks [2] or longer [3]. More problematic is that certain physiological disturbances can persist beyond the typical 2-week window of clinical recovery, raising concerns about the super-additive risks associated with repeated injury incurred while the brain is still recovering from the effects of the first impact. Indeed, growing evidence suggests that a concussed individual is at high risk for further concussion, and that repeated injuries within a short time window can provoke cumulative brain damage [4].

Objective methods that can accurately diagnose the impact of a concussion on the brain, allowing for better understanding of the underlying pathology, and tracking post-concussion recovery, are thus required. Magnetic resonance imaging (MRI) is a non-invasive imaging method with a number of modalities optimised to detect different aspects of the structural and functional integrity of the brain. The non-invasive nature of MRI also allows repeated imaging measurements, making it ideal for tracking the temporal trajectory of the injury.

Diffusion Tensor Imaging (DTI) is an MRI modality that probes the motion of water molecules in biological tissue [5, 6]. DTI uses a number of metrics to describe water diffusivity in a voxel, namely Fractional Anisotropy (FA) [7] parallel diffusivity (Dp), radial diffusivity (Dr), and mean diffusivity (MD) [8]. DTI has been used to study human concussions and white matter pathologies, in particular, though the results have often been contradictory [9–15]; both reduced FA coupled with increased MD [9–12] and increased FA coupled with decreased MD [13–15] have been reported in the white matter following mTBI. A 2014 meta-analysis of 122 studies suggested that following a concussion, changes in FA are time-dependent—FA increases during the acute phase then decreases in the subacute phases following the impact [16]. Repeated mTBI in rodents [17, 18], single-impact drop-weight model in rats [19] and single-impact piston-driven closed-head injury in mice [20, 21] have shown decreased FA in the white matter. A couple of other studies in rats have shown opposite results: increased FA in the white matter [22, 23]. DTI changes in the grey matter (GM) received less attention though increased FA has been found in human patients with persistent post-concussive symptoms (PPCS) [24] and in vivo animal models of traumatic brain injury (TBI) [25–27].

Neurite orientation dispersion and density imaging (NODDI) is yet another approach that provides more specificity to diffusion imaging changes [28]. NODDI provides measures of neurite (axons and dendrites) density and local structural organisation of neurites by measuring the level of orientation dispersion of restricted anisotropic diffusivities measured with multiple diffusion-weighted directions at higher b-values using orientation dispersion index (ODI) and the contribution fraction of restricted anisotropic diffusion to the total diffusion using neurite density index (NDI) [28, 29]. The few NODDI studies applied to map brain changes in human concussion have reported different findings. Wu et al. detected decreased NDI without any DTI changes in the white matter (WM) of concussion patients aged 35 years old approximately 2 weeks post-injury [30]. Similarly, both decreased NDI (associated with decreased FA, and increased Dp and Dr) [31] and increased NDI (associated with decreased ODI, increased FA and decreased MD) [32] were found in the WM of concussed athletes. Decreased FA and increased ODI were found in the optic tracts of a mouse model of closed-head injury [21].

Studies on the associated integrity of the functional connections in the brain, using resting state functional MRI, following a concussion have also been limited. Resting-state functional Magnetic Resonance Imaging (rsfMRI) detects synchronous low-frequency fluctuations (< 0.3 Hz) of blood oxygen level dependent (BOLD) signals among spatially distinct brain regions in subjects at rest [33]. Regions that have synchronous oscillating fluctuations form brain networks that are functionally connected. The default mode network (DMN) has been the major functional network disrupted after concussion in the absence of structural deficits [34, 35]. Zhou et al. reported reduced functional connectivity in the posterior cingulate cortex and parietal regions and increased functional connectivity around the medial prefrontal cortex in mTBI patients on average 22 days post-concussion [34]. The decreased connectivity in the posterior DMN was positively correlated with cognitive deficits (lower ability to rapidly switching between cognitive sets) while the increased connectivity in the frontal DMN was correlated negatively with posttraumatic depressive symptoms [34]. Similarly, Johnson et al. detected a reduction in the number and strength of the connections in the posterior cingulate cortex and lateral parietal cortices, and an increase in the number and strength of connections in the medial prefrontal cortex in concussed athletes imaged after symptoms had resolved, on average 10 days post-concussion [35]. Other networks found with decreased functional connectivity after concussion include: the Salience Network (SN) [36, 37], in the lateralised cognitive control network [37], and in regions related with motor, sensorimotor, attention, and phonological processing [36]. Connectivity strength between the left dorso-lateral prefrontal cortex and left lateral parietal cortex was reduced with an increasing number of concussions [35]. More recently, Kaushal et al. investigated resting-state functional connectivity in concussed athletes and found that at 48 h post-injury, there were no changes in functional connectivity, despite psychological distress, and oculomotor, balance and memory deficits [38]. At 8 days post-injury, a global increase in functional connectivity was seen with improving symptoms, which recovered by 15 days post-injury [38]. Similarly, Meier et al. reported increased local intrinsic functional connectivity in the right middle and superior frontal gyri at 24–48 h post-injury, which were normalised at 7 days and 6 months post-medical-clearance [39]. To the best of the authors’ knowledge no functional imaging study combining stimulus-evoked functional MRI and rsfMRI were published in a mouse model of closed-head injury.

The conflicting findings in human imaging studies described above suggested the difference might be due to the patients’ background (sex, age, medical history), the injury severity, and the imaging time post-concussion, which affect where each patient was along the injury/recovery time course. Therefore, animal models serve as a way to study concussion by allowing precise control of all the variables or their effects on the injury and manifestation on MRI findings. In this study, we attempted to chart the temporal evolution of the microstructural and functional changes post-concussion in a mouse model with the aim of highlighting the functional-structural mismatch in their recovery trajectories.

## Methods

### Study design

This is a cross-sectional study which included a total of 43 3–4 months old (mice age on impact date: 13.2 ± 1.4 weeks) male mice. Mice were divided into four cohorts: Sham (n = 14, n = 6 day 2, n = 3 day 7, n = 5 day 14), concussion day 2 (CON 2; n = 9), concussion day 7 (CON 7; n = 10), concussion day 14 (CON 14; n = 10). On day 0, all mice in the concussion group were exposed to a concussive impact using our impactor device (see our earlier study [40] for detailed description). The sham animals underwent the exact same procedure but did not receive an impact. After the concussion or sham procedure, the loss-of-righting-reflex (LRR) time was measured for each of the animals. Thirty minutes after recovery, the Neuro Severity Score (NSS) was measured for each of the mice. At day 2, day 7, or day 14, depending on the cohort, the NSS was measured again and all mice underwent Open Field Assessment and MRI scan. Animals were excluded from this study if obvious brain injuries or structural abnormalities were observed on T2-weighted structural MRI images. All experiments were approved by the Institutional Animal Ethics Committee at the University of Queensland (Animal Ethics Committee approval number QBI/260/17).

Data from this study is available, without reservations, on request to the corresponding author.

### Concussion procedure

The concussion model used in this study was used in one of our prior publications [40], where more details can be found; the procedural description is mentioned for clarity and ease for the readers.

The animals were housed in the animal holding facility with a 12-h light–dark cycle, with food and water available ad libitum. Animals were initially anaesthetised with 3% isoflurane in 60% Air 40% O_2_ gas mixture at 2 L/min for 2 min. Mice were then transferred to the impactor device and supported on the body plate. The animal support structure included a reclining body plate to support the mouse’s body and a head plate to support the head. The head plate had a hole to allow a brass piston to deliver the impact from below; two perpendicular lines intersecting the centre of the brass piston were used as crosshairs to aim and position the head. The body plate was reclined to allow the frontal and parietal bones on the mouse’s head to be positioned flat against the head plate. The mouse body was secured in the supine position by a non-slip silicone mattress and two Velcro straps across the chest and abdomen. The tail was secured to the body plate with masking tape to prevent slipping. Anaesthesia was maintained during the securing and positioning for impact at 2% isoflurane in the same gas mixture via a nose cone; total time under anaesthesia was 10–12 min. Time under anaesthesia was kept consistent across different groups and the experimenter aligned all animals, concussed or shams, to the same accuracy standard.

Once the animal was secured on the platform, anaesthesia was discontinued, and the trigger button immediately pressed to induce a concussive impact. Terminal piston velocity used ranged from 5.2 to 5.4 m/s. Mice in sham control group underwent the same procedure without actual impact.

### Behavioural assessment

#### Loss-of-righting-reflex (LRR)

After the impact or sham procedure, the mice were removed from the restraints and laid in the supine position on a warmed surface for recovery. Loss-of-righting-reflex (LRR) time was the time (s) from the moment of impact to the first sign of the animal, righting itself to a prone position.

#### Neuro Severity Score assessment

Thirty minutes after the first sign of righting reflex, all mice were assessed based on a modified Neuro Severity Score (NSS). Another NSS assessment was performed on the day of and prior to the MRI scanning session. NSS is a common assessment 10 tasks scale for neurological deficiency and a detailed description of the tasks can be found in Flier et al. [41]. These 10 tasks included: successful escape from a 30 cm-diameter walled circle with one opening; natural seeking behaviour in an open area; lack of limping/dragging walking gait or grabbing weakness; presence of a straight walk and lack of an abnormal gait; intact startle response to loud noise; 1 cm beam balance; 3, 2, and 1 cm beam walk tasks, and 0.6 cm round stick balance. An additional 6 mm beam walk task was added, raising the number of task and the highest possible score from 10 to 11. The final task described in Flier et al. was the round stick balance [41]; preliminary tests showed several of our mice were sufficiently skilled and motivated to cross the stick similarly to beam walk tasks; thus we decided to add a 6 mm round stick walk into our modified NSS assessment.

#### Open field assessment

Open field activity was assessed on the day of and prior to the MRI scanning session using an elevated circular arena (diameter 77 cm) fenced by 32 cm high boundary. The animals were placed in the centre of the arena and spontaneous activities were tracked and recorded using an overhead camera and Tracker software (Bio-Signal Group, NY, USA) for 10 min. The circular platform was divided into four annuli (with annulus 1 at the centre and annulus 4 at the periphery of the platform) the fraction of total time the animal spent in each annulus was quantified. The Thigmotaxis Index (TI)—an index for anxiety-like behaviours [42], was calculated as TI = (T_ann4_ − T_ann1-3_)/(T_ann4_ + T_ann1-3_) where *T*_ann4_ and T_ann1-3_ represents the time spent in annulus 4 and combined time spent in annuli 1, 2, and 3, respectively.

### MRI experiments

#### Animal handling

Anaesthesia was induced using 3% isoflurane in 60% air, 40% O_2_ at 1L/min. The isoflurane concentration was maintained at 2–2.5% during the preparation which took around 30 min. Each mouse was positioned on an MRI-compatible cradle (Bruker Biospin, Germany) with ear bars and bite bars to reduce head motion. A peritoneal catheter was inserted and fixed to the mouse for delivery of medetomidine (Domitor, Pfizer, USA). For sedation, a bolus of 0.05 mg/kg medetomidine was given intraperitoneally and then sedation was maintained with a continuous infusion of 0.1 mg/kg/h. Once the animal was inside the MRI scanner, isoflurane was then reduced gradually and kept at approximately 0.25% throughout the experiment. The total time under anaesthesia for each animal was approximately 2.5 h. At the end of the scanning session, 1.25 mg/kg atipemazole (medetomidine reversal) (Antisedan, Pfizer, USA) was given intraperitoneally.

##### Structural MRI scans

MRI scans were performed on a 9.4T MRI scanner (Bruker Biospin, Germany) equipped with a cryogenically cooled transmit and receive coil, controlled by a console running Paravision 6.0.1 (Bruker Biospin, Germany). Structural imaging data was acquired using a 2D T2-weighted (T2w) Turbo Rapid Acquisition with Refocused Echoes (TurboRARE) sequence with the following parameters: matrix size = 192 × 192, Field of View (FOV) = 19.2 × 19.2 mm, number of contiguous slices = 52 and slice thickness = 0.3 mm; giving an effective spatial resolution of 0.1 × 0.1 × 0.3 mm, Repetition Time (TR) = 7200 ms, Echo Time (TE) = 39 ms, averages = 4, RARE factor = 8, and bandwidth = 54.3478 kHz.

#### Resting-state fMRI scans

a 2D gradient-echo echo-planar-imaging (GE-EPI) sequence with the following parameters was used: matrix size = 64 × 64, FOV = 19.2 × 19.2 mm, 20 slices of 0.5 mm thickness and 0.1 mm slice gap; giving an effective spatial resolution of 0.3 × 0.3 × 0.6 mm, TR = 1000 ms, TE = 14 ms, averages = 2, flip angle = 70°, bandwidth = 200 kHz, and 600 volumes were acquired with fat suppression, FOV saturation (covering the head tissue inferior to the subject’s brain), and navigator pulses turned on. A block-stimulation design (40 s OFF, 20 s ON) fMRI scan was performed approximately 70 min after the initiation of the medetomidine bolus; stimulation fMRI scan contained six OFF–ON blocks plus a final 40 s OFF block, for a total of 400 s. After the end of the stimulation fMRI scan, a 5 min break was given before rsfMRI scan was started; rsfMRI scan duration was 600 frames for a total of 600 s. A single EPI scan with the same parameters but opposite phase-encoding direction was acquired for distortion correction of the fMRI images.

#### Diffusion MRI scans

Diffusion data were acquired using a diffusion-weighted Imaging (DWI) spin-echo echo planar imaging (SE-EPI) sequence with the following parameters matrix size = 96 × 96, FOV = 19.2 × 19.2 mm, 32 slices of 0.25 mm thickness and 0.05 mm slice gap; giving output spatial resolution of 0.2 × 0.2 × 0.3 mm, TR = 4500 ms, TE = 25 ms, averages = 4, 3 b-value shells with b = 600, 1500, and 2000s/mm^2^, 33 diffusion weighted directions for each shell, and 2 b = 0 images. A pair of reference b = 0 SE-EPI scans were acquired with opposite phase-encoding directions for EPI distortion correction.

### Behavioural data analysis

All statistical analyses of behavioural data were performed in Prism 8 (GraphPad Inc.). Statistically significant threshold was set at *p* value < 0.05 (two-tailed). Analysis of Variance (ANOVA) post hoc tests were corrected for multiple comparisons by controlling False Discovery Rate (FDR).

#### Loss-of-righting-reflex (LRR)

LRR duration was compared between sham animals and all injured animals using Mann–Whitney tests.

#### Neuro Severity Score (NSS)

The NSS score was compared between the sham and all concussed mice at 30 min post-concussion using the Mann–Whitney rank test. The NSS of the concussed mice from the same cohort assessed at 30 min was compared against the NSS on the day of the MRI scan of that cohort using the Wilcoxon matched pairs signed rank sum test. NSS of shams and CON cohorts assessed on imaging day were analysed using Kruskal–Wallis One-Way ANOVA with post hoc tests comparing each CON cohort with sham cohort.

#### Open field assessment

Time spent in each annulus of different cohorts were analysed using repeated measures Two-way ANOVA with Geisser–Greenhouse correction and post hoc tests comparing mean time spent in each annulus of each cohort with every other cohort. TIs were analysed using Brown–Forsythe and Welch One-way ANOVA with post hoc tests comparing mean TI of each cohort with mean TI of every other cohort.

### MRI data processing

All MRI data were exported in DICOM format using Paravision 6.0.1 before converting to NIFTI data format using MRIcron (i.e., the dcm2nii tool) [43]. For the remaining of the processing, the images were given a header file with voxel size 20 times larger than the original size [44].

#### Structural image processing and tensor-based morphometry (TBM)

Structural image signal inhomogeneity correction was applied to the T_2_w structural data using FSL’s FAST tool (https://fsl.fmrib.ox.ac.uk/fsl/fslwiki) [45]. The structural image was then registered rigidly to the Australian Mouse Brain Mapping Consortium (AMBMC, www.imaging.org.au/AMBMC) MRI template resampled to 0.1 mm isotropic resolution using FSL’s FLIRT [45]. The registered images from all animals were averaged to create a study-specific “FLIRT template”. This study-specific template was used as the new template for another iteration of linear registration and “FLIRT template” creation. This process was repeated for three linear registration iterations before each individual structural data was then non-linearly registered to the resulting third iteration study-specific “FLIRT template” using FSL’s FNIRT tool [45] and a study-specific “FNIRT template” was created from the non-linear registered images. Individual structural images were non-linearly registered to the “FNIRT template” and the Jacobian determinant was extracted. Each individual affine-transformation-included Jacobian determinant was calculated from the subject-specific non-linear registration warp; Jacobian determinant for each voxel is a measure that indicate the relative volume change required to warp the template voxel to the individual voxel. Tensor-based morphometry (TBM) [46] was performed using voxel-wise statistics on the Jacobian determinants to assess of local structure absolute volume differences between groups, as opposed to the analysis of local structures normalised by total brain volume.

#### Stimulus-evoked and resting-state fMRI data processing

##### Functional MRI image registration

Opposite phase-encoding direction EPI data were used to calculate the warping field required for EPI distortion correction using FSL’s TOPUP tool [47, 48]. One distortion corrected EPI data was used as reference for an iterative direct EPI-to-EPI non-linear registration/template generation image registration process. All subject’s distortion corrected EPI images were affinely registered to the chosen EPI reference using FSL’s FLIRT [45]; the registered images were then averaged to create the first iteration of a study-specific EPI template. Each subject’s distortion corrected EPI images were then non-linearly registered to the new study-specific EPI template using FSL’s FNIRT tool [45]. The non-linearly registered EPI images were averaged to create the second iteration study-specific EPI template. The non-linear registration process was repeated one more time and the warping field from this iteration used to warp the rsfMRI image to a common space. Recent studies suggested that for the purpose of image registration for functional MRI studies, traditional structural image-based step-wise registration of functional image to same-subject structural image then to common space template can be replaced by direct faster EPI-to-EPI non-linear registration without loss of accuracy [49].

##### Functional data pre-processing

Each functional data set underwent slice-timing correction with FSL’s slicetimer tool, despiked using AFNI’s 3dDespike (https://afni.nimh.nih.gov/) tool, motion corrected using FSL’s MCFLIRT tool (FMRIB’s Software Library) [45] with the forward phase EPI image as the reference image. After motion correction, functional images were distortion corrected using the warping field obtained from EPI pair. Band-pass filter was applied on resting-state fMRI data at 0.01–0.3 Hz using AFNI’s 3dTproject. Pre-processed stimulus-evoked and rsfMRI data were warped to a common space using warp field obtained from the iterative direct EPI-to-EPI non-linear registration/template generation image registration process described above.

##### Functional MRI artefact removal

Artefact removal from registered and pre-processed stimulus-evoked and rsfMRI data were performed with group information guided independent component analysis (GIG-ICA) [50], as implemented in the Group ICA of fMRI Toolbox (GIFT) [51]. The number of independent components for GIG-ICA was set to 50. GIG-ICA has been shown to be superior to not performing artefact removal, or artefact removal using individual-level Independent Component Analysis (ICA) followed by group ICA—a method recommended the Human Connectome Project [52]—for artefacts removal in the context of group ICA [53]. GIG-ICA also avoids the need for potentially subjective, biased, and error-prone human classification of individual level ICA [53] or machine learning training of human-classified data for automated signal/artefact classification. Artefactual components were classified based on criteria similar to prior publications [54, 55]: (1) component spatial map having a large overlap with white matter (WM) and cerebral-spinal fluid (CSF) or ring-like or crescent shape around the edge of the brain or near regions with EPI distortion, (2) frequency power spectrum showing pan frequency distribution, (3) component spatial maps showing alternating positively and negatively correlation bands.

##### Independent component analysis (ICA) of functional MRI data

Artefact cleaned stimulus-evoked fMRI was analysed using temporal-concatenated spatial group ICA, using Infomax algorithm [56, 57], with the number of components set to 18. An independent component with spatial maps (converted to Z-scores and thresholded at Z > 1) that includes the primary somatosensory area (S1) that was shown to be activated in prior fMRI publication [58], and component time course that matched the stimulation paradigm was chosen for further analysis.

Artefact-cleaned rsfMRI data were decomposed into resting-state functional independent components using Independent Vector Analysis (IVA), implemented as combined IVA algorithm (IVA-GL) with multivariate Gaussian (IVA-G) [59] source component vectors plus IVA with Laplace source component vectors (IVA-L) [60]. IVA-GL has been shown to be superior in identifying subject variability and detecting unique biomarkers in fMRI data, compared to more traditional group ICA [61–63], which is potentially more suitable more detecting subtle changes in the post-concussion brains. IVA-GL simultaneously decomposes artefactual-cleaned individual rsfMRI data into 100 Independent Components (ICs) spatial maps and time courses per subject then calculates group-averaged spatial maps and time courses. Group averaged ICs spatial maps were scaled and converted to Z-scores and thresholded at Z > 1 and the ICs were sorted and functionally-relevant components were chosen based on the following criteria: (1) component spatial maps clustered on spatially and functionally feasible grey matter, (2) frequency power spectrum showed power largely in the 0.01–0.1 Hz range (or at least the power in this frequency range was larger than those above 0.3 Hz), (3) component spatial maps had little or no overlap with white matter (WM) and cerebral-spinal fluid (CSF), (4) component spatial maps with bilateral or midline-only areas, or if an IC’s spatial map was unilateral; it was only included if there was another contralateral IC, (5) ICs were rejected as “functionally relevant” if the spatial map had a thickness in the rostral-caudal direction equivalent to one EPI slice in the acquired resolution (single-slice artefacts).

Twenty four ICs were identified and grouped into nine anatomical/network groupings; list of component numbers, component IDs/abbreviations, approximate anatomical structures, and their corresponding anatomical/network groupings are listed in Table 1. More complete spatial maps, time courses, and frequency power spectra for each of the group-level “functionally relevant” ICs are shown in Additional file 1: Data 1.

**Table 1:** Resting-state functional connectivity supra-networks, network, and components: list of component numbers, component IDs/abbreviations, approximate anatomical structures, and their corresponding anatomical/network groupings and functional supra-networks

Supra-network	Network group	Abbreviations	IC no.	Anatomical areas
Default mode–sensory–memory (DMSM)	Anterior Cingulate—Retrosplenial Cortex axis (ACA RSN)	03—ACA-PL	03	Anterior Cingulate Area + Prelimbic area
		06—RSN-ACA	06	Retrosplenial area + Anterior Cingulate Area
		52—RSN	52	Retrosplenial area
		62—ACA	62	Anterior Cingulate Area
		82—RSN	82	Retrosplenial area
		89—IL	89	Infralimbic area
	Hippocampal—Subcortical memory circuit (HP MEM)	05—rHP—RSN—ATN—ACA	05	Subcortical spatial memory pathway (Retrohippocampal Area—Retrosplenial Area—Anterior Thalamic Nuclei—Anterior Cingulate Area)
		08—DG	08	Dentate Gyrus
		34—ATN	34	Anterior Thalamic Nucleus
	Visual—Auditory (VA)	17—AUD	17	Auditory Area
		46—VIS	46	Visual Area
	Primary Somatosensory Area (S1)	04—S1lwlmb	04	Primary Somatosensory Cortex (S1) (Lower Limbs, Trunks) + Posterior Parietal Association Area + Temporal Association Area
		11—S1uplmb	11	Primary Somatosensory Cortex (Upper Limbs)
		31—S1u	31	Primary Somatosensory Cortex (unassigned) + Visual Area
	Thalamus-polymodal association cortex (TH-pmc)	10—TH-pmc-S1	10	Thalamic nucleus (polymodal association cortex) + Primary Somatosensory Cortex
		30—TH-pmc-VA	30	Thalamic nucleus (polymodal association cortex) + Pretectal Region + Visual-Auditory Area
Striatal–motor (STR–MO)	CPu	07—CPu	07	Caudate Putamen
	M1	14—M1	14	Primary Motor Cortex
Salience–supplementary somatosensory (SAL–SS)	Salience Network (SN)	13—S1BF	13	Primary Somatosensory Cortex (Barrel Field, Lower Limbs) + Primary Motor Area
		37—INS-EP	37	Insula + Endopiriform Nucleus
		67—AM	67	Amygdala
	S2	02—S2-a	02	Supplemental Somatosensory cortex (S2)—anterior (bi lateral), Primary Somatosensory Cortex (Nose, Mouth)
		18—S2-p-R	18	Supplemental somatosensory cortex—Right
		45—S2-p-L	45	Supplemental somatosensory cortex—Left

##### Regional homogeneity (ReHo) and intrinsic local functional connectivity analysis

Intrinsic local functional connectivity were calculated for GIG-ICA artefact-cleaned rsfMRI data for all subjects using the regional homogeneity (ReHo) approach [64]. Seven-voxels neighbourhood regional homogeneity (ReHo7) maps of each subject’s rsfMRI data were created by calculating Kendall’s coefficient of concordance (KCC) of each voxel’s time course with its six face-neighbour voxels using AFNI’s 3dReHo tool.

#### Diffusion MRI data processing

##### Diffusion tensor imaging (DTI) data processing

The opposite phase-encoding direction EPI images of the diffusion data were used to calculate the warping field required for EPI distortion correction using FSL’s TOPUP tool [47]. This EPI distortion correction warping field was applied to diffusion data, which was then eddy current distortion corrected and motion corrected using FSL’s *eddy_correct.* Diffusion tensor data was fitted using FSL’s DTIFIT tool using b-value = 1500 s/mm^2^.

One distortion corrected individual-level FA image was used as reference for an iterative direct EPI-to-EPI linear and non-linear registration/template generation image registration process similar to that of rsfMRI EPI data described above (“Stimulus-evoked and resting-state fMRI data processing” section). Warping fields obtain from FA to study-specific FA template FNIRT registration were used to warp other DTI metric images to the common space: MD, Dp, and Dr.

##### Neurite orientation dispersion and density imaging data processing

Multi b-value shell data were fitted using NODDI MATLAB Toolbox (https://www.nitrc.org/projects/noddi_toolbox) [28, 29]; to calculate NODDI metrics. In this in vivo NODDI data fitting, neurites were modelled as impermeable sticks (cylinders with zero radius) in a homogeneous background, neurite orientation distribution was modelled as Watson’s distribution, and the algorithm estimate the hindered diffusivity from the free diffusivity, and neurite packing density using Szafer et al.’s [65] tortuosity model for randomly packed cylinders. NODDI metrics were also warped to the common space using FA to study-specific FA template FNIRT registration warp field. Registered DTI and NODDI metrics images were smoothed to an estimated spatial smoothness of 0.6 mm FWHM using AFNI’s 3dBlurToFWHM.

### Statistical analysis of MRI data

#### Two sample statistical inference of CON cohorts versus sham cohort

Voxel-wise Two-samples *t* tests comparing CON cohorts against sham cohort were performed on registered Jacobian determinants, DTI and NODDI metrics, S1 independent component spatial maps, and ReHo7 KCC maps using permutation inference for the General Linear model [66] as implemented in FSL’s randomise [67]. The number of permutation was set to 10,000, as recommended by a prior study [68]. The resulting statistical map were corrected for multiple comparisons with mass-based FSL’s Threshold-free Cluster enhancement (TFCE) [69] and thresholded at *p* value < 0.05 (two-tailed).

Reproducibility of DTI and NODDI were tested by randomly dividing the sham cohort into two pseudo-groups and two-samples *t* test comparing the pseudo-groups were performed using identical procedure as describe above. The resulting statistical maps are shown in Additional file 3: Fig. S3.

One and two-samples statistical tests were conducted on individual level IC time courses using GIFT’s Mancovan toolbox [70] for IC–IC functional network connectivity (FNC) averages for each cohort and differences between CON and sham cohorts. *T* test for component–component FNC were performed using permutation inference for the General Linear model [66] as implemented in FSL’s randomise package [67], with the number of permutation set to 50,000 or exhaustive, whichever is smaller, and corrected for multiple comparisons with False Discovery Rate at q value < 0.05 for one-sample *t* test and q value < 0.1 for two-sample *t* test.

#### Correlation of behavioural symptoms with local tissue volumes and DTI and NODDI metrics

Registered DTI/NODDI metrics, and ReHo7 KCC maps were correlated with NSS and TI obtained on scanning day, across all subjects. Implementation of voxel-wise statistics were similar to two-sample *t* tests described above.

Three regions of interest (ROIs) were identified to have DTI and NODDI metrics significantly correlated with TI through voxel-wise correlation analysis: the corpus callosum (cc), the polymodal association area of the thalamus (TH-pmc), and the Anterior Cingulate Area (ACA) for further analysis. Another three ROIs were identified to ReHo7 KCC significantly correlated with TI through voxel-wise correlation analysis: the Caudate Putamen (CPu), the Amygdala (AM), and the Insula (INS). Simple linear regression between DTI/NODDI metrics and KCC quantified from the corresponding ROIs and TI were performed on the basis of sham + CON day 2 and CON day 7 + CON day 14, and thresholded at *p* value < 0.05.

T-statistics of two-samples *t* tests of NSS and TI behavioural measures and averaged whole-brain t-statistics maps of two samples *t* tests of Jacobian determinants, DTI/NODDI metrics, and ReHo7’s maps were quantified for each CON cohort comparison with sham cohort and the relative behavioural versus MRI changes were plotted for comparison.

## Results

### Behavioural measures

#### Loss-of-righting-reflex (LRR)

**Fig. 1:**
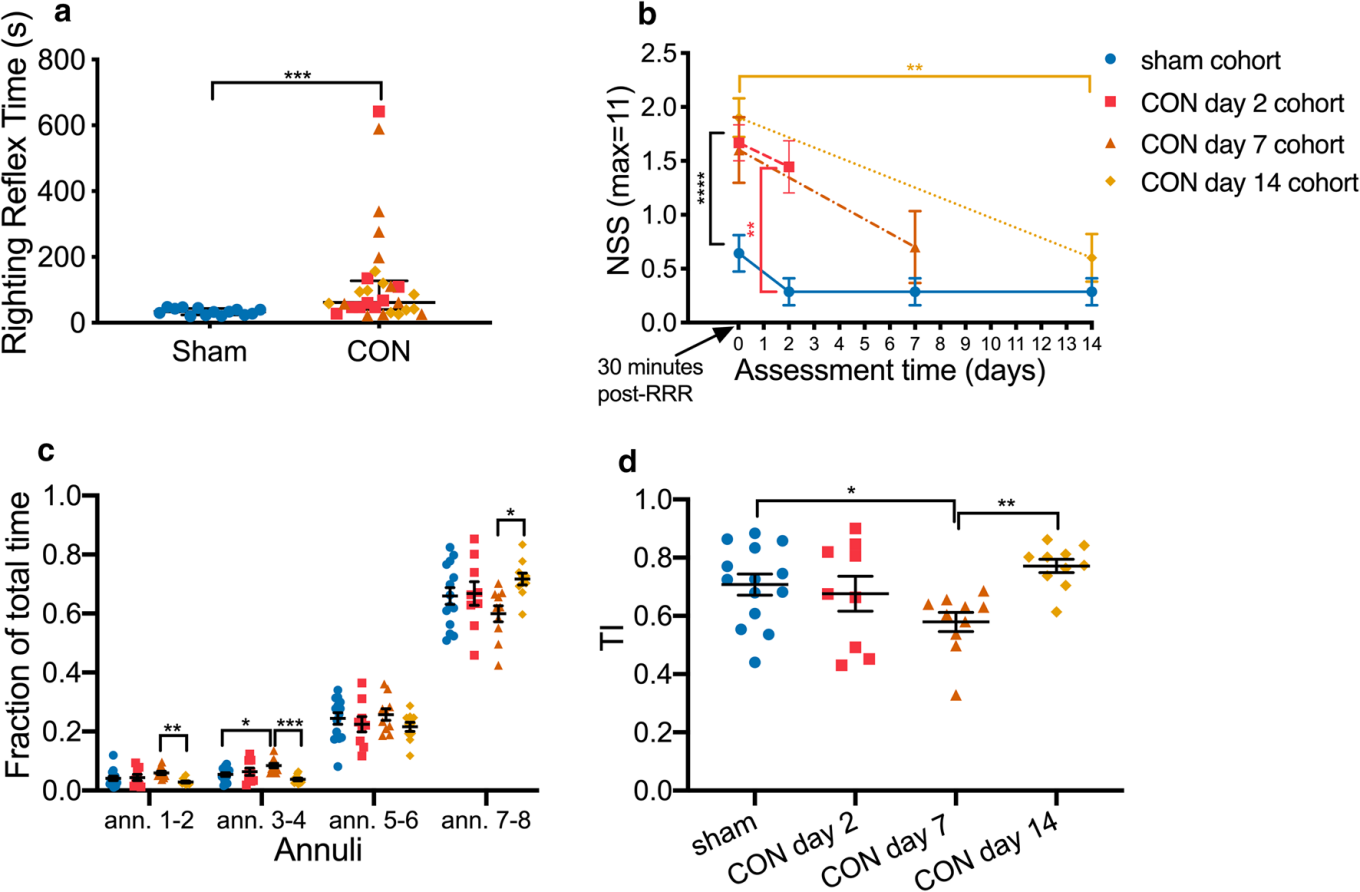
Behavioural assessment of sham and CON cohorts: **a** LRR time of sham (n = 14) and CON (all 3 CON cohorts, n = 29), two-tailed Mann–Whitney test. Lines showing median and interquartile range. **b** NSS of experimental cohorts at 30 min post-concussion and on imaging days: sham (n = 14), CON day 2 (n = 9), CON day 7 (n = 10), and CON day 14 (n = 10). Sham versus all CONs at 30 min: two-tailed Mann–Whitney test. Intra-cohort, 30 min versus imaging day: two-tailed Wilcoxon matched-pairs signed rank test. Inter-cohort NSS on imaging day: Kruskal–Wallis One-Way ANOVA with FDR-corrected (*q* < 0.05, two-tailed) post hoc tests. Data point displayed as mean and standard errors of means. **c** Proportion of time spent in each of four annuli in Open Field Test. Repeated Measures Two-way ANOVA with Geisser–Greenhouse correction and FDR-corrected (*q* < 0.05, two-tailed) post hoc tests. **d** Thigmotaxis Index of each cohort quantified from Open Field Test on imaging day. Brown–Forsythe and Welch One-way ANOVA with FDR-corrected (*q* < 0.05, two-tailed) post hoc tests. *P value < 0.05, **P value < 0.01, ***P value < 0.001

A significant increase in the LRR time was seen in the CON groups 125.6 ± 28.81 (mean ± standard error of mean [s]) compared to the sham group 34 ± 2.65 (*p* value = 0.0005) (Fig. 1a).

#### Neuro Severity Score (NSS) (Fig. 1b)

At 30 min post-concussion, all concussed animals had significantly higher NSS 1.68 ± 0.11 compared to sham animals 0.62 ± 0.13 (*p* value < 0.0001). NSS of CON day 2 on the imaging day was significantly higher than that of the sham cohort (1.44 ± 0.24 vs. 0.29 ± 0.12; FDR’s q value = 0.002). For CON day 7 and CON day 14, there was no significant difference in the NSS score at day 7 and day 14 post-injury compared to that of the sham cohort; CON day 7 (0.7 ± 0.34; FDR’s q value = 0.248) and CON day 14 (0.6 ± 0.22; FDR’s q value = 0.248). There was no significant difference between the NSS measured at day 2 (1.44 ± 0.24) or day 7 (1.44 ± 0.24) compared to the 30 min NSS in the CON day 2 (1.67 ± 0.17, *p* value = 0.35) and CON day 7 cohort (1.6 ± 0.3, *p* value = 0.0625). A significant difference between the NSS measured at day 14 (0.6 ± 0.22) and the 30 min of the CON day 14 cohort (1.9 ± 0.18, *p* value = 0.0039) was seen.

#### Open field test

Two-way ANOVA showed significant annuli (*p* value < 0.0001) and annuli × cohort effect (*p* value = 0.03) but an insignificant cohort effect (*p* value = 0.2) on the fraction of time spent in each annulus. CON day 7 cohort animals spent significantly more time in the annuli towards the centre compared to CON day 14 animals and significantly more time in annulus 2 than sham animals (Fig. 1c). There was significant difference of TIs among different cohorts (*p* value = 0.022) and CON day 7 cohort to have significantly lower TI compared to sham (FDR’s q value = 0.03) and CON day 14 cohorts (FDR’s q value = 0.0009) (Fig. 1d).

### MRI findings

#### Diffusion MRI findings

**Fig. 2:**
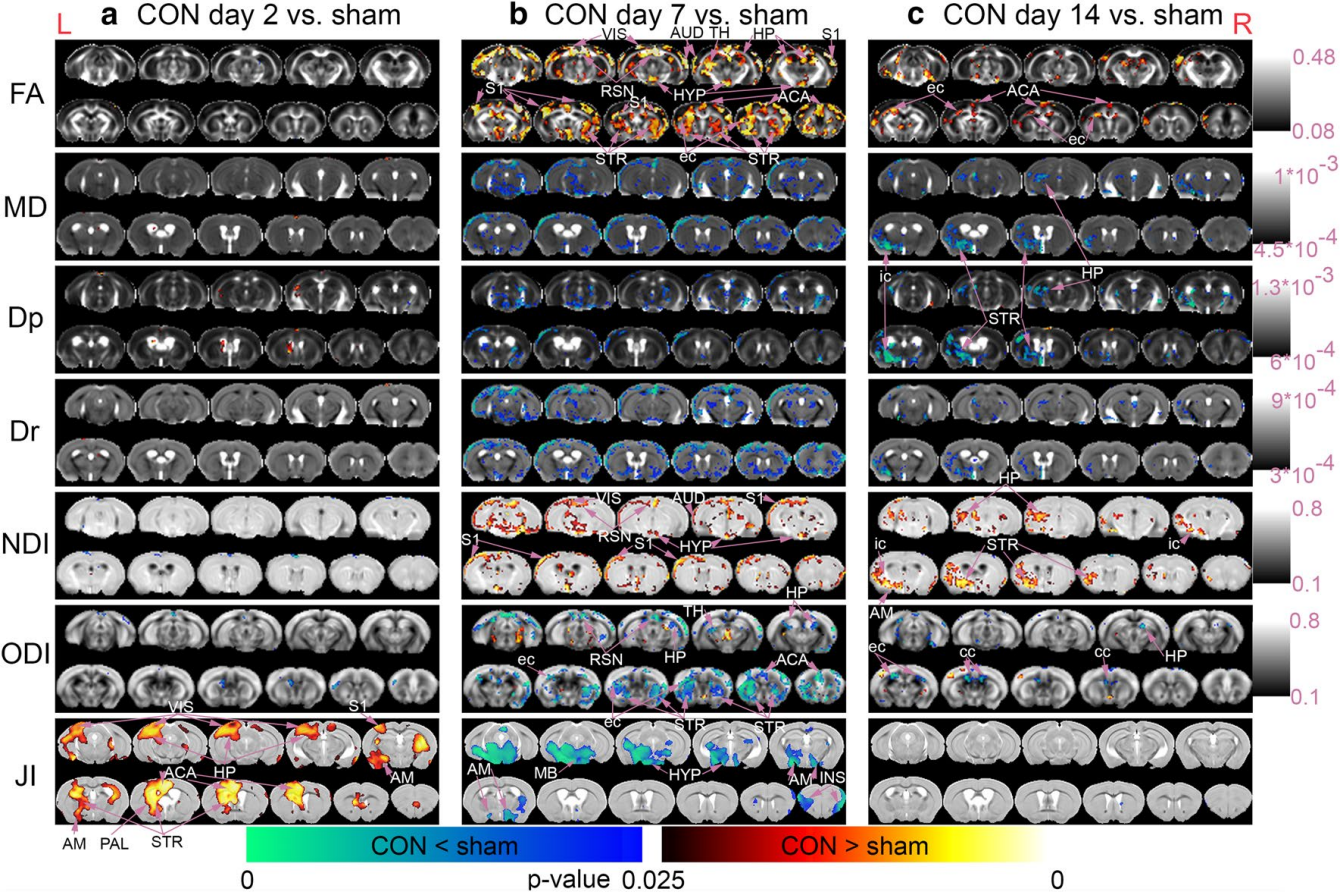
DTI and NODDI metric changes from day 2 to day 14 post-concussion. Voxel-by-voxel statistical analysis results of Diffusion Tensor Imaging (FA = Fractional Anisotropy, MD = Mean Diffusivity, Dp = Parallel Diffusivity, Dr = Radial Diffusivity) and Neurite Orientation Dispersion and Density Imaging metrics (NDI = Neurite Density Index, ODI = Orientation Dispersion Index) and Tensor-based Morphometry with Jacobian Index (JI) of **a** CON day 2 (n = 9) versus sham (n = 14), **b** CON day 7 (n = 10) versus sham (n = 10), and **c** CON day 14 (n = 10) versus sham (n = 14). Statistical map thresholded at *P* value < 0.05 (two-tailed), unpaired two sample *t* test, implemented as permutation tested for the General Linear Model, corrected for multiple comparisons with mass-based FSL’s Threshold-free Cluster enhancement (TFCE). Statistical maps were overlaid on the averaged and registered DTI and NODDI metrics maps corresponding to the statistical maps (DTI and NODDI results) and structural template (TBM results). Corresponding grey scale map for each averaged DTI and NODDI metrics maps were provided; units for Dp, Dr, and MD were in mm/s^2^. ACA = Anterior Cingulate Area, AM = Amygdala, AUD = Auditory Area, cc = corpus callosum, ec = external capsule, HP = Hippocampus, HYP = Hypothalamus, ic = internal capsule, INS = Insula, MB = Midbrain, PAL = Palladium, S1 = Primary Somatosensory Cortex, RSN = Retrosplenial Area, STR = Striatum, TH = Thalamus, VIS = Visual Area. Red anatomical orientation marker L = Left, R = Right. An enlarged view of the DTI and NODDI metrics and the identified regions of interest (ROIs) can be found in Additional file 3: Fig. S3

No significant changes of brain tissues’ DTI and NODDI metrics were detected at day 2 post-concussion compared to shams (Fig. 2a). At day 7 post-concussion, increased FA was detected at day 7 post-concussion compared to shams in both grey matter—the visual, auditory, retrosplenial, hippocampal, and Primary Somatosensory Area (S1), the thalamus, the hypothalamus, the striatum, and the anterior cingulate cortex—and white matter—the external capsule (Fig. 2b, FA). This increased FA in the grey matter was driven mostly by reduced MD and Dr, in the visual, auditory, retrosplenial and S1, the thalamus, the hypothalamus, and the anterior cingulate cortex (Fig. 2b, MD and Dr). Decreased Dp was detected in the internal capsule (Fig. 2b, Dp). Increased NDI was detected in the grey matter—the visual, auditory, retrosplenial, and S1 areas, and the hypothalamus (Fig. 2b, NDI). Decreased ODI was detected in both grey matter—the visual, auditory, retrosplenial, hippocampal, and S1 areas, the striatum, and the anterior cingulate cortex—and white matter—the external capsule (Fig. 2b, ODI).

At day 14 post-concussion, DTI and NODDI changes were still although to a lesser extent compared to day 7. Increased FA was detected in both grey matter—the auditory area and anterior cingulate cortex—and white matter—the external capsule (Fig. 2c, FA). Decreased MD, driven by both Dp and Dr decreases was found in the S1, striatum, and internal capsule (Fig. 2c, MD, Dp, and Dr). Increased NDI was detected over the same regions that had decreased MD, Dp, and Dr (Fig. 2c, NDI). Decreased ODI were detected in small areas in the hippocampus, external capsule and corpus callosum (Fig. 2c, ODI).

#### Structural MRI findings

TBM detected increased ventricle volumes and local tissue volumes in the auditory, visual, and primary somatosensory areas, the hippocampus, anterior cingulate cortex, and the amygdala at day 2 post-concussion compared to shams (Fig. 2a, JI). At day 7, a decrease in tissue volume was seen in the midbrain, the hypothalamus, the amygdala, and the insula (Fig. 2b, JI). No changes were seen at day 14 (Fig. 2c, JI).

#### Functional MRI findings

##### Stimulus-evoked fMRI changes post-concussion

**Fig. 3:**
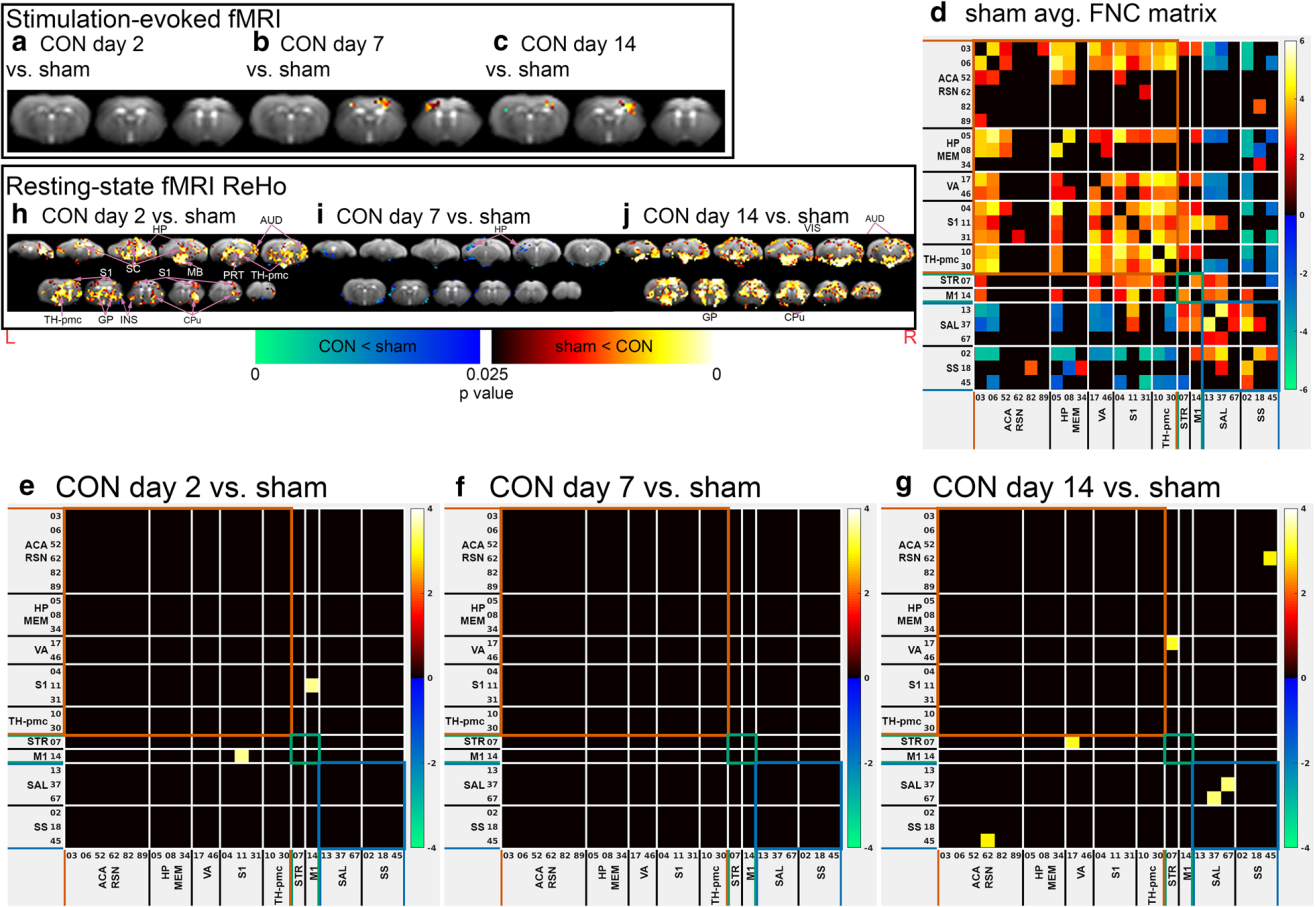
Functional MRI of concussed and sham animals. **a**–**c** Post-concussion stimulus-evoked fMRI activity changes of **a** CON day 2 versus sham, **b** CON day 7 versus sham, **c** CON day 14 versus sham. **h**–**j** Post-concussion changes in local intrinsic functional connectivity in (H) CON day 2 versus sham, (I) CON day 7 versus sham, and **j** CON day 14 versus sham. **a**–**c**, **h**–**j** Statistical map thresholded at *p* value < 0.05 (two-tailed), unpaired two sample *t* test, implemented as permutation tested for the General Linear Model, corrected for multiple comparisons with mass-based FSL’s Threshold-free Cluster enhancement (TFCE). Statistical maps were overlaid on the study-specific averaged EPI images. Red anatomical orientation marker L = Left, R = Right. ACA = Anterior Cingulate Area, AM = Amygdala, AUD = Auditory Area, CPu = Caudate Putamen, GP = Globus Pallidus, HP = Hippocampus, INS = Insula, M2 = Secondary Motor Cortex, MB = Midbrain, PRT = Pretectal area, S1 = Primary Somatosensory Cortex, RSN = Retrosplenial Area, TH-pmc = Thalamus-polymodal association cortex, VIS = Visual Area. **d** Average functional network connectivity (FNC) matrices among Independent Components (ICs) identified by IVA-GL of (**a**) sham (n = 14). Colour scaled by z test statistics; non-black cells were defined as component–component connectivity deemed statistically significant. One sample *t* tests, permutation-tested, and FDR-corrected (q value < 0.05, two-tailed). **e**–**g** Post-concussion FNC changes of **e** CON day 2 versus sham, **f** CON day 7 versus sham, **g** CON day 14 versus sham. Colour scaled by *z* test statistics; non-black cells were defined as component–component connectivity deemed statistically significant. Two sample *t* tests, permutation-tested, and FDR-corrected (q value < 0.1, two-tailed)

Group ICA of stimulus-evoked fMRI showed no difference in spatial extents of the S1 IC spatial maps associated with functional activation during forepaw stimulation at day 2 post-concussion (Fig. 3a). An increased spatial extent of the S1 IC spatial maps was detected at day 7 and 14 post-concussion (Fig. 3b, c), indicating increased neural responses to somatosensory stimulation.

##### Resting state functional network connectivity architecture

Three main supra-networks based on the connectivity architecture: default mode–sensory–memory (DMSM), the salience–supplementary sensory (SAL–S2), and the striatal–motor (STR–MO) supra-network (Fig. 3d). Supra-network refers to a collection of networks in this study determined to have some common characteristics in terms of connectivity to other networks within the same supra-network while different connectivity characteristics in regard to networks in other supra-networks.

The DMSM supra-network consisted of Default Mode Network-like ICs along the Anterior Cingulate–Retrosplenial Cortex axis (ACA RSN group: IC 03, 06, 52, 62, 82, 89), Hippocampal–Subcortical memory circuit (HP MEM group: IC 05, 08, and 34), Primary Somatosensory and Association Area (S1 group: IC 04, 11, and 31), Visual–Auditory areas (VA group: IC 17 and IC 46), and Thalamic polymodal association cortex (TH-pmc group: IC 10 and 30). The strong functional connectivity among CG RSN and VA areas were consistent with the earlier characterisation of the Default Mode Network (DMN) in the rodents [71, 72] and the overall strong connectivity within the DMSM supra-network was consistent with earlier whole brain group ICA functional network characterisation of the mouse brain [55]. IC 05 spatial maps showed several regions strongly connected to one another: the retrosplenial area (RSN), the retrohippocampal (rHP) area, the Anterior Thalamic Nuclei (ATN); this IC was also positively correlated with the anterior cingulate and retrosplenial ICs (IC 03, 06, and 52), and the retrohippocampal IC (IC 08). These connections resemble the cortical memory pathway of HP > RSN > ACA and a subcortical memory pathway of HP > ATN > RSN/ACA, that support head direction, spatial navigation/memory/coding, and emotion [73–75]. Resting-state functional connectivity was known to have a basis in structural connectivity of direct nerve projections [76].

The SAL–SS supra-network consisted of the Primary Somatosensory Area, Barrel Field (S1BF, IC 13), the Supplementary Somatosensory Areas (S2 group: IC 02, 18, and 45), and the Salience Network (SN group: IC 37 [Insula area] and IC 67 [Amygdala]). The SAL–SS supra-network is characterised by positively correlated functional connectivity of ICs within the supra-network and negatively correlated functional connectivity of its ICs with ICs of the DMSM supra-cluster.

The STR–MO supra-network consisted of the Caudate Putamen (CPu, IC 07) and the Primary Motor Area (M1, IC 14). The distinguishing feature of this supra-network is its components have a positive correlation within the supra-network and with the other two supra-networks.

One-sample *t* test results matrices of IC–IC FNC of CON cohorts are shown in Additional file 2: Fig. S2A-C.

##### Whole brain resting-state functional connectivity changes post-concussion

Figure 3e–g reflect the two-sample *t* test comparison of the CON cohorts compared to the sham cohort. At day 2 post-concussion, concussed mice had increased functional connectivity between the M1 and S1, upper limbs (Fig. 3e). At day 7 post-concussion, no IC–IC FNC connectivity change was detected (Fig. 3f). At day 14 post-concussion (Fig. 3g), the increased connectivity was detected in the CPu—Auditory Area (AUD), Left S2—ACA, and Amygdala (AM)—Insula (INS) connections.

##### Local intrinsic functional connectivity changes post-concussion

At day 2 post-concussion, concussed mice had increased local functional connectivity in several regions in the DMSM supra-network (S1, TH-pmc, and HP), as well as the Salience-like network (INS and AM), and the CPu (Fig. 3h). The increased local connectivity at day 2 subsided at day 7 post-concussion, with only decreased local connectivity in a small area of the hippocampus (Fig. 3i). However, at day 14 post-concussion, increased local functional connectivity were detected in many regions of the brain, including the regions in the DMSM supra-network–DMN (ACA, RSN), VA (VIS, AUD), and S1 networks, the SAL–SS supra-network–SN (INS and AM), and the CPu (Fig. 3j).

Averaged ReHo7 maps of sham and CON cohorts are shown in Additional file 2: Fig. S2D-G.

### Correlation of MRI findings with behavioural symptoms

**Fig. 4:**
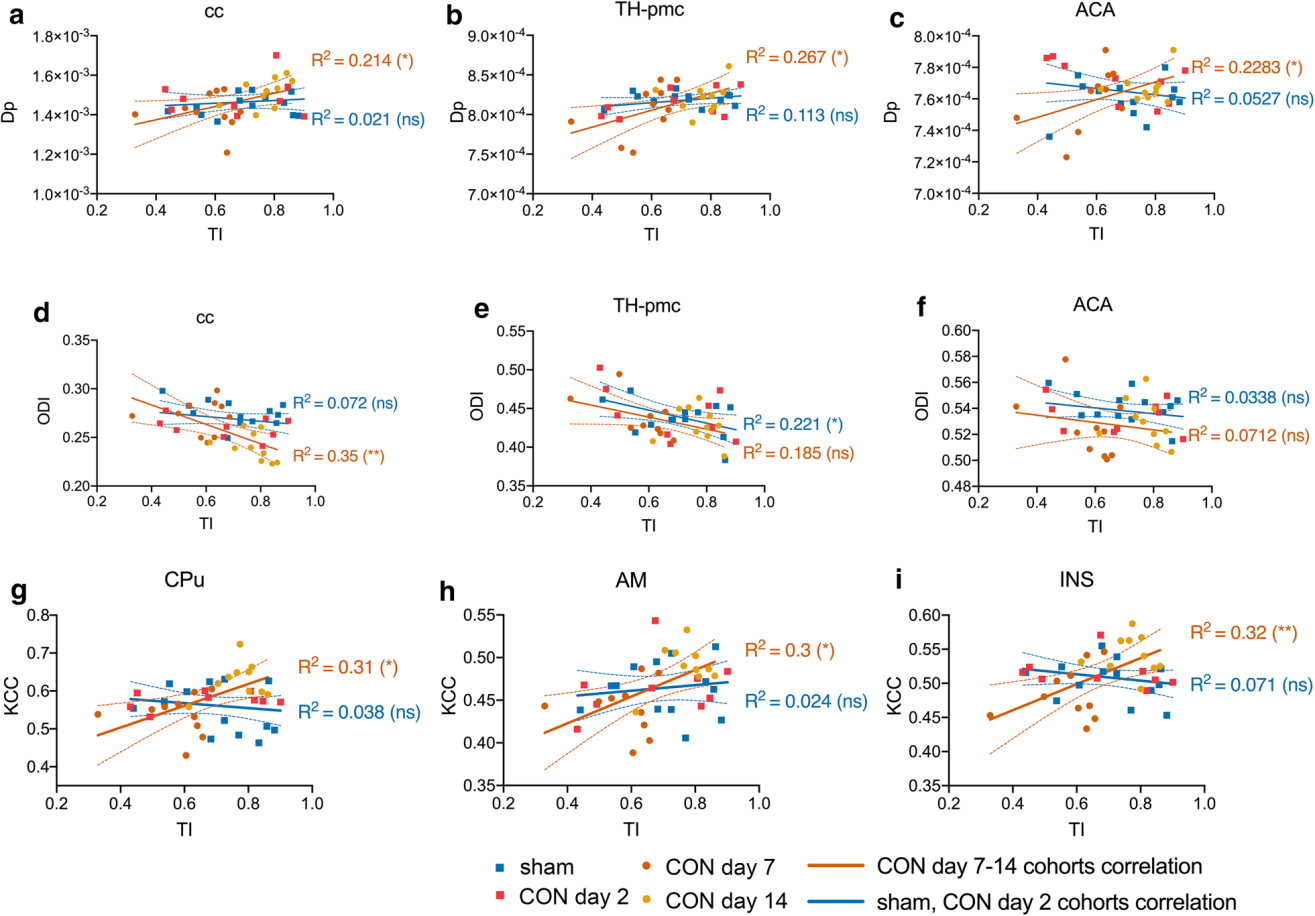
Correlation of diffusion and functional MRI with Open Field behavioural measures. Simple linear regression analysis of Parallel Diffusivity (Dp) (**a**–**c**), Orientation Dispersion Index (ODI) (**d**–**f**), and Kendall’s Coefficient of Concordance (KCC) from seven voxels neighbourhood Regional Homogeneity analysis of resting-state functional MRI (**g**–**i**), quantified from different regions of interest, the corpus callosum (cc—**a**, **d**), polymodal association area of the Thalamus (TH-pmc—**b**, **e**), Anterior Cingulate Area (ACA—**c**, **f**), Caudate Putamen (CPu—**g**), Amygdala (AM—**h**), and Insula (INS—**I**) with Thigmotaxis Index (TI). Regression analysis were performed across sham and CON day 2 cohorts combined (blue lines) or CON day 7 and CON day 14 combined (orange line). Regression lines displayed as mean and 95% confidence intervals slopes. ns = not significant, *p value < 0.05, **p value < 0.01

Voxel-wise correlation analysis defined three ROIs—cc, TH-pmc, and ACA of interest, that showed Dp were positively correlated and ODI were negatively correlated with TI. More detailed linear regression revealed that Dp and ODI in these ROIs were significantly correlated with TI only among CON day 7 and CON day 14 animals but not among the sham and CON day 2 animals (Fig. 4a–f), with the exception of ODI in the TH-pmc (Fig. 4e) and ACA (Fig. 4f). ODI was negatively correlated with TI among sham and CON day 2 cohorts but not among CON day 7 and day 14 cohort. ODI was not correlating with TI in the ACA.

Voxel-wise correlation analysis also defined three ROIs—CPu, AM, and INS that showed significant correlation between KCC and TI. More detailed analysis showed KCC were positively correlated with TI in all three ROIs among CON day 7 and CON day 14 animals but not among sham and CON day 2 cohorts (Fig. 4g–i).

## Discussion

This work shows a mismatch in the onset and recovery of the structural and functional attributes of the brain following a concussion, evidenced by advanced neuroimaging, beyond that of symptom subsidence. Motor deficits were evident immediately after concussion while deficits in learning were only evident at day 7; all symptoms resolved at day 14. Imaging findings varied; DTI and NODDI changes peaked at day 7 and significantly reduced at day 14 post-concussion while the functional connectivity increased at day 2 and 14 post-concussion. Additionally, stimulus-evoked fMRI detected no differences at day 2 but increased cortical activation at day 7 and 14 post-concussion.

### Behavioural changes following concussion

Motor-balance deficits were significant following a single concussive injury, as evidenced by lower NSS at day 2 post-concussion. Motor-balance symptoms partially recovered at day 7 and fully recovered at day 14. This recovery timeline of motor-balance symptoms in our model of rotational, acceleration/deceleration concussive injury is consistent with earlier studies of repeated or single mild impacts of the CHIMERA model, which showed injured animals recovered their motor balance function at approximately day 14 [77, 78].

Concussed animals displayed reduced TI and spent more time at the centre of the area compared to shams at day 7 post-concussion; this behaviour normalised at day 14. This trend of reduced TI and more time spent in the centre of the arena was consistent with similar results after a single moderate injury CHIMERA impact [79]. On the other hand, increased TI, anxiety-like behaviours, and less time spent at the centre of the arena during an open field task were observed at day 1 and day 7 after a single [78] or two consecutive mild CHIMERA impacts 24 h apart [77] and recovery occurred at day 14 [77]. The majority of other closed-head injury model studies also showed opposite trends of increased anxiety-like behaviours and decreased time spent in the centre of the arena at 2 days [80–82] or up to one month post-injury [20, 83]. Ertürk et al. observed no alternations from day 4 up to 8 weeks post-injury [84].

### Structural brain changes following concussion

Structural imaging and brain morphometry showed surprising dynamic post-concussion changes that correlated well with motor-balance symptoms (NSS) and anxiety-like behaviours (TI) to a lesser extent. At day 2 post-concussion, TBM analysis detected enlargement in the ventricles and several structures, including the auditory, visual, and S1, the hippocampus, anterior cingulate cortex, and the amygdala. Similar findings have been reported by human studies showing enlarged cortical structures in the short-term—from 24 h [85], 7 days [86] or up to 5.7 years post-concussion [87]. The majority of structural imaging studies in humans reported shrinkage in brain tissue volume after mild to moderate TBI (see Ross et al. [87] for review), though a recent study comparing a large number of mild to moderate TBI patients (n = 50) to the USA’s Food and Drug Administration-approved NeuroQuant^®^ normal control database [88, 89] revealed those patients have enlarged cortical grey matter, cerebellar white matter, and hippocampal volumes [87]. The short-term increased brain tissue volume and lack of long-term or permanent atrophy in our model can be explained by the lack of diffuse axonal injury; DTI and NODDI detecting no changes characteristic of diffuse axonal injury in our model and the correlation between diffuse axonal injury and brain atrophy after TBI were known in human TBI [90, 91]. Brain structural and morphological changes in this model occurred as early as 2 days post-injury and correlated well with behavioural symptoms. While this might seem very quick, this is consistent with our earlier study in the same model [40] and other studies which showed brain morphological changes could happen as early as 24-h post-intervention [92–94], ranging from changes in oestrous cycle [92], environmental enrichment [93], and maze training [94]; though the precise underlying mechanism is unclear.

### Diffusion MRI changes in the grey mater after concussion

Diffuse increases in FA in the grey matter of concussed animals were observed at day 7 post-injury. Increased FA in the grey matter is known to be associated with persistent post-concussive symptoms (PPCS) following a concussion in human patients [24], consistent with our results. Increased FA in the grey matter is also consistent with other in vivo animal studies [25–27]. Closer analysis showed this increased FA in the grey matter was primarily driven by a decrease in Dr and MD without as much decreased Dp. This pattern of elevated FA associated with reduced Dr and mostly unchanged Dp in the grey matter, is consistent with the majority of human PPCS showing elevated grey matter FA [24], and rat closed-head injury models [27]. A minority of human PPCS cases [24], rat open-head injury model [25] or repetitive mild blast exposure model [26] report elevated grey matter FA, which is often associated with increased Dp without changes in Dr. Regardless of the patterns of Dp or Dr changes, this elevated FA in the grey matter post-TBI has been associated with astrogliosis [25, 27] or microstructural remodelling in a rat model of open-head injury [95]. Of particular interest is the association of DTI/NODDI changes and astrocyte activity, since astrocytes have dual roles in neuronal plasticity and reconstruction after traumatic brain injury [96, 97].

### Diffusion MRI changes in the white mater after concussion

Increased FA and decreased ODI were detected in the white matter tracts at both day 7 and day 14 post-concussion in our model. Decreased FA in the white matter is commonly associated with reduced white matter integrity in Alzheimer’s disease [98], and in animal models of repeated mild TBI [17, 18]. Other single-impact rodent closed-head injuries also showed decreased FA in the white matter: drop-weight model in the rats [19] and piston-driven closed-head injury in the mice [20, 21] at 1 and 8 days post-injury [19] or up to a month [20], and 18 weeks post-injury [21]. Decreased FA and increased ODI were found in the optic tracts of a mouse model of closed-head injury at 1, 6, 12, and 18 weeks post-injury, even when memory deficits resolved within the first week post-injury; persistent neuroinflammation, including astrogliosis and microgliosis were associated with the DTI and NODDI changes in this model [21]. On the other hand, a rat model of a single mild modified controlled cortical impact showed no changes at day 2 post-injury but elevated FA and reduced MD and Dr in the corpus callosum and external capsule at day 7 post-injury—consistent with our results—though the changes normalised at day 14 [22]. Increased FA was also found in a rat drop weight model of mild traumatic brain injury 7 days post-injury [23].

In Mierzwa et al., demyelination of degenerating and intact axons was found at day 3 post-injury in a mouse model of a single mild closed-head injury, and was followed by remyelination and excessive myelination (including double-layered myelin sheaths) between day 7 and day 14 post-injury [99]. The process of remyelination of previously demyelinated axons in a cuprizone-fed mouse model of demyelination showed that the remyelination process decreased MD and Dr but no changes to Dp were observed [100]. The observed decreased Dr, MD, and ODI in our model at day 14 post-concussion suggested the possibility of the mice having excessive myelination in the corpus callosum as a result of injury repair/recovery. Increased FA and decreased MD have also been observed in non-injury neuroplasticity processes (a spatial learning task) involving increased myelination and myelin basic protein expression [101].

Currently, only a few NODDI studies in human concussion have been published to date and the results have been contradictory. Wu et al. [30] and Churchill et al. [31] detected decreased NDI in the white matter of concussed patients imaged no longer than 2 months post-injury. On the other hand, our patterns of increased NDI and decreased ODI in the white matter were consistent with Churchill et al. [32] where increased NDI and decreased ODI were detected in the white matter and grey matter-white matter boundaries of young, healthy athletes with a history of concussion (average of two concussions) imaged, on average, 24 months post-injury. In our study, increased FA, decreased ODI, and increased NDI were found in the white matter of concussed mice. These findings coupled with the relatively young age of the animals used (average age at impact or sham procedure 13.2 ± 1.4 weeks) suggested the age range and injury profile at day 7 and day 14 post-concussion of our animals fit with that of young, healthy humans imaged on average 24 months (and at least 9 months) post-concussion. Churchill et al. [32] suggested increased NDI and decreased ODI in the white matter of young, healthy and fit athletes post-concussion and without concussion symptoms was an indication that the axons recovering from injury were strengthened in the long-term after injury.

### Brain functional connectivity changes following concussions

Initially, there was an increase in functional connectivity between the M1 and the S1 upper limbs and increased local intrinsic connectivity in the S1, TH-pmc, and HP, SN, and the CPu. This increased long-range and local functional connectivity was normalised at day 7 post-concussion, when motor-balance deficits mostly subsided but psychological symptoms started. At day 14, all symptoms were normalised but increased long-range and local functional connectivity were found within the SN. Our results of initial increased functional connectivity associated with significant motor-balance deficits were consistent with some smaller studies that showed significant rsfMRI changes associated with post-concussion symptoms [34, 36, 37, 102]; the trends and networks most frequently involved in studies were reduced connectivity in the posterior DMN [34, 102], increased connectivity in the anterior DMN [34, 102], reduced anti-correlation among networks with anti-correlation relationships [102], and decreased local intrinsic functional connectivity in the SN [36, 37], in the lateralised cognitive control network [37], and in regions related with motor, sensorimotor, attention, and phonological processing [36]. On the other hand, Meier et al. reported short-term elevated local intrinsic functional connectivity in regions associated with the DMN that normalised upon symptom recovery [39]. Other studies in humans have also found persistent rsfMRI changes beyond symptom recovery [35, 103, 104], with one study reporting no significant changes to rsfMRI connectivity when symptoms were highest, but ongoing rsfMRI changes after symptoms had recovered [103]. Our results demonstrated persistent local and long-range functional connectivity changes at day 14 despite resolution of symptoms. Our findings of no functional connectivity changes and significant psychological symptoms at day 7 post-concussion, were also consistent with human studies reporting no rsfMRI changes despite significant ongoing symptoms as our results [38, 103].

A number of novel networks were defined in this study as part of the resting-state functional connectivity architecture through the patterns of mostly positive correlation among networks within the same supra-network and negative or anti-correlation among networks of different supra-network by contrast. These negative or anti-correlations were found in the absence of global signal regression during the pre-processing steps [105]. A number of human studies showed the persistence of anti-correlated connections, even without global signal regression, in ROI-based [106–108], graph theoretical [109], and independent component analysis [70]. Patterns of S2 cortex negative correlation with areas in the DMSM were found in mice [55] and rats [110]. The human SN, which is primarily comprised of the insula, amygdala, and dorsal anterior cingulate cortex [111], has been shown to also have anti-correlation with the human DMN [70, 112], The human Dorsal Attention Network is known to be another network that is anti-correlated with the human DMN [112]; thus, the S1BF and the S2 identified in the mouse brains may play an analogous role to the human Dorsal Attention Network.

### Multi-phase brain recovery after concussion

**Fig. 5:**
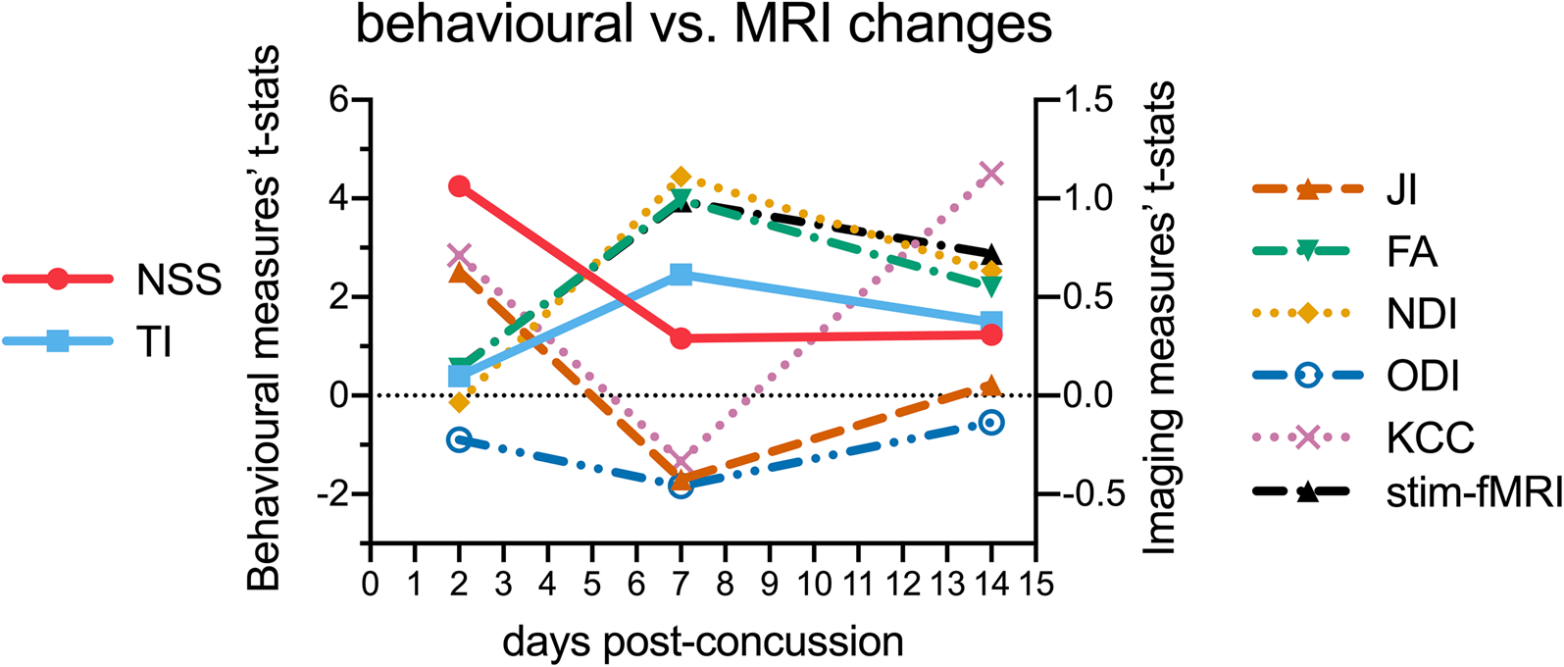
Deficit and recovery trajectories of behavioural and magnetic resonance imaging (MRI) markers post-concussion. Relative changes of behavioural and MRI metrics of concussed cohorts relative to the sham cohorts (horizontal black dotted line through 0). Behavioural measurements (NSS and TI) scaled as t-statistics of the corresponding CON versus sham comparison. MRI biomarkers (JI, FA, NDI, ODI, KCC, and stim-fMRI) scaled as whole-brain averaged *t*-statistics of the corresponding CON vesus sham comparison; whole-brain t-statistics were used as a biomarker proxy that incorporated both extents and degrees of change. NSS = Neuro Severity Score, TI = Thigmotaxis Index, JI = Jacobian Index, FA = Fractional Anisotropy, NDI = Neurite Density Index, ODI = Orientation Dispersion Index, KCC = Kendall’s Coefficient of Concordance (resting-state functional Magnetic Resonance Imaging Regional Homogeneity), and stim-fMRI (stimulus-evoked functional Magnetic Resonance Imaging). An alternative version of figure separating behavioural and MRI changes can be found in Supplementary data Fig. S4

We observed a biphasic pattern of neural recovery and plasticity post-injury, each with distinct patterns of behavioural symptoms and associated MRI findings (Fig. 5).

In the first phase of post-concussion recovery, up to day 7 post-injury, brain recovery was mostly related to functional compensation. In this phase, significant motor-balance symptoms were evident, which were associated with increased long-range and local functional connectivity with no concurrent changes in stimulus-evoked response detected with task-based fMRI; this can be interpreted as the brains’ increased activity to functionally compensate for the injury prior to injury recovery, healing, or plasticity [113].

In the second phase of concussion recovery, between day 7 post-injury and day 14, brain recovery was dominated by neural plasticity. In this phase, particularly at day 7 post-concussion, there was significant psychological symptoms in the mice, evidenced by the open field assessment, and DTI/NODDI detected changes consistent with astrogliosis and neuroinflammation [25]. Interestingly, the interaction of astrocytes with injured neurons has been shown to result in hyperexcitable neurons [96]. This change in cortical excitability was observed in a rat model of controlled cortical injury, with injured animals showing increased stimulus-evoked fMRI activation between 1 and 4 weeks post-injury [114]. Furthermore, Verley et al. showed bilateral hyperexcitability to a unilateral forepaw stimulation that was observed in the first week before functional reorganisation occurred and consolidated to unilateral hyperexcitability from week 2 to 4 [114]. Our stimulus-evoked fMRI results showed increased fMRI responses at day 7 and day 14 post-injury, and also demonstrated bilateral response enhancement in the CON day 7 cohort and unilateral enhancement in the CON day 14 cohort, suggesting the second phase of recovery being reflective of changes in neuroplasticity.

The post-concussion cortical hyperexcitability detectable with stimulus-evoked fMRI may explain the apparently contradictory trajectory of resting-state functional connectivity in our model through different timepoints. The increased functional connectivity at day 2 without concurrent cortical hyperexcitability possibly reflected functional compensation [113] without neural recovery/plasticity. The increased functional connectivity at day 14 with increasing cortical hyperexcitability might have been the result of ongoing neural plasticity in the brains [114]. NODDI patterns of increased NDI and decreased ODI at day 7 and 14 also supported hypothesis of neural plasticity occurring during this phase. Furthermore, DTI changes in the white matter also supports neuroplasticity processes that may include remyelination or excessive myelination.

### Limitations

The study suffers from a number of limitations. The behavioural assessments applied were restricted to a small number; rotarod can be used in addition to NSS to allow for more fine-grained scoring of motor-balance function/deficit [77], especially since the NSS range in this model was quite narrow. Learning and memory assessments, such as Barnes maze [115] should also be considered. In future studies, other behaviour tests that better assess depression, anxiety, irritability, aggression [116], and hyperarousal [117] symptoms should be included. The correlation of MRI biomarkers and the neurological processes that supposedly underlie the two brain recovery phases were hypothesised based on MRI biomarker fingerprinting of prior studies, often of different brain injury models. Nevertheless, the specific correlation of a histopathological process with a specific trend of diffusion imaging metric change is a complicated issue. For example, a specific pathology may result in a predictable biomarker on DTI or NODDI; however, different pathologies may create the same diffusion metric change [118]. Furthermore, in complex conditions like concussion or traumatic brain injury, pathologies generally do not occur alone. Astrogliosis, microgliosis, and axonal injury generally occur together as a consequence of concussive injury in mice [40, 77, 78, 115, 119]. As described in our earlier publication [40] DTI and NODDI metrics only correlated with microgliosis, similar to a NODDI examination of a model of microglia depletion and repopulation [120] in regions with just neuroinflammation. A more thorough investigation to include biological measures would be needed to further validate the findings in this work.

There are inherent limitations to extrapolating findings in animal models to human pathologies and TBI models are of no exception. There are differences in gross anatomy between rodents and humans (lack of gyri/sulci in rodents) and rotational force component common in large-brained humans TBIs are difficult to be replicated in small-brained animals [121]. Tauopathy is a significant feature in repetitive closed head injury (for example, contact sport athletes and military personnel) [122], but TBI-induced tauopathy in rodents have mainly been demonstrated in transgenic mice, which were genetically modified to develop tauopathy [121]. Other studies in the CHIMERA model showed tau phosphorylation to be a transient feature, which normalised by day 7 post-injury [77, 115]. It is notable that in the CHIMERA model, two consecutive mild impacts 23 h apart caused increased tau phosphorylation lasting until day 2 post-injury while a single moderate-severe impact only cause transient increased tau-phosphorylation at 6 h post-injury. Assuming the brains can completely recover after an injury, our imaging study attempted to identify imaging biomarkers for injury recovery and, more importantly, a window of vulnerability within which consecutive injuries may cause disproportionate consequences, for example, long-term tauopathy. Chronic traumatic encephalopathy has been associated with sub-populations at risk of repetitive traumatic brain injury, such as contact sport athletes and military personnel [123] while animal models of repetitive closed-head injury do not consistently demonstrate only transient elevated phospho-tau [124]. Long-term elevated phosphor-tau, 6 months post-injury, was shown in one study after as many as 42 impacts [125]. More research is required at this stage to address the question of human pathology can be modelled in animals and the existence of a hypothetical window of vulnerability.

## Conclusion

Our multi-modal assessment of a mouse model of concussion showed varying trends in the temporal profile of different MRI markers. While concussion symptoms and routine structural imaging found significant changes up to day 7 post-concussion, the changes were normalised by day 14. By contrast, advanced imaging using DTI, NODDI, resting-state, and stimulus-evoked fMRI revealed ongoing changes that persisted at day 14 with different onset, reflecting ongoing biological and molecular changes. To the best of the authors’ knowledge, this study represents the first study to perform multi-modal advanced MRI and behavioural assessment to monitor recovery after a concussion in a mouse model. The findings in this study have important implication for translation to human clinical settings. First, recovery and discharge by clinical assessment criteria may not indicate complete brain recovery and the brain may still be vulnerable to disproportionate consequences from subsequent concussions. Second, it is likely that multi-modal assessments using different imaging methods will provide a more complete clinical picture in human concussions.

## Supplementary Information

**Additional file 1: Data 1.** “Signal” resting-state Group Independent Vector Analysis components.

**Additional file 2: Figure S2.** Group averaged resting-state MRI functional connectivity. (A–D) Average functional network connectivity (FNC) matrices among Independent Components (ICs) identified by IVA-GL of (A) sham (n = 14), (B) CON day 2 (n = 9), (C) CON day 7 (n = 10), and (D) CON day 14 (n = 10) cohorts. Colour scaled by z test statistics; non-black cells were defined as component-component connectivity deemed statistically significant. One sample t-tests, permutation-tested, and FDR-corrected (q-value < 0.05, two-tailed). (E–H) Regional Homogeneity analysis’s local intrinsic functional connectivity represented as averaged seven-voxels-neighbourhood Kendall’s Coefficient of Concordance (KCC) maps of (E) sham, (F) CON day 2, (G) CON day 7, and (H) CON day 14 cohorts.

**Additional file 3: Figure S3.** Randomise reproducibility test of DTI and NODDI metrics. Voxel-by-voxel statistical analysis results of Diffusion Tensor Imaging (FA = Fractional Anisotropy, MD = Mean Diffusivity, Dp = Parallel Diffusivity, Dr = Radial Diffusivity) and Neurite Orientation Dispersion and Density Imaging metrics (NDI = Neurite Density Index, ODI = Orientation Dispersion Index) and Tensor-based Morphometry with Jacobian Index (JI) reproducibility test. Statistical map thresholded at P value < 0.05 (two-tailed), unpaired two sample t-test, implemented as permutation tested for the General Linear Model, corrected for multiple comparisons with mass-based FSL’s Threshold-free Cluster enhancement (TFCE). Statistical maps were overlaid on the averaged and registered DTI and NODDI metrics maps corresponding to the statistical maps (DTI and NODDI results) and structural template (TBM results). Corresponding grey scale map for each averaged DTI and NODDI metrics maps were provided; units for Dp, Dr, and MD were in mm/s2. ACA = Anterior Cingulate Area, AM = Amygdala, AUD = Auditory Area, cc = corpus callosum, ec = external capsule, HP = Hippocampus, HYP = Hypothalamus, ic = internal capsule, INS = Insula, MB = Midbrain, PAL = Palladium, S1 = Primary Somatosensory Cortex, RSN = Retrosplenial Area, STR = Striatum, TH = Thalamus, VIS = Visual Area.

**Additional file 4: Figure S4.** Deficit and recovery trajectories of behavioural and Magnetic Resonance Imaging (MRI) markers post-concussion. Relative changes of (A) behavioural and (B) MRI metrics of concussed cohorts relative to the sham cohorts (horizontal black dotted line through 0). Behavioural measurements (NSS and TI) scaled as t-statistics of the corresponding CON vs. sham comparison. MRI biomarkers (JI, FA, NDI, ODI, KCC, and stim-fMRI) scaled as whole-brain averaged t-statistics of the corresponding CON vs. sham comparison; whole-brain t-statistics were used as a biomarker proxy that incorporated both extents and degrees of change. NSS = Neuro Severity Score, TI = Thigmotaxis Index, JI = Jacobian Index, FA = Fractional Anisotropy, NDI = Neurite Density Index, ODI = Orientation Dispersion Index, KCC = Kendall’s Coefficient of Concordance (resting-state functional Magnetic Resonance Imaging Regional Homogeneity), and stim-fMRI (stimulus-evoked functional Magnetic Resonance Imaging).

## Data Availability

Data from this study is available, without reservations, on request to the corresponding author.

## References

[1] National Center for Injury Prevention and Control (2003) Report to Congress on mild traumatic brain injury in the United States: steps to prevent a serious public health problem. Centers for Disease Control and Prevention, Atlanta, GA

[2] Williams RM, Puetz TW, Giza CC, Broglio SP (2015). Concussion recovery time among high school and collegiate athletes: a systematic review and meta-analysis. Sport Med.

[3] Henry LC, Elbin RJ, Collins MW, Marchetti G, Kontos AP (2016). Examining recovery trajectories following sport-related concussion using a multi-modal clinical assessment approach. J Neurosurg.

[4] Semple BD, Lee S, Sadjadi R, Fritz N, Carlson J, Griep C (2015). Repetitive concussions in adolescent athletes—Translating clinical and experimental research into perspectives on rehabilitation strategies. Front Neurol.

[5] Stejskal EO, Tanner JE (1965). Spin diffusion measurements: spin echoes in the presence of a time-dependent field gradient. J Chem Phys.

[6] Basser PJ (1995). Inferring microstructural features and the physiological state of tissues from diffusion weighted images. NMR Biomed.

[7] O’Donnell LJ, Westin C-F (2011) An introduction to diffusion tensor image analysis. Neurosurg Clin N Am 22:185–96, viii 10.1016/j.nec.2010.12.004 PMC3163395 21435570

[8] Alexander AL, Lee JE, Lazar M, Field AS (2007). Diffusion tensor imaging of the brain. Neurotherapeutics.

[9] Miles L, Grossman RI, Johnson G, Babb JS, Diller L, Inglese M (2008). Short-term DTI predictors of cognitive dysfunction in mild traumatic brain injury. Brain Inj.

[10] Aoki Y, Inokuchi R, Gunshin M, Yahagi N, Suwa H (2012). Diffusion tensor imaging studies of mild traumatic brain injury: a meta-analysis. J Neurol Neurosurg Psychiatry.

[11] Brandstack N, Kurki T, Tenovuo O (2013). Quantitative diffusion-tensor tractography of long association tracts in patients with traumatic brain injury without associated findings at routine MR imaging. Radiology.

[12] Yuh EL, Cooper SR, Mukherjee P, Yue JK, Lingsma HF, Gordon WA (2014). Diffusion Tensor Imaging for outcome prediction in mild traumatic brain Injury: a TRACK-TBI study. J Neurotrauma.

[13] Bazarian JJ, Zhong J, Blyth B, Zhu T, Kavcic V, Peterson D (2007). Diffusion tensor imaging detects clinically important axonal damage after mild traumatic brain injury: a pilot study. J Neurotrauma.

[14] Mayer AR, Ling J, Mannell MV, Gasparovic C, Phillips JP, Doezema D (2010). A prospective diffusion tensor imaging study in mild traumatic brain injury. Neurology.

[15] Ling JM, Peña A, Yeo RA, Merideth FL, Klimaj S, Gasparovic C (2012). Biomarkers of increased diffusion anisotropy in semi-acute mild traumatic brain injury: a longitudinal perspective. Brain.

[16] Eierud C, Craddock RC, Fletcher S, Aulakh M, King-Casas B, Kuehl D (2014). Neuroimaging after mild traumatic brain injury: review and meta-analysis. NeuroImage Clin.

[17] Wortman RC, Meconi A, Neale KJ, Brady RD, McDonald SJ, Christie BR (2018). Diffusion MRI abnormalities in adolescent rats given repeated mild traumatic brain injury. Ann Clin Transl Neurol.

[18] Haber M, Hutchinson EB, Sadeghi N, Cheng WH, Namjoshi D, Cripton P et al (2017) Defining an analytic framework to evaluate quantitative MRI markers of traumatic axonal injury: preliminary results in a mouse closed head injury model. eNeuro 4:ENEURO.0164-17.2017 10.1523/ENEURO.0164-17.2017 PMC5616192 28966972

[19] Tu T-W, Lescher JD, Williams RA, Jikaria N, Turtzo LC, Frank JA (2017). Abnormal injury response in spontaneous mild ventriculomegaly Wistar rat brains: a pathological correlation study of diffusion tensor and magnetization transfer imaging in mild traumatic brain injury. J Neurotrauma.

[20] Rodriguez-Grande B, Obenaus A, Ichkova A, Aussudre J, Bessy T, Barse E (2018). Gliovascular changes precede white matter damage and long-term disorders in juvenile mild closed head injury. Glia.

[21] Gazdzinski LM, Mellerup M, Wang T, Adel SAA, Lerch JP, Sled JG (2020). White matter changes caused by mild traumatic brain injury in mice evaluated using neurite orientation dispersion and density imaging. J Neurotrauma.

[22] Hoogenboom WS, Rubin TG, Ye K, Cui MH, Branch KC, Liu J (2019). Diffusion tensor imaging of the evolving response to mild traumatic brain injury in rats. J Exp Neurosci.

[23] Kikinis Z, Muehlmann M, Pasternak O, Peled S, Kulkarni P, Ferris C (2017). Diffusion imaging of mild traumatic brain injury in the impact accelerated rodent model: a pilot study. Brain Inj.

[24] Bouix S, Pasternak O, Rathi Y, Pelavin PE, Zafonte R, Shenton ME (2013). Increased gray matter diffusion anisotropy in patients with persistent post-concussive symptoms following mild traumatic brain injury. PLoS One.

[25] Budde MD, Janes L, Gold E, Turtzo LC, Frank JA (2011). The contribution of gliosis to diffusion tensor anisotropy and tractography following traumatic brain injury: validation in the rat using Fourier analysis of stained tissue sections. Brain.

[26] Badea A, Kamnaksh A, Anderson RJ, Calabrese E, Long JB, Agoston DV (2018). Repeated mild blast exposure in young adult rats results in dynamic and persistent microstructural changes in the brain. NeuroImage Clin.

[27] Braeckman K, Descamps B, Pieters L, Vral A, Caeyenberghs K, Vanhove C (2019). Dynamic changes in hippocampal diffusion and kurtosis metrics following experimental mTBI correlate with glial reactivity. NeuroImage Clin.

[28] Zhang H, Schneider T, Wheeler-Kingshott CA, Alexander DC (2012). NODDI: practical in vivo neurite orientation dispersion and density imaging of the human brain. Neuroimage.

[29] Tariq M, Schneider T, Alexander DC, Gandini Wheeler-Kingshott CA, Zhang H (2016). Bingham-NODDI: mapping anisotropic orientation dispersion of neurites using diffusion MRI. Neuroimage.

[30] Wu Y-C, Mustafi SM, Harezlak J, Kodiweera C, Flashman LA, McAllister TW (2018). Hybrid diffusion imaging in mild traumatic brain injury. J Neurotrauma.

[31] Churchill NW, Caverzasi E, Graham SJ, Hutchison MG, Schweizer TA (2019). White matter during concussion recovery: comparing diffusion tensor imaging (DTI) and neurite orientation dispersion and density imaging (NODDI). Hum Brain Mapp.

[32] Churchill NW, Caverzasi E, Graham SJ, Hutchison MG, Schweizer TA (2017). White matter microstructure in athletes with a history of concussion: comparing diffusion tensor imaging (DTI) and neurite orientation dispersion and density imaging (NODDI). Hum Brain Mapp.

[33] Biswal B, Yetkin FZ, Haughton VM, Hyde JS (1995). Functional connectivity in the motor cortex of resting human brain using echo-planar MRI. Magn Reson Med.

[34] Zhou Y, Milham MP, Lui YW, Miles L, Reaume J, Sodickson DK (2012). Default-mode network disruption in mild traumatic brain injury. Radiology.

[35] Johnson B, Zhang K, Gay M, Horovitz S, Hallet M, Sebastianelli W (2012). Alteration of brain default network in subacute phase of injury in concussed individuals: resting-state fMRI study. Neuroimage.

[36] Zhan J, Gao L, Zhou F, Kuang H, Zhao J, Wang S (2015). Decreased regional homogeneity in patients with acute mild traumatic brain injury: a resting-state fMRI Study. J Nerv Ment Dis.

[37] Peters SK, Dunlop K, Downar J (2016). Cortico-striatal-thalamic loop circuits of the salience network: a central pathway in psychiatric disease and treatment. Front Syst Neurosci.

[38] Kaushal M, España LY, Nencka AS, Wang Y, Nelson LD, McCrea MA (2019). Resting-state functional connectivity after concussion is associated with clinical recovery. Hum Brain Mapp.

[39] Meier TB, Giraldo-Chica M, España LY, Mayer AR, Harezlak J, Nencka AS (2020). Resting-state fMRI metrics in acute sport-related concussion and their association with clinical recovery: a study from the NCAA-DOD CARE consortium. J Neurotrauma.

[40] To XV, Benetatos J, Soni N, Liu D, Abraha HM, Yan W et al (2020) Ultra-high field diffusion tensor imaging identifies discrete patterns of concussive injury in the rodent brain. J Neurotrauma 2020;neu.2019.6944 10.1089/neu.2019.6944 32394788

[41] Flierl MA, Stahel PF, Beauchamp KM, Morgan SJ, Smith WR, Shohami E (2009). Mouse closed head injury model induced by a weight-drop device. Nat Protoc.

[42] Simon P, Dupuis R, Costentin J (1994). Thigmotaxis as an index of anxiety in mice Influence of dopaminergic transmissions. Behav Brain Res.

[43] Rorden C, Brett M (2000). Stereotaxic display of brain lesions. Behav Neurol.

[44] Bajic D, Craig MM, Mongerson CRL, Borsook D, Becerra L (2017) Identifying rodent resting-state brain networks with independent component analysis. Front Neurosci 11 10.3389/fnins.2017.00685 PMC5733053 29311770

[45] Smith SM, Jenkinson M, Woolrich MW, Beckmann CF, Behrens TEJ, Johansen-Berg H (2004). Advances in functional and structural MR image analysis and implementation as FSL. Neuroimage.

[46] Ashburner J, Friston KJ, Frackowiak RSJ, Friston KJ, Frith C, Dolan R, Friston KJ, Price CJ (2003). Morphometry. Human brain function.

[47] Andersson JLR, Skare S, Ashburner J (2003). How to correct susceptibility distortions in spin-echo echo-planar images: application to diffusion tensor imaging. Neuroimage.

[48] Hong X, To XV, Teh I, Rui J, Chuang K (2015). Evaluation of EPI distortion correction methods for quantitative MRI of the brain at high magnetic field. Magn Reson Imaging.

[49] Dohmatob E, Varoquaux G, Thirion B (2018). Inter-subject registration of functional images: do we need anatomical images?. Front Neurosci.

[50] Du Y, Fan Y (2013). Group information guided ICA for fMRI data analysis. Neuroimage.

[51] Calhoun VD, Adali T, Pearlson GD, Pekar JJ (2001). A method for making group inferences from functional MRI data using independent component analysis. Hum Brain Mapp.

[52] Smith SM, Andersson J, Auerbach EJ, Beckmann CF, Bijsterbosch J, Douaud G (2013). Resting-state fMRI in the human connectome project. Neuroimage.

[53] Du Y, Allen EA, He H, Sui J, Wu L, Calhoun VD (2016). Artifact removal in the context of group ICA: a comparison of single-subject and group approaches. Hum Brain Mapp.

[54] Gri L, Douaud G, Bijsterbosch J, Evangelisti S, Alfaro-almagro F, Glasser MF (2017). Hand classification of fMRI ICA noise components. Neuroimage.

[55] Zerbi V, Grandjean J, Rudin M, Wenderoth N (2015). Mapping the mouse brain with rs-fMRI: an optimized pipeline for functional network identification. Neuroimage.

[56] Bell AJ, Sejnowski TJ (1995). An information-maximization approach to blind separation and blind deconvolution. Neural Comput.

[57] Lee T, Girolami M, Sejnowski TJ (1999). Independent component analysis using an extended infomax algorithm for mixed subgaussian and supergaussian sources. Neural Comput.

[58] Nasrallah FA, Tay H, Chuang K (2014). Detection of functional connectivity in the resting mouse brain. Neuroimage.

[59] Anderson M, Adali T, Li X-L (2012). Joint blind source separation swith multivariate gaussian model: algorithms and performance analysis. IEEE Trans Signal Process.

[60] Lee JH, Lee TW, Jolesz FA, Yoo SS (2008). Independent vector analysis (IVA): multivariate approach for fMRI group study. Neuroimage.

[61] Michael AM, Anderson M, Miller RL, Adalı T, Calhoun VD (2014). Preserving subject variability in group fMRI analysis: performance evaluation of GICA vs. IVA. Front Syst Neurosci.

[62] Laney J, Westlake K, Ma S, Woytowicz E, Calhoun VD, Adali T (2015). Capturing subject variability in fMRI data: a graph-theoretical analysis of GICA vs. IVAIVA. J Neurosci Methods.

[63] Du Y, Lin D, Yu Q, Sui J, Chen J, Rachakonda S (2017). Comparison of IVA and GIG-ICA in brain functional network estimation using fMRI data. Front Neurosci.

[64] Zang Y, Jiang T, Lu Y, He Y, Tian L (2004). Regional homogeneity approach to fMRI data analysis. Neuroimage.

[65] Szafer A, Zhong J, Gore JC (1995). Theoretical model for water diffusion in tissues. Magn Reson Med.

[66] Anderson MJ, Robinson J (2001). Permutation tests for linear models. Aust New Zeal J Stat.

[67] Winkler AM, Ridgway GR, Webster MA, Smith SM, Nichols TE (2014). Permutation inference for the general linear model. Neuroimage.

[68] Dickie DA, Mikhael S, Job DE, Wardlaw JM, Laidlaw DH, Bastin ME (2015). Permutation and parametric tests for effect sizes in voxel-based morphometry of gray matter volume in brain structural MRI. Magn Reson Imaging.

[69] Smith SM, Nichols TE (2009). Threshold-free cluster enhancement: addressing problems of smoothing, threshold dependence and localisation in cluster inference. Neuroimage.

[70] Allen EA, Erhardt EB, Damaraju E, Gruner W, Segall JM, Silva RF (2011). A baseline for the multivariate comparison of resting-state networks. Front Syst Neurosci.

[71] Gozzi A, Schwarz AJ (2016). Large-scale functional connectivity networks in the rodent brain. Neuroimage.

[72] Hsu L-M, Liang X, Gu H, Brynildsen JK, Stark JA, Ash JA (2016). Constituents and functional implications of the rat default mode network. Proc Natl Acad Sci.

[73] Jankowski MM, Ronnqvist KC, Tsanov M, Vann SD, Wright NF, Erichsen JT (2013). The anterior thalamus provides a subcortical circuit supporting memory and spatial navigation. Front Syst Neurosci.

[74] O’Mara SM, Aggleton JP (2019). Space and memory (far) beyond the hippocampus: many subcortical structures also support cognitive mapping and mnemonic processing. Front Neural Circuits.

[75] Bubb EJ, Kinnavane L, Aggleton JP (2017). Hippocampal–diencephalic–cingulate networks for memory and emotion: an anatomical guide. Brain Neurosci Adv.

[76] Grandjean J, Zerbi V, Balsters J, Wenderoth N, Rudina M (2017). The structural basis of large-scale functional connectivity in the mouse. J Neurosci.

[77] Namjoshi DR, Cheng WH, McInnes KA, Martens KM, Carr M, Wilkinson A (2014). Merging pathology with biomechanics using CHIMERA (closed-head impact model of engineered rotational acceleration): a novel, surgery-free model of traumatic brain injury. Mol Neurodegener.

[78] Namjoshi DR, Cheng WH, Bashir A, Wilkinson A, Stukas S, Martens KM (2017). Defining the biomechanical and biological threshold of murine mild traumatic brain injury using CHIMERA (closed head impact model of engineered rotational acceleration). Exp Neurol.

[79] Sauerbeck AD, Fanizzi C, Kim JH, Gangolli M, Bayly PV, Wellington CL, Brody DL, Kummer TT (2018). modCHIMERA: a novel murine closed-head model of moderate traumatic brain injury. Sci Rep.

[80] Hehar H, Yu K, Ma I, Mychasiuk R (2016). Paternal age and diet: the contributions of a father’s experience to susceptibility for post-concussion symptomology. Neuroscience.

[81] Salberg S, Yamakawa G, Christensen J, Kolb B, Mychasiuk R (2017). Assessment of a nutritional supplement containing resveratrol, prebiotic fiber, and omega-3 fatty acids for the prevention and treatment of mild traumatic brain injury in rats. Neuroscience.

[82] Mychasiuk R, Hehar H, Candy S, Ma I, Esser MJ (2016). The direction of the acceleration and rotational forces associated with mild traumatic brain injury in rodents effect behavioural and molecular outcomes. J Neurosci Methods.

[83] Sherman M, Liu MM, Birnbaum S, Wolf SE, Minei JP, Gatson JW (2016). Adult obese mice suffer from chronic secondary brain injury after mild TBI. J Neuroinflamm.

[84] Ertürk A, Mentz S, Stout EE, Hedehus M, Dominguez SL, Neumaier L (2016). Interfering with the chronic immune response rescues chronic degeneration after traumatic brain injury. J Neurosci.

[85] Govindarajan KA, Narayana PA, Hasan KM, Wilde EA, Levin HS, Hunter JV (2016). Cortical thickness in mild traumatic brain injury. J Neurotrauma.

[86] Wang X, Xie H, Cotton AS, Tamburrino MB, Brickman KR, Lewis TJ (2015). Early cortical thickness change after mild traumatic brain injury following motor vehicle collision. J Neurotrauma.

[87] Ross DE, Seabaugh JD, Seabaugh JM, Alvarez C, Ellis LP, Powell C (2020). Patients with chronic mild or moderate traumatic brain injury have abnormal brain enlargement. Brain Inj.

[88] Ross DE, Graham TJ, Ochs AL (2013). Review of the evidence supporting the medical and legal use of NeuroQuant^®^ in patients with traumatic brain injury. Psychol Inj Law.

[89] Ross DE, Seabaugh J, Cooper L, Seabaugh J (2018). NeuroQuant^®^ and NeuroGage^®^ reveal effects of traumatic brain injury on brain volume. Brain Inj.

[90] Ding K, De La Plata CM, Wang JY, Mumphrey M, Moore C, Harper C (2008). Cerebral atrophy after traumatic white matter injury: correlation with acute neuroimaging and outcome. J Neurotrauma.

[91] Scheid R, Preul C, Gruber O, Wiggins C, von Cramon DY (2003). Diffuse axonal injury associated with chronic traumatic brain injury: evidence from T2*-weighted gradient-echo imaging at 3 T. AJNR Am J Neuroradiol.

[92] Qiu LR, Germann J, Spring S, Alm C, Vousden DA, Palmert MR (2013). Hippocampal volumes differ across the mouse estrous cycle, can change within 24 hours, and associate with cognitive strategies. Neuroimage.

[93] Scholz J, Allemang-Grand R, Dazai J, Lerch JP (2015). Environmental enrichment is associated with rapid volumetric brain changes in adult mice. Neuroimage.

[94] Lerch JP, Yiu AP, Martinez-Canabal A, Pekar T, Bohbot VD, Frankland PW (2011). Maze training in mice induces MRI-detectable brain shape changes specific to the type of learning. Neuroimage.

[95] Li L, Chopp M, Ding G, Qu C, Nejad-Davarani SP, Davoodi-Bojd E (2017). Diffusion-derived magnetic resonance imaging measures of longitudinal microstructural remodeling induced by marrow stromal cell therapy after traumatic brain injury. J Neurotrauma.

[96] Burda JE, Bernstein AM, Sofroniew MV (2016). Astrocyte roles in traumatic brain injury. Exp Neurol.

[97] Zhou Y, Shao A, Yao Y, Tu S, Deng Y, Zhang J (2020). Dual roles of astrocytes in plasticity and reconstruction after traumatic brain injury. Cell Commun Signal.

[98] Kantarci K, Murray ME, Schwarz CG, Reid RI, Przybelski SA, Lesnick T (2017). White-matter integrity on DTI and the pathologic staging of Alzheimer’s disease. Neurobiol Aging.

[99] Mierzwa AJ, Marion CM, Sullivan GM, McDaniel DP, Armstrong RC (2015). Components of myelin damage and repair in the progression of white matter pathology after mild traumatic brain injury. J Neuropathol Exp Neurol.

[100] Wang N, Zhuang J, Wei H, Dibb R, Qi Y, Liu C (2019). Probing demyelination and remyelination of the cuprizone mouse model using multimodality MRI. J Magn Reson Imaging.

[101] Blumenfeld-Katzir T, Pasternak O, Dagan M, Assaf Y (2011). Diffusion MRI of structural brain plasticity induced by a learning and memory task. PLoS One.

[102] Sours C, Zhuo J, Janowich J, Aarabi B, Shanmuganathan K, Gullapalli RP (2013). Default mode network interference in mild traumatic brain injury—A pilot resting state study. Brain Res.

[103] McCuddy WT, España LY, Nelson LD, Birn RM, Mayer AR, Meier TB (2018). Association of acute depressive symptoms and functional connectivity of emotional processing regions following sport-related concussion. NeuroImage Clin.

[104] Meier TB, Bellgowan PSF, Mayer AR (2017). Longitudinal assessment of local and global functional connectivity following sports-related concussion. Brain Imaging Behav.

[105] Murphy K, Birn RM, Handwerker DA, Jones TB, Bandettini PA (2009). The impact of global signal regression on resting state correlations: are anti-correlated networks introduced?. Neuroimage.

[106] Chang C, Glover GH (2009). Effects of model-based physiological noise correction on default mode network anti-correlations and correlations. Neuroimage.

[107] Fransson P (2005). Spontaneous low-frequency BOLD signal fluctuations: an fMRI investigation of the resting-state default mode of brain function hypothesis. Hum Brain Mapp.

[108] Uddin LQ, Clare Kelly AM, Biswal BB, Xavier Castellanos F, Milham MP (2009). Functional connectivity of default mode network components: correlation, anticorrelation, and causality. Hum Brain Mapp.

[109] Gopinath K, Krishnamurthy V, Cabanban R, Crosson BA (2015). Hubs of anticorrelation in high-resolution resting-state functional connectivity network architecture. Brain Connect.

[110] Schwarz AJ, Gass N, Sartorius A, Risterucci C, Spedding M, Schenker E (2013). Anti-correlated cortical networks of intrinsic connectivity in the rat brain. Brain Connect.

[111] Akiki TJ, Averill CL, Abdallah CG (2017). A network-based neurobiological model of PTSD: evidence from structural and functional neuroimaging studies. Curr Psychiatry Rep.

[112] Raichle ME (2011). The restless brain. Brain Connect.

[113] Iraji A, Chen H, Wiseman N, Welch RD, O’Neil BJ, Haacke EM et al (2016) Compensation through functional hyperconnectivity: a longitudinal connectome assessment of mild traumatic brain injury. Neural Plast 10.1155/2016/4072402 PMC4706919 26819765

[114] Verley DR, Torolira D, Pulido B, Gutman B, Bragin A, Mayer A (2018). Remote changes in cortical excitability after experimental traumatic brain injury and functional reorganization. J Neurotrauma.

[115] Bashir A, Abebe ZA, McInnes KA, Button EB, Tatarnikov I, Cheng WH (2020). Increased severity of the CHIMERA model induces acute vascular injury, sub-acute deficits in memory recall, and chronic white matter gliosis. Exp Neurol.

[116] Malkesman O, Tucker LB, Ozl J, McCabe JT (2013). Traumatic brain injury—modeling neuropsychiatric symptoms in rodents. Front Neurol.

[117] Golub Y, Mauch CP, Dahlhoff M, Wotjak CT (2009). Consequences of extinction training on associative and non-associative fear in a mouse model of Posttraumatic Stress Disorder (PTSD). Behav Brain Res.

[118] Jelescu IO, Budde MD (2017). Design and validation of diffusion MRI models of white matter. Front Phys.

[119] Namjoshi DR, Cheng WH, Carr M, Martens KM, Zareyan S, Wilkinson A (2016). Chronic exposure to androgenic-anabolic steroids exacerbates axonal injury and microgliosis in the CHIMERA mouse model of repetitive concussion. PLoS ONE.

[120] Yi SY, Barnett BR, Torres-Velázquez M, Zhang Y, Hurley SA, Rowley PA (2019). Detecting microglial density With quantitative multi-compartment diffusion MRI. Front Neurosci.

[121] Risling M, Smith D, Stein TD, Thelin EP, Zanier ER, Ankarcrona M (2019). Modelling human pathology of traumatic brain injury in animal models. J Intern Med.

[122] Castellani RJ, Perry G, Tabaton M (2019). Tau biology, tauopathy, traumatic brain injury, and diagnostic challenges. J Alzheimer’s Dis.

[123] Asken BM, Sullan MJ, DeKosky ST, Jaffee MS, Bauer RM (2017). Research gaps and controversies in chronic traumatic encephalopathy: a review. JAMA Neurol.

[124] Hoogenboom WS, Branch CA, Lipton ML (2019). Animal models of closed-skull, repetitive mild traumatic brain injury. Pharmacol Ther.

[125] Petraglia A, Plog B, Dayawansa S, Dashnaw M, Czerniecka K, Walker C (2014). The pathophysiology underlying repetitive mild traumatic brain injury in a novel mouse model of chronic traumatic encephalopathy. Surg Neurol Int.

