# Supplementary material for: A roadmap of brain recovery in a mouse model of concussion: insights from neuroimaging
Source: Acta Neuropathol Commun. 2021 Jan 6;9:2. doi: 10.1186/s40478-020-01098-y (PMC7789702; doi:10.1186/s40478-020-01098-y)
Supplement: Supplementary file 1 — Additional file 1: Data 1. “Signal” resting-state Group Independent Vector Analysis components. [file 40478_2020_1098_MOESM1_ESM.pdf]

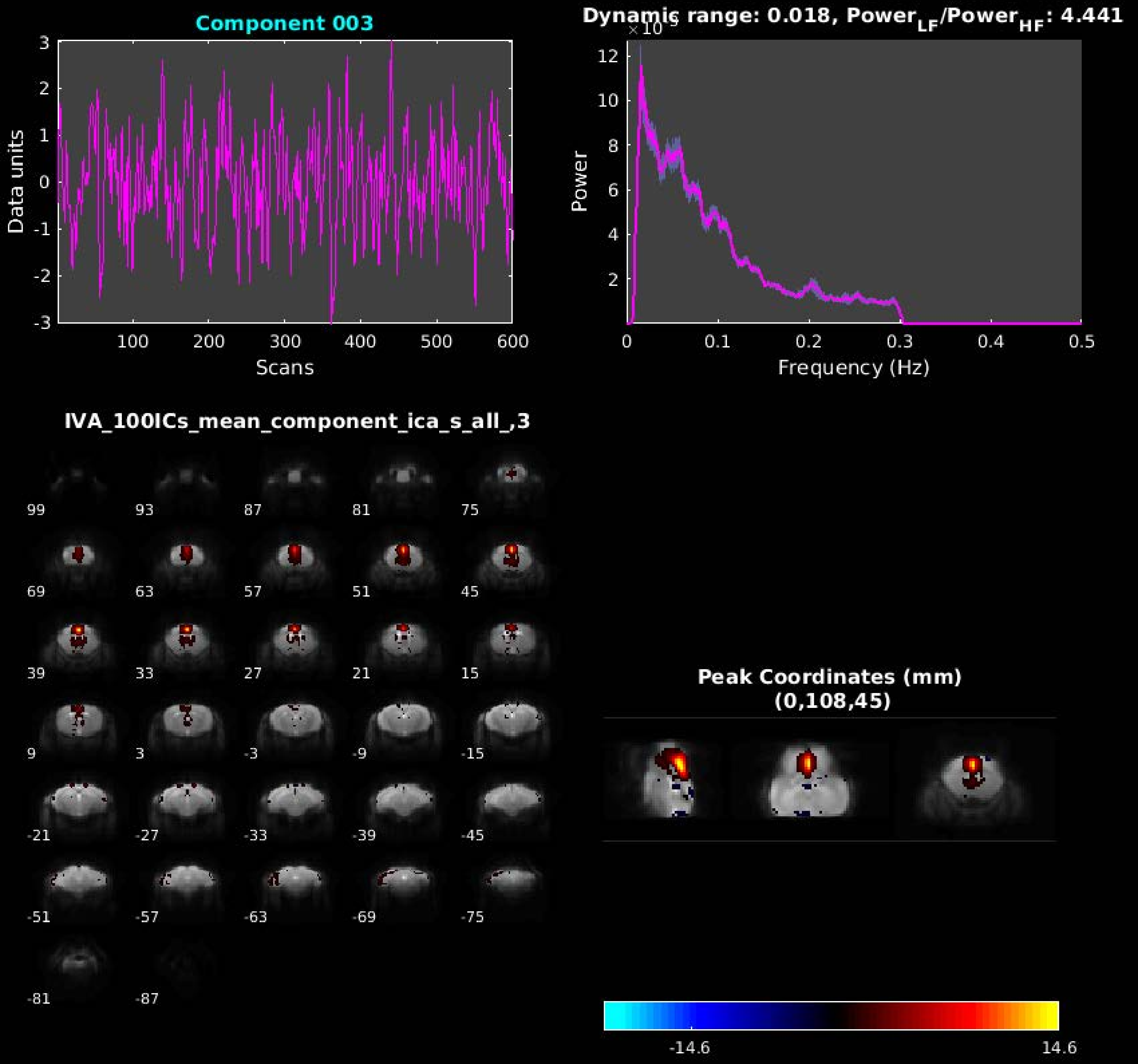

Component 003
Data units
Scans
Dynamic range: 0.018, Power_LF/Power_HF: 4.441
Power
Frequency (Hz)
IVA_100ICs_mean_component_ica_s_all_,3
Peak Coordinates (mm)
(0,108,45)
-14.6
14.6

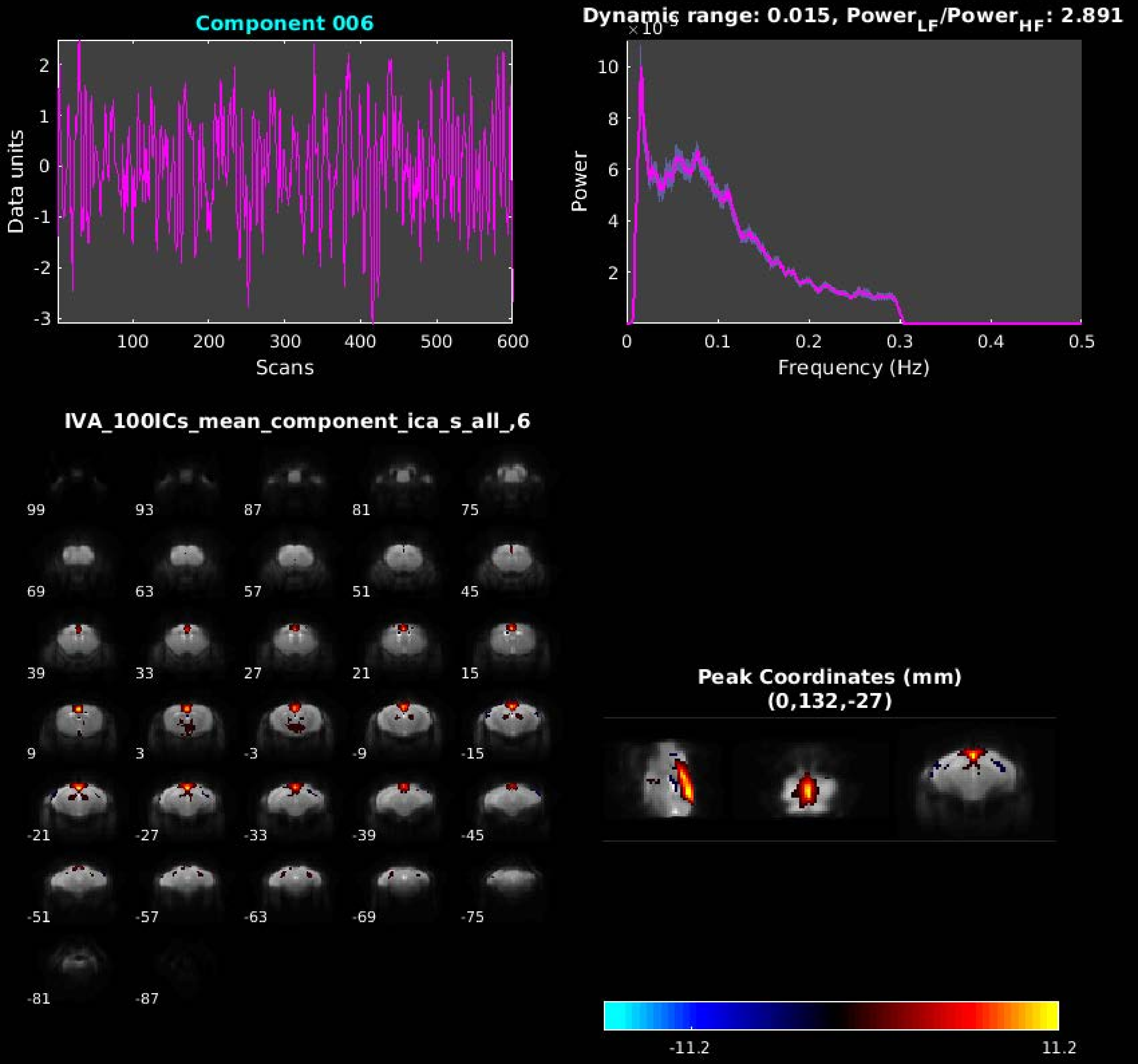
Component 006
Data units
Scans
Dynamic range: 0.015, Power_LF/Power_HF: 2.891
Power
Frequency (Hz)
IVA_100ICs_mean_component_ica_s_all_,6
99
93
87
81
75
69
63
57
51
45
39
33
27
21
15
9
3
-3
-9
-15
-21
-27
-33
-39
-45
-51
-57
-63
-69
-75
-81
-87
Peak Coordinates (mm)
(0,132,-27)
-11.2
11.2

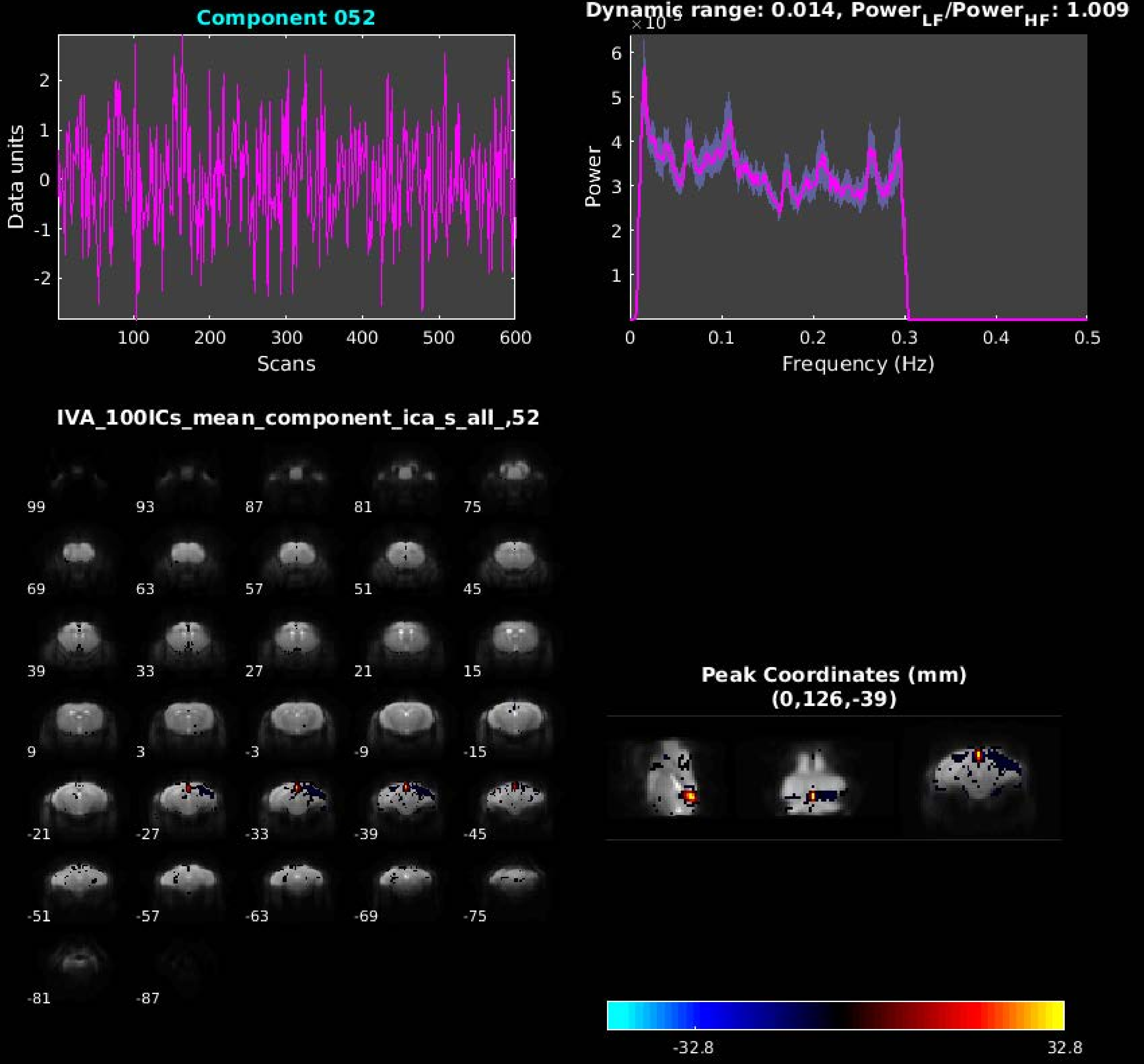

Component 052
Data units
Scans
Dynamic range: 0.014, Power$_{LF}$/Power$_{HF}$: 1.009
$\times 10^{-3}$
Power
Frequency (Hz)
IVA_100ICs_mean_component_ica_s_all_,52
99
93
87
81
75
69
63
57
51
45
39
33
27
21
15
9
3
-3
-9
-15
-21
-27
-33
-39
-45
-51
-57
-63
-69
-75
-81
-87
Peak Coordinates (mm)
(0,126,-39)
-32.8
32.8

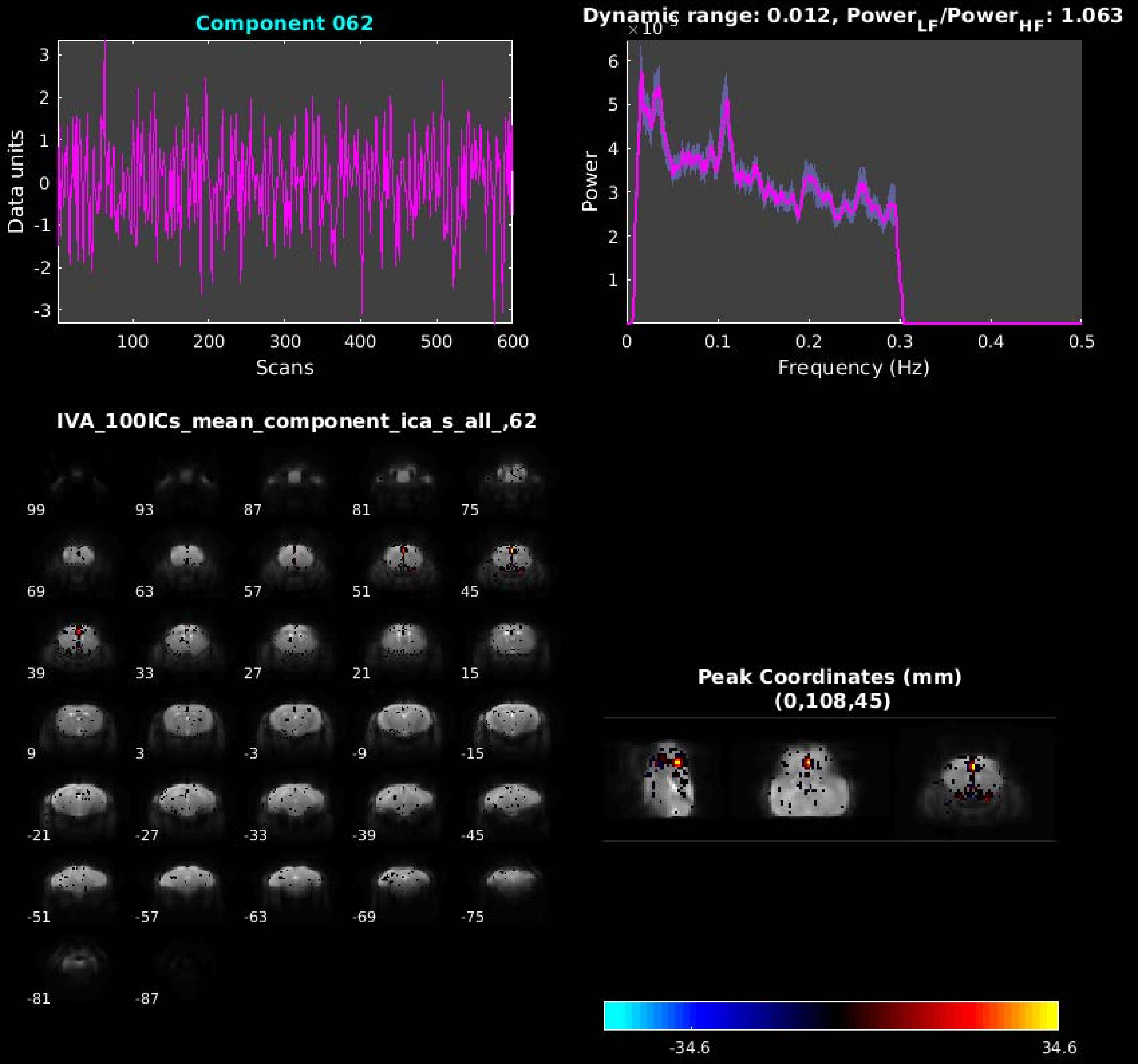
Component 062
Data units
Scans
100
200
300
400
500
600
Dynamic range: 0.012, $Power_{LF}/Power_{HF}$: 1.063
Power
Frequency (Hz)
0
0.1
0.2
0.3
0.4
0.5
IVA_100ICs_mean_component_ica_s_all_,62
99
93
87
81
75
69
63
57
51
45
39
33
27
21
15
9
3
-3
-9
-15
-21
-27
-33
-39
-45
-51
-57
-63
-69
-75
-81
-87
Peak Coordinates (mm)
(0,108,45)
-34.6
34.6

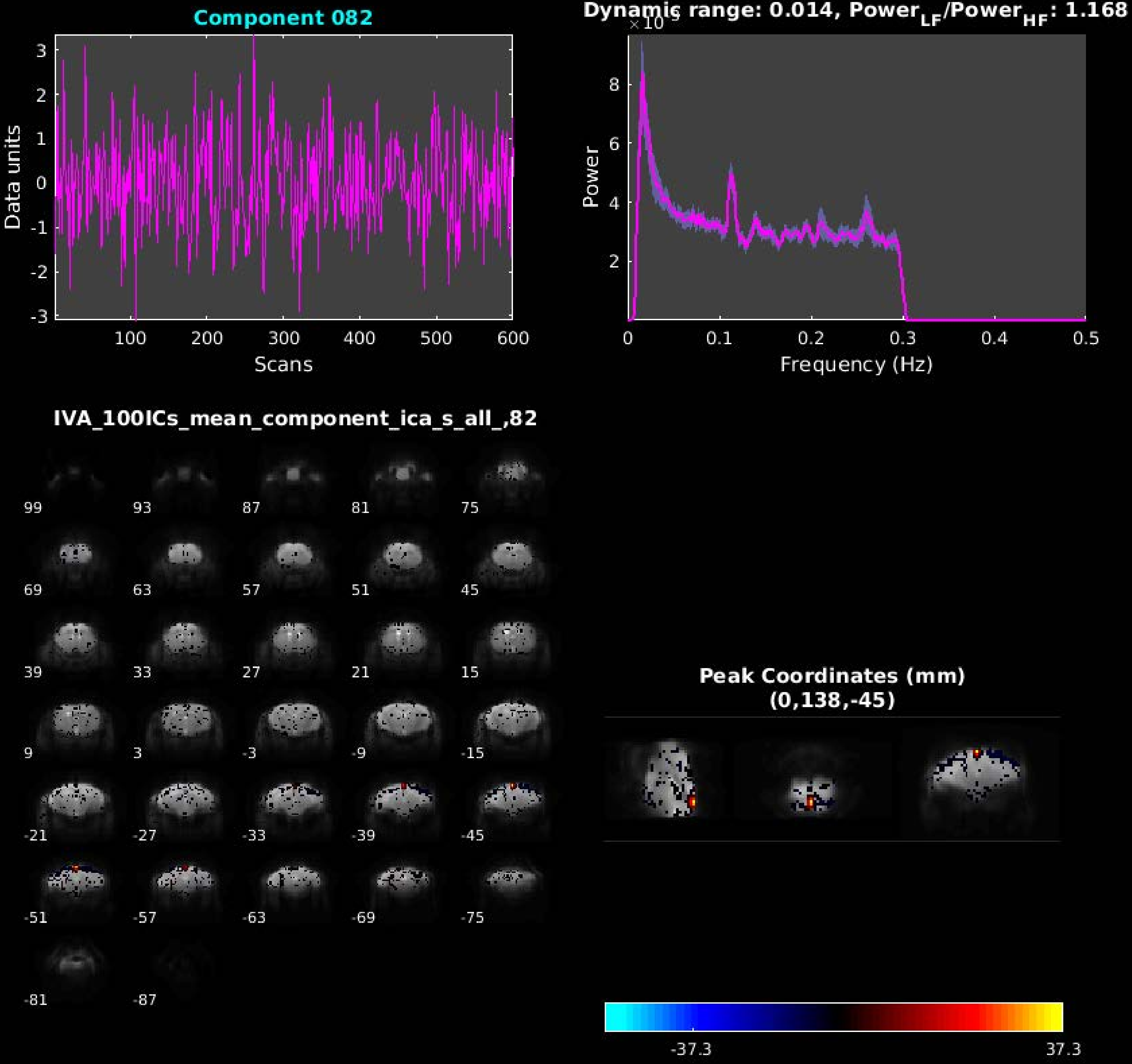

Component 082
Data units
Scans
Dynamic range: 0.014, Power_LF/Power_HF: 1.168
Power
Frequency (Hz)
IVA_100ICs_mean_component_ica_s_all_,82
Peak Coordinates (mm)
(0,138,-45)
-37.3
37.3

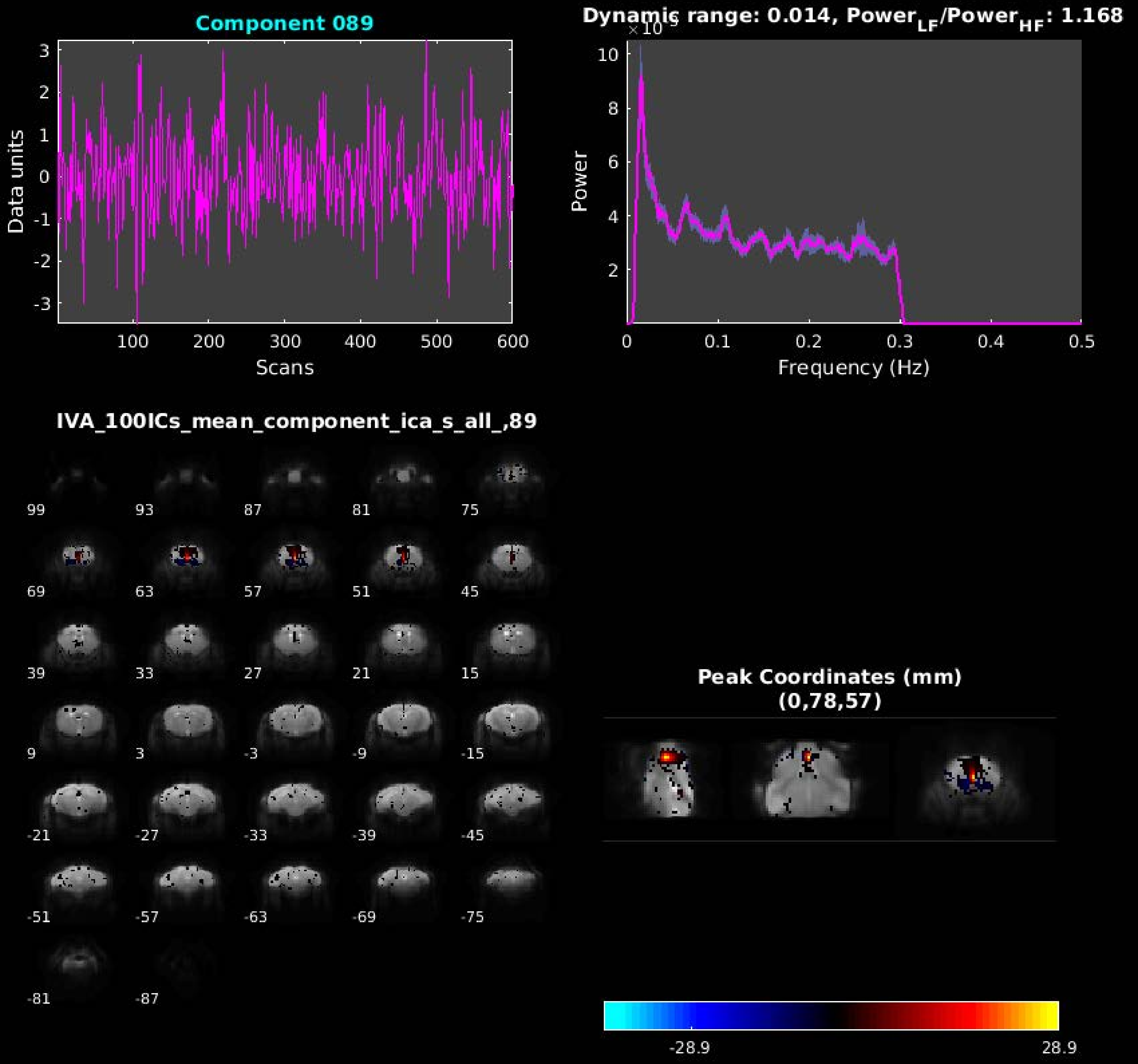

Component 089
Data units
Scans
Dynamic range: 0.014, $Power_{LF}/Power_{HF}$: 1.168
Power
Frequency (Hz)
IVA_100ICs_mean_component_ica_s_all_,89
Peak Coordinates (mm)
(0,78,57)
-28.9
28.9

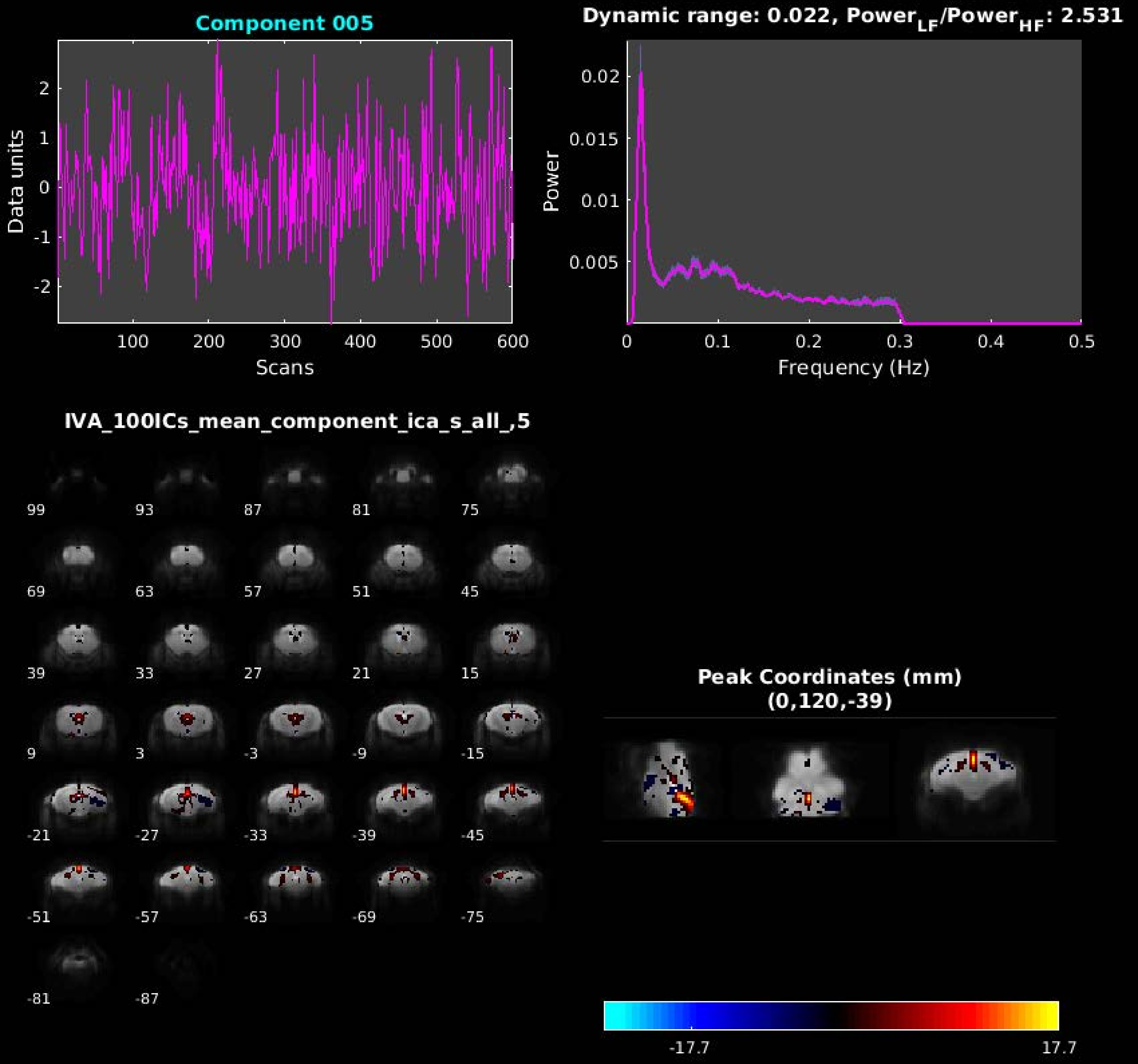
Component 005
Data units
Scans
Dynamic range: 0.022, Power$_{LF}$/Power$_{HF}$: 2.531
Power
Frequency (Hz)
IVA_100ICs_mean_component_ica_s_all_,5
99
93
87
81
75
69
63
57
51
45
39
33
27
21
15
9
3
-3
-9
-15
-21
-27
-33
-39
-45
-51
-57
-63
-69
-75
-81
-87
Peak Coordinates (mm)
(0,120,-39)
-17.7
17.7

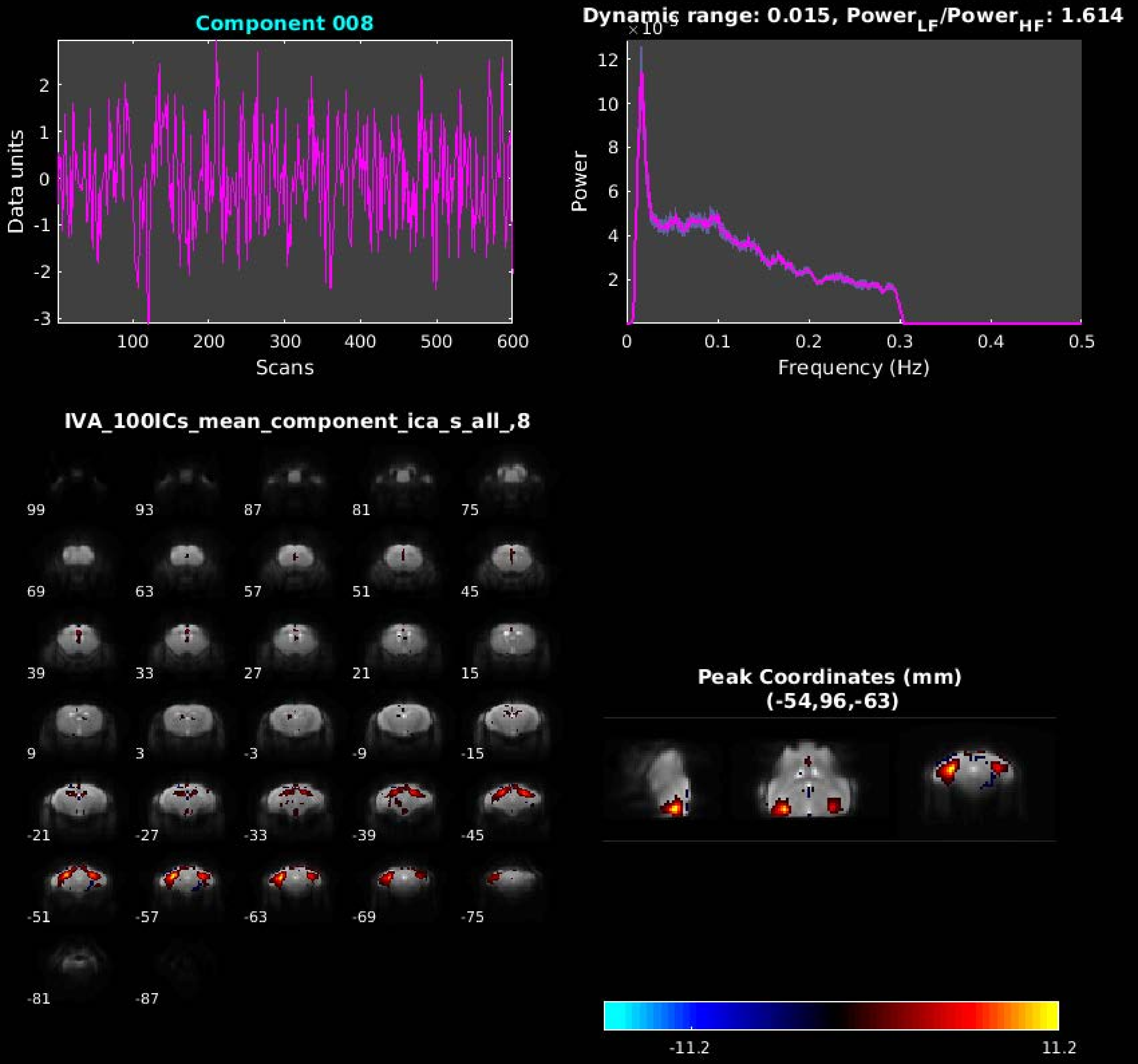

Component 008
Data units
Scans
100
200
300
400
500
600
Dynamic range: 0.015, Power$_{LF}$/Power$_{HF}$: 1.614
$\times 10^{-5}$
Power
Frequency (Hz)
0
0.1
0.2
0.3
0.4
0.5
IVA_100ICs_mean_component_ica_s_all_,8
99
93
87
81
75
69
63
57
51
45
39
33
27
21
15
9
3
-3
-9
-15
-21
-27
-33
-39
-45
-51
-57
-63
-69
-75
-81
-87
Peak Coordinates (mm)
(-54,96,-63)
-11.2
11.2

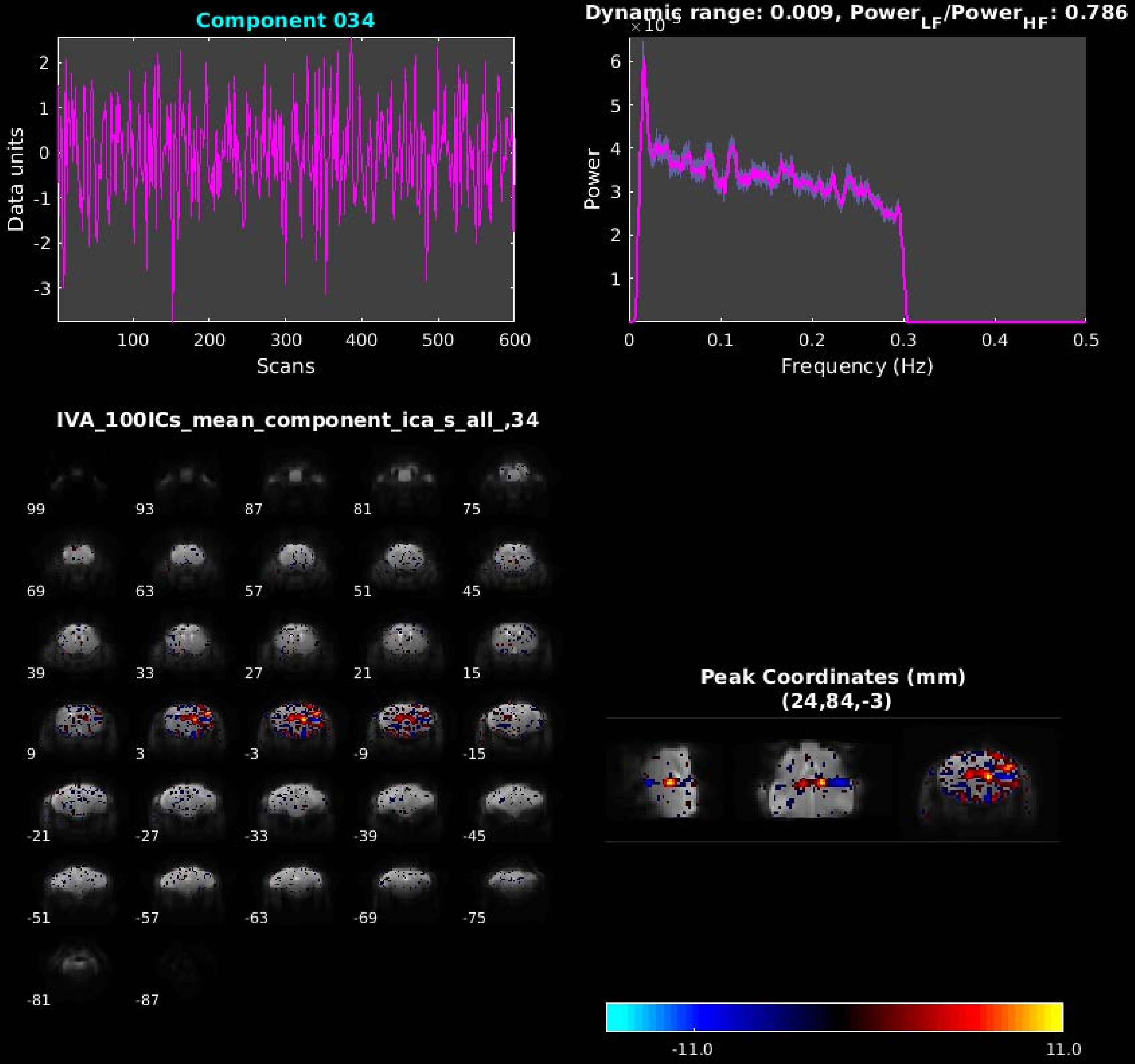

Component 034
Data units
Scans
Dynamic range: 0.009, Power_LF/Power_HF: 0.786
× 10^-5
Power
Frequency (Hz)
IVA_100ICs_mean_component_ica_s_all_,34
99
93
87
81
75
69
63
57
51
45
39
33
27
21
15
9
3
-3
-9
-15
-21
-27
-33
-39
-45
-51
-57
-63
-69
-75
-81
-87
Peak Coordinates (mm)
(24,84,-3)
-11.0
11.0

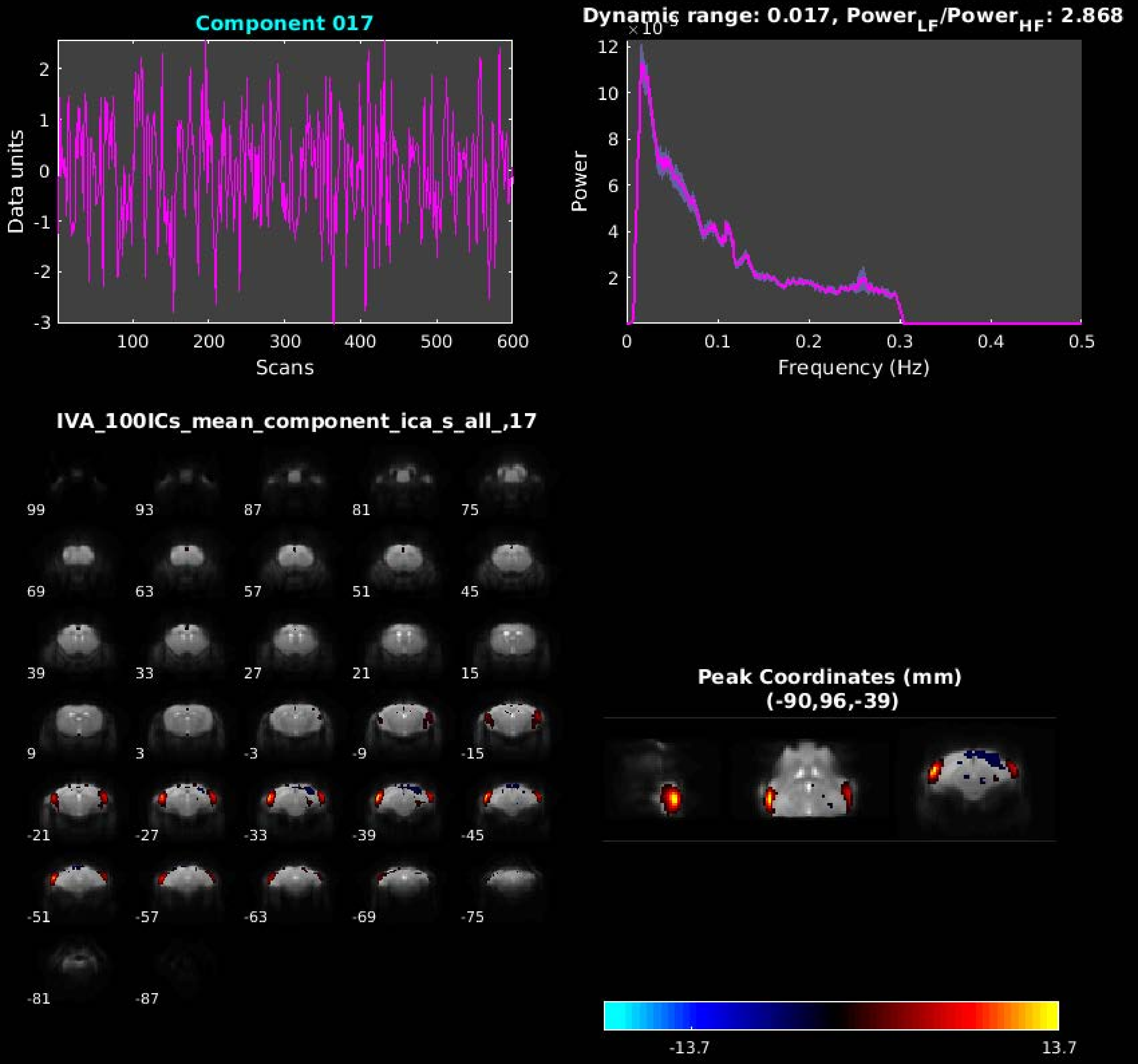

Component 017
Data units
Scans
Dynamic range: 0.017, Power_LF/Power_HF: 2.868
Power
Frequency (Hz)
IVA_100ICs_mean_component_ica_s_all_,17
Peak Coordinates (mm)
(-90,96,-39)
-13.7
13.7

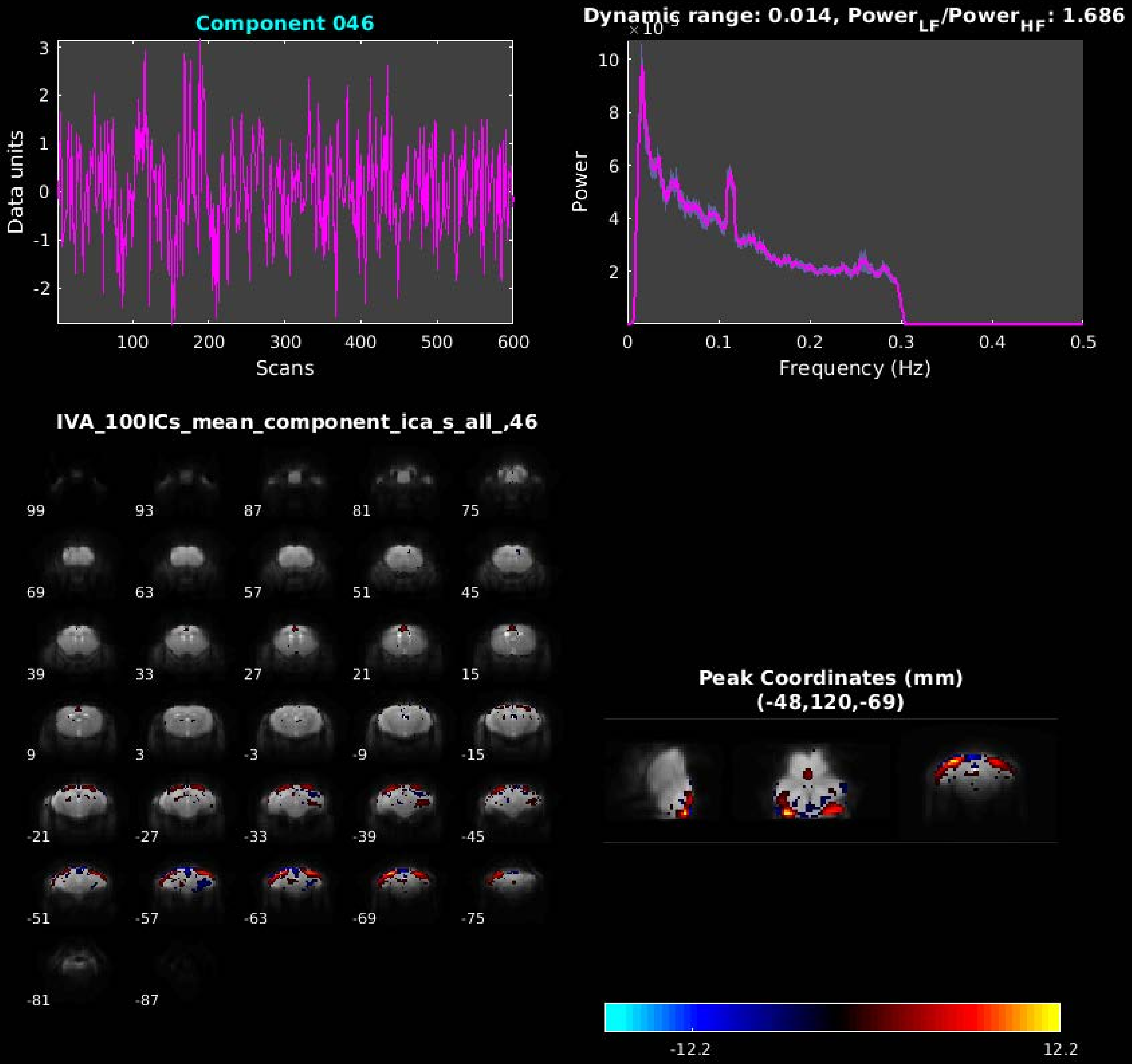

Component 046
Data units
Scans
Dynamic range: 0.014, Power$_{LF}$/Power$_{HF}$: 1.686
Power
Frequency (Hz)
IVA_100ICs_mean_component_ica_s_all_,46
99
93
87
81
75
69
63
57
51
45
39
33
27
21
15
9
3
-3
-9
-15
-21
-27
-33
-39
-45
-51
-57
-63
-69
-75
-81
-87
Peak Coordinates (mm)
(-48,120,-69)
-12.2
12.2

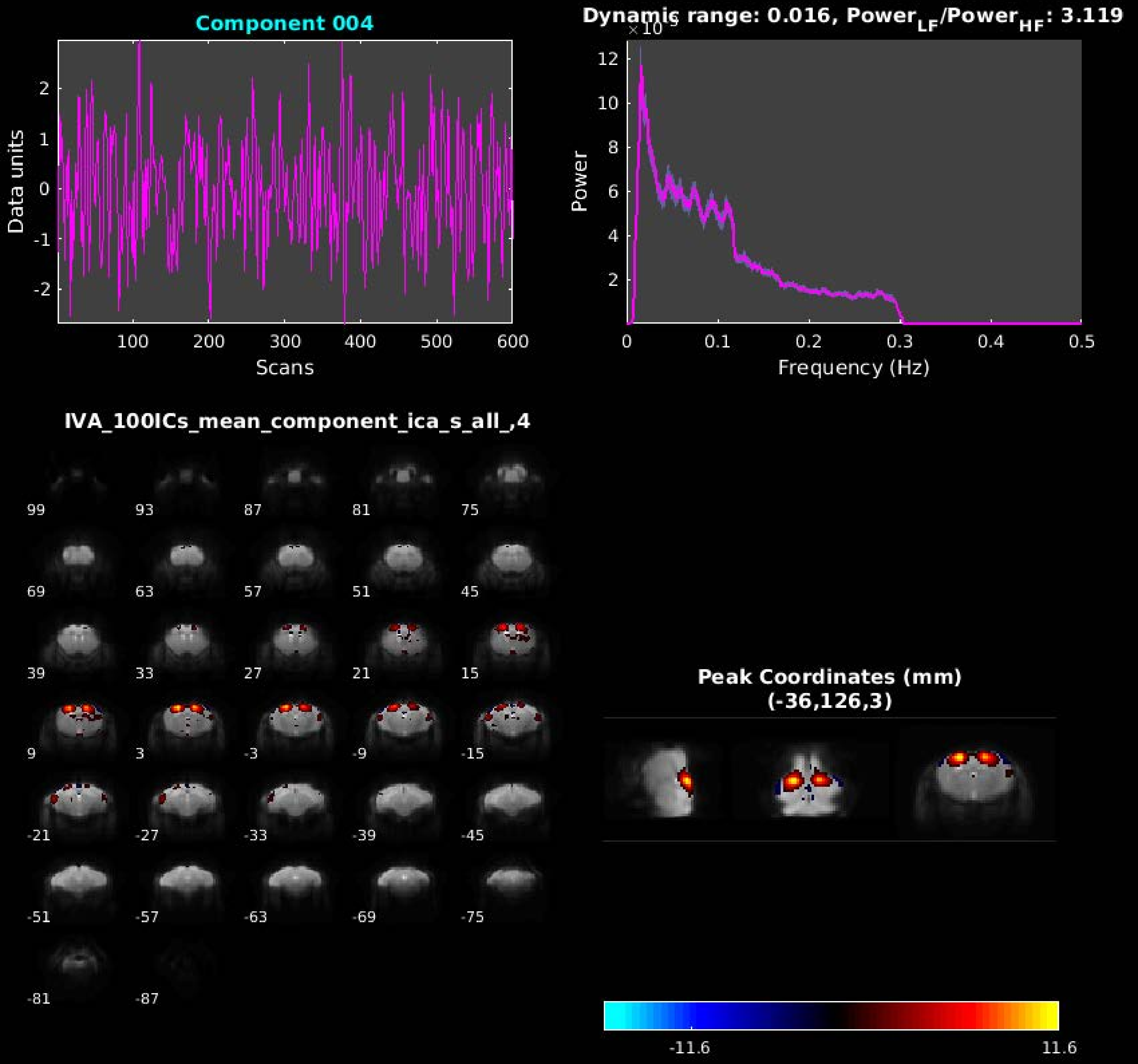

Component 004
Data units
Scans
Dynamic range: 0.016, Power_LF/Power_HF: 3.119
×10
Power
Frequency (Hz)
IVA_100ICs_mean_component_ica_s_all_,4
99
93
87
81
75
69
63
57
51
45
39
33
27
21
15
9
3
-3
-9
-15
-21
-27
-33
-39
-45
-51
-57
-63
-69
-75
-81
-87
Peak Coordinates (mm)
(-36,126,3)
-11.6
11.6

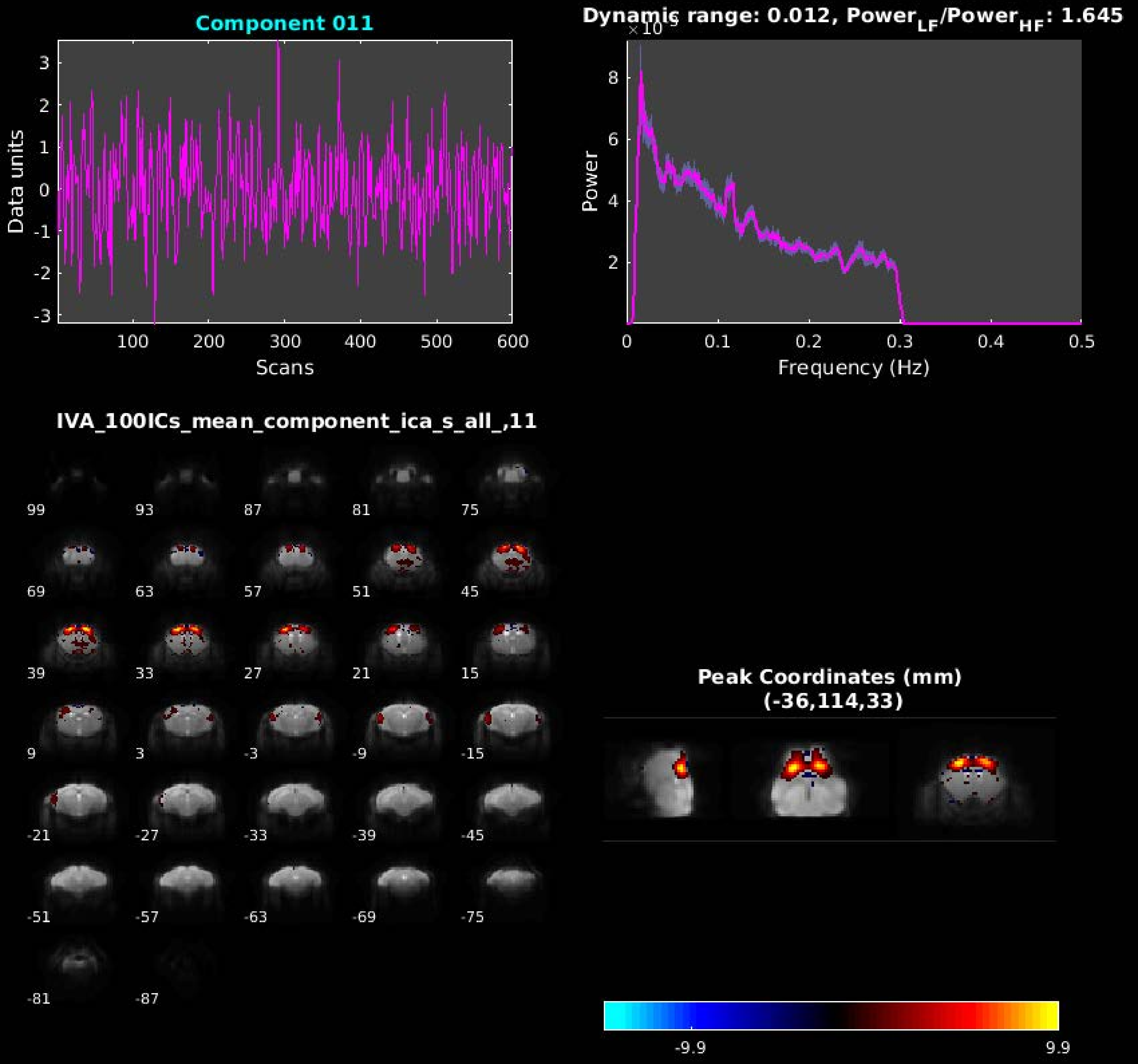
Component 011
Data units
Scans
Dynamic range: 0.012, Power$_{LF}$/Power$_{HF}$: 1.645
Power
Frequency (Hz)
IVA_100ICs_mean_component_ica_s_all_,11
Peak Coordinates (mm)
(-36,114,33)
-9.9
9.9

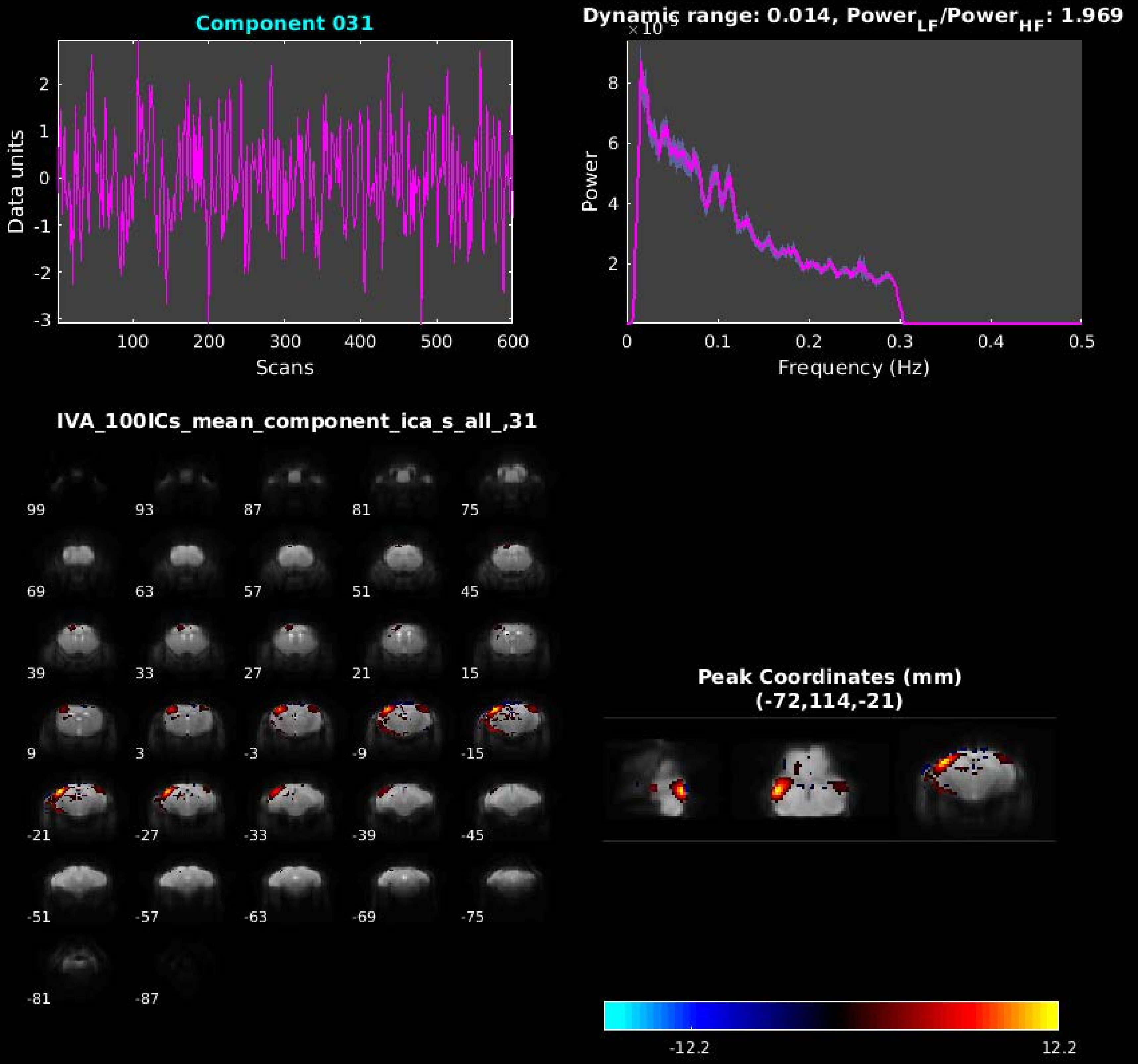
Component 031
Data units
Scans
Dynamic range: 0.014, Power_LF/Power_HF: 1.969
×10^-5
Power
Frequency (Hz)
IVA_100ICs_mean_component_ica_s_all_,31
99 93 87 81 75
69 63 57 51 45
39 33 27 21 15
9 3 -3 -9 -15
-21 -27 -33 -39 -45
-51 -57 -63 -69 -75
-81 -87
Peak Coordinates (mm)
(-72,114,-21)
-12.2
12.2

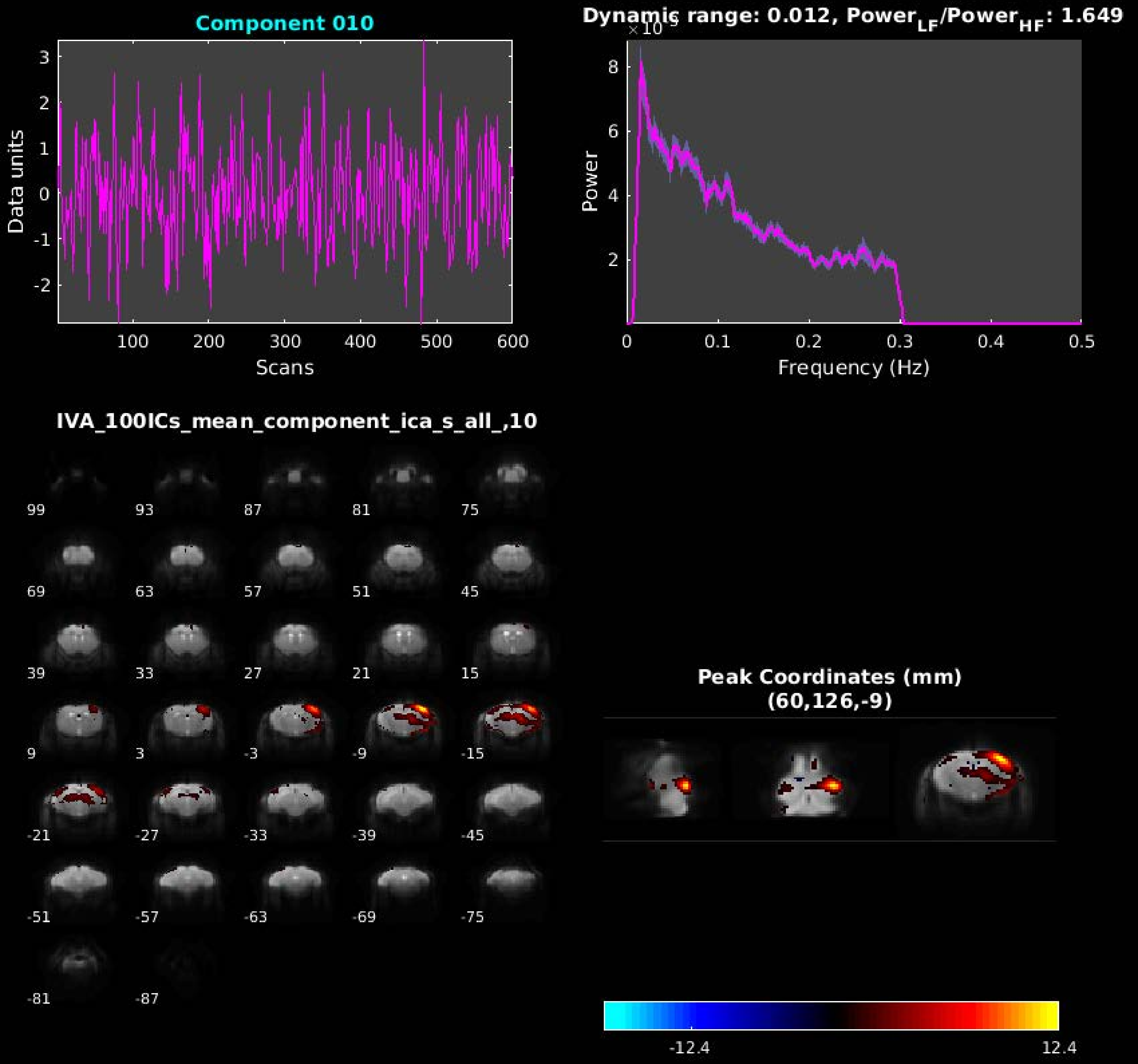

Component 010
Data units
3
2
1
0
-1
-2
100
200
300
400
500
600
Scans
Dynamic range: 0.012, Power_LF/Power_HF: 1.649
× 10
8
6
4
2
Power
0
0.1
0.2
0.3
0.4
0.5
Frequency (Hz)
IVA_100ICs_mean_component_ica_s_all_,10
99
93
87
81
75
69
63
57
51
45
39
33
27
21
15
9
3
-3
-9
-15
-21
-27
-33
-39
-45
-51
-57
-63
-69
-75
-81
-87
Peak Coordinates (mm)
(60,126,-9)
-12.4
12.4

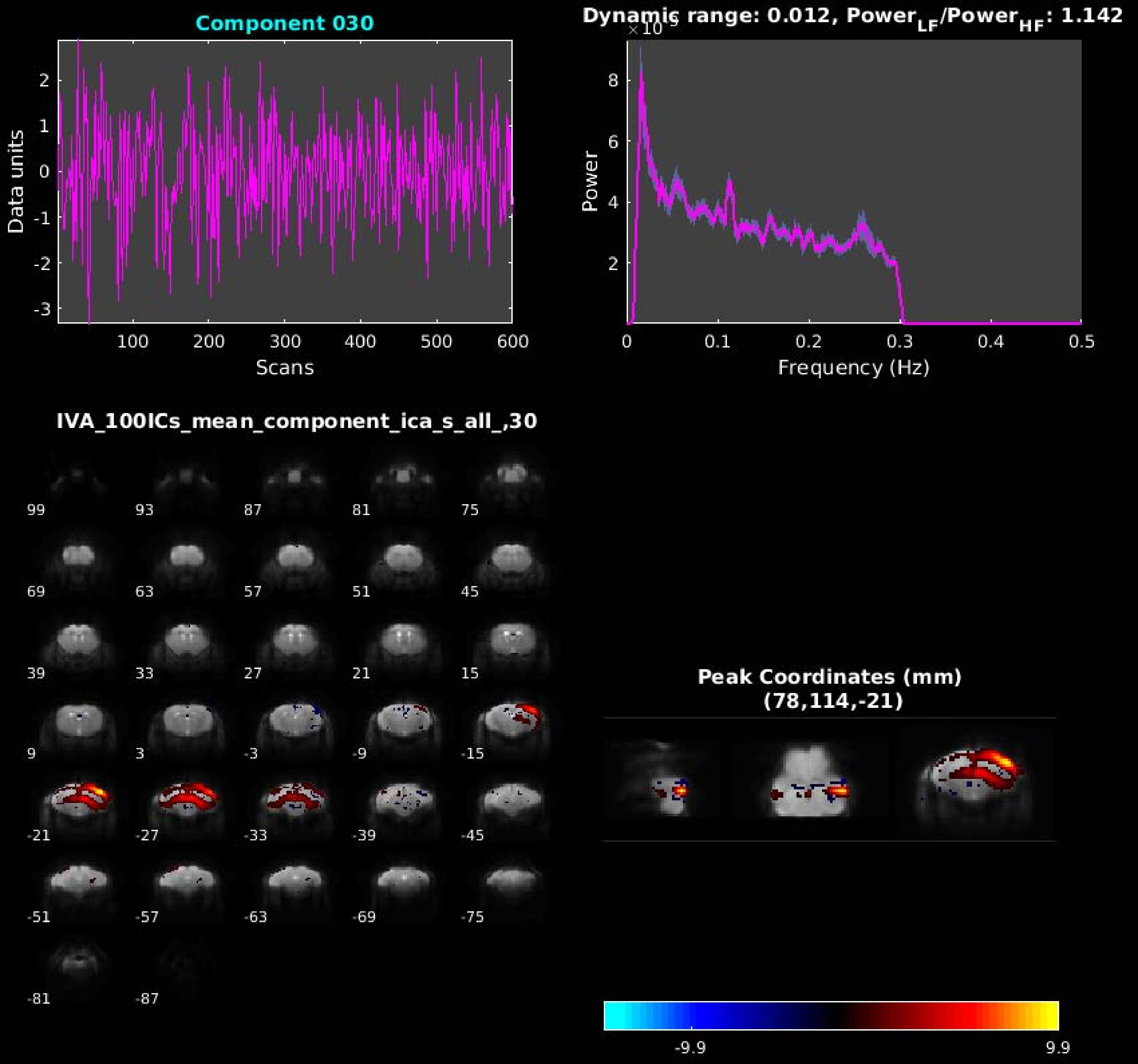
Component 030
Data units
Scans
Dynamic range: 0.012, Power_LF/Power_HF: 1.142
Power
Frequency (Hz)
IVA_100ICs_mean_component_ica_s_all_,30
99
93
87
81
75
69
63
57
51
45
39
33
27
21
15
9
3
-3
-9
-15
-21
-27
-33
-39
-45
-51
-57
-63
-69
-75
-81
-87
Peak Coordinates (mm)
(78,114,-21)
-9.9
9.9

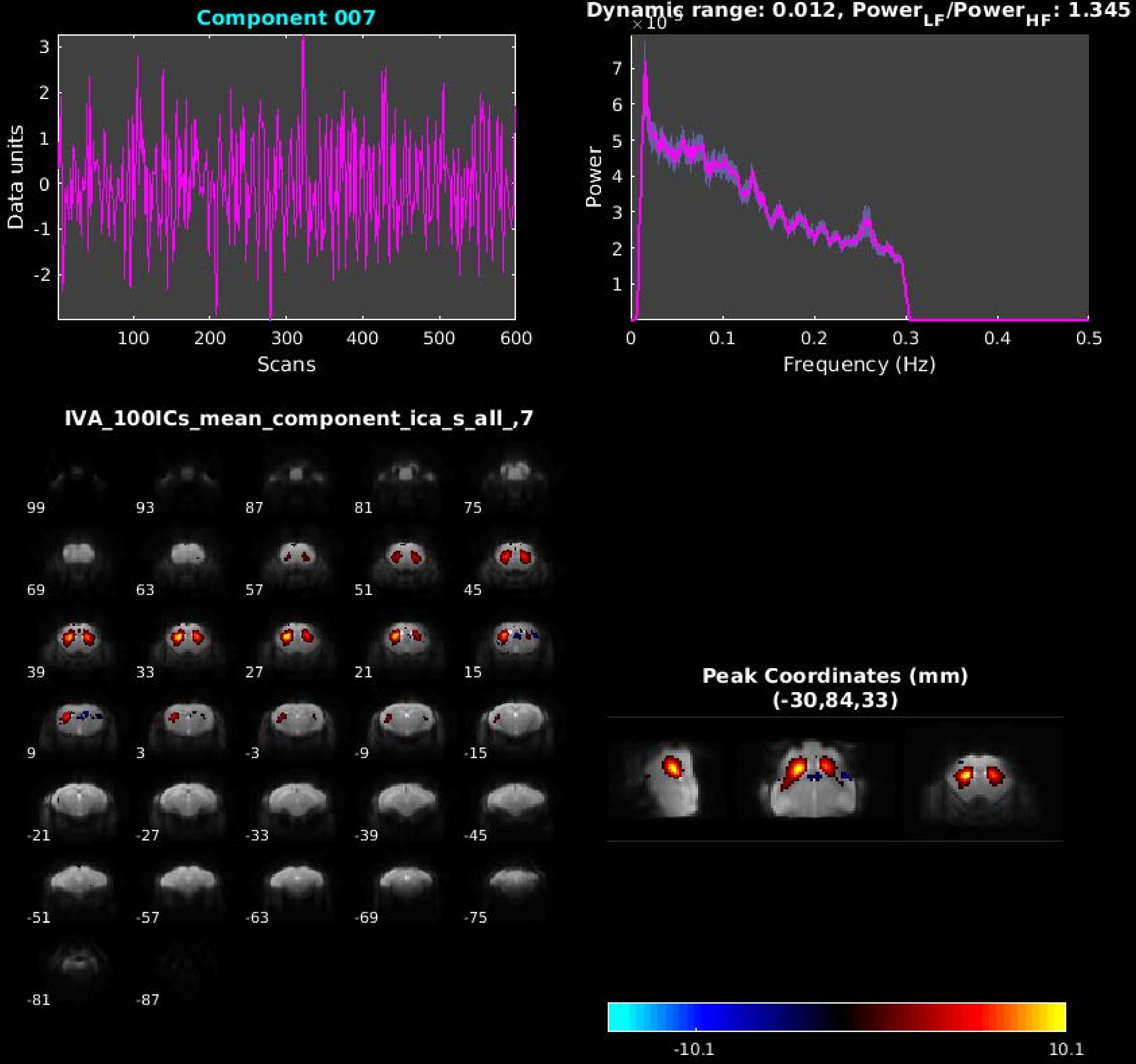
Component 007
Data units
Scans
Dynamic range: 0.012, Power$_{LF}$/Power$_{HF}$: 1.345
Power
Frequency (Hz)
IVA_100ICs_mean_component_ica_s_all_,7
Peak Coordinates (mm)
(-30,84,33)
-10.1
10.1

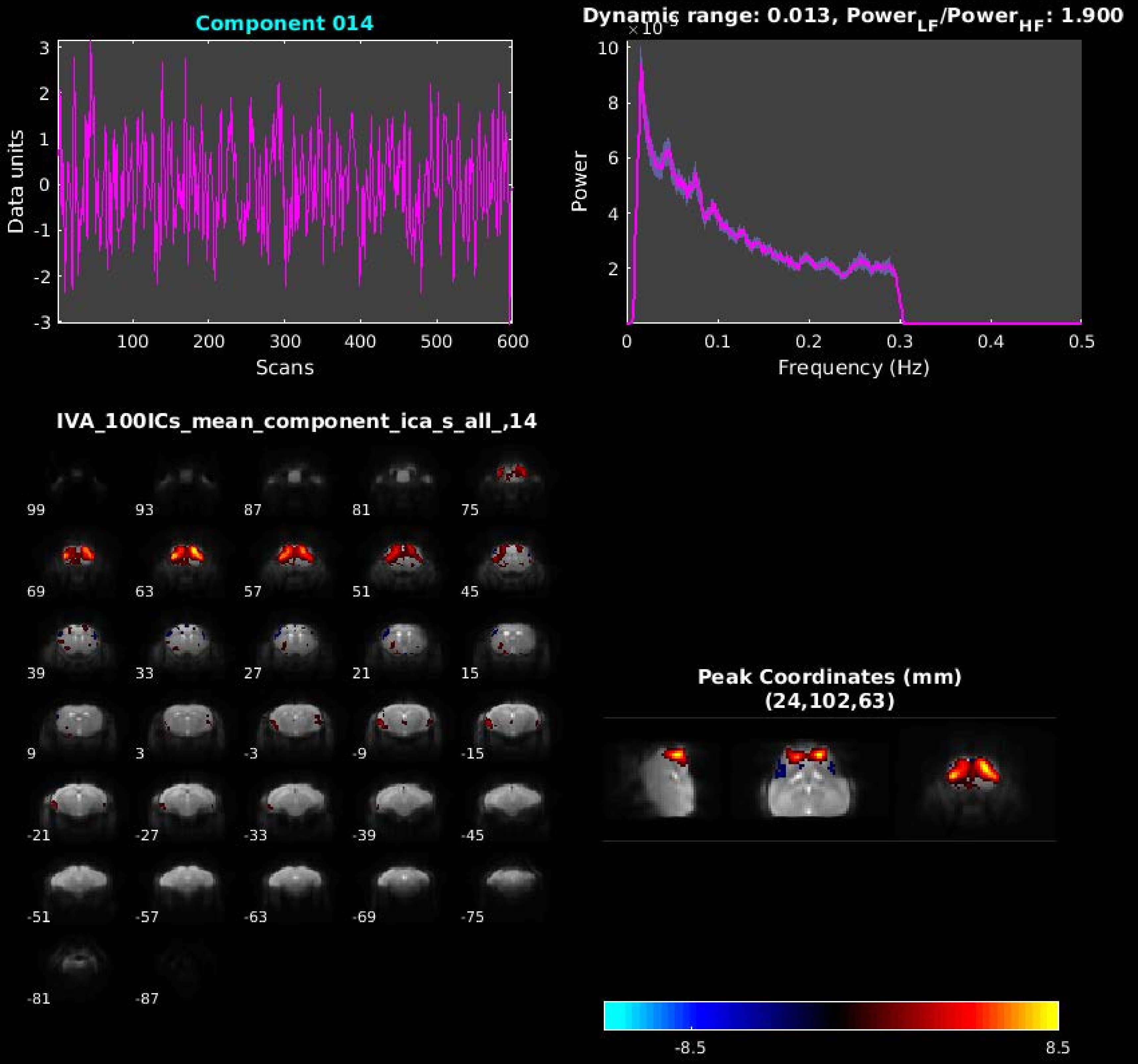

Component 014
Data units
Scans
Dynamic range: 0.013, Power$_{LF}$/Power$_{HF}$: 1.900
$\times 10^{-3}$
Power
Frequency (Hz)
IVA_100ICs_mean_component_ica_s_all_,14
99 93 87 81 75
69 63 57 51 45
39 33 27 21 15
9 3 -3 -9 -15
-21 -27 -33 -39 -45
-51 -57 -63 -69 -75
-81 -87
Peak Coordinates (mm)
(24,102,63)
-8.5
8.5

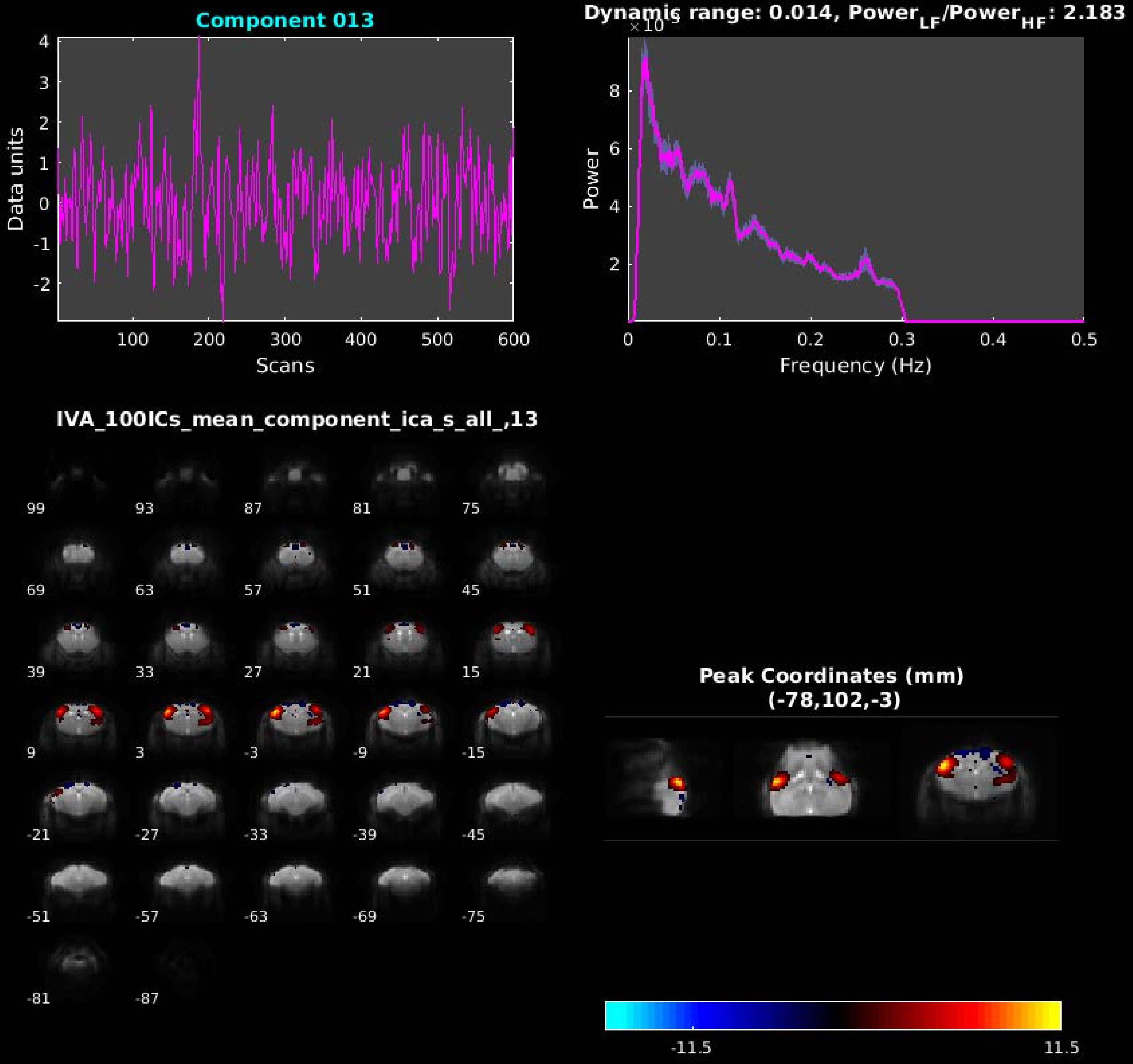
Component 013
Data units
Scans
Dynamic range: 0.014, Power$_{LF}$/Power$_{HF}$: 2.183
Power
Frequency (Hz)
IVA_100ICs_mean_component_ica_s_all_,13
99 93 87 81 75
69 63 57 51 45
39 33 27 21 15
9 3 -3 -9 -15
-21 -27 -33 -39 -45
-51 -57 -63 -69 -75
-81 -87
Peak Coordinates (mm)
(-78,102,-3)
-11.5
11.5

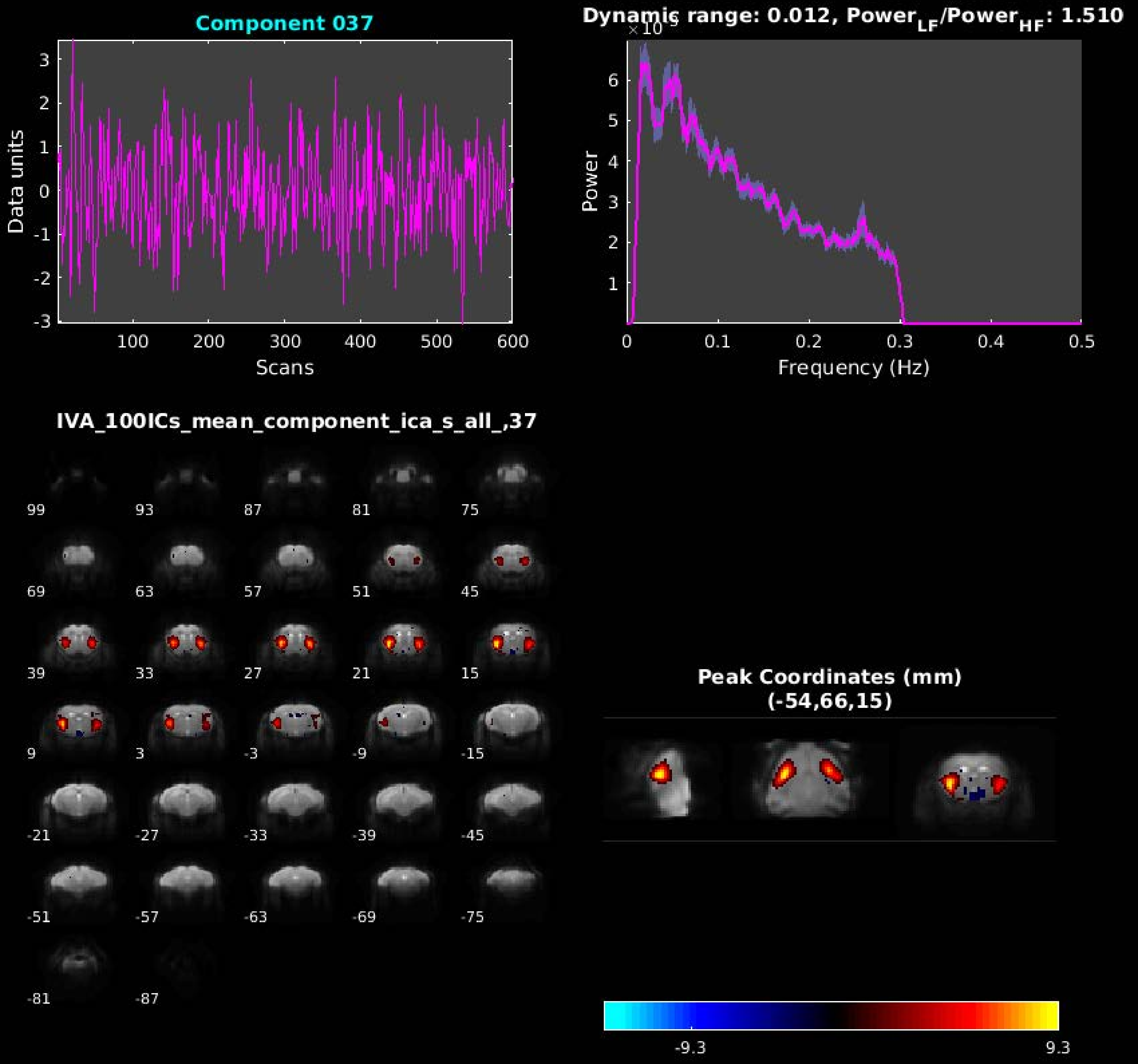
Component 037
Data units
Scans
Dynamic range: 0.012, Power$_{LF}$/Power$_{HF}$: 1.510
×10^{-5}
Power
Frequency (Hz)
IVA_100ICs_mean_component_ica_s_all_,37
Peak Coordinates (mm)
(-54,66,15)
-9.3
9.3

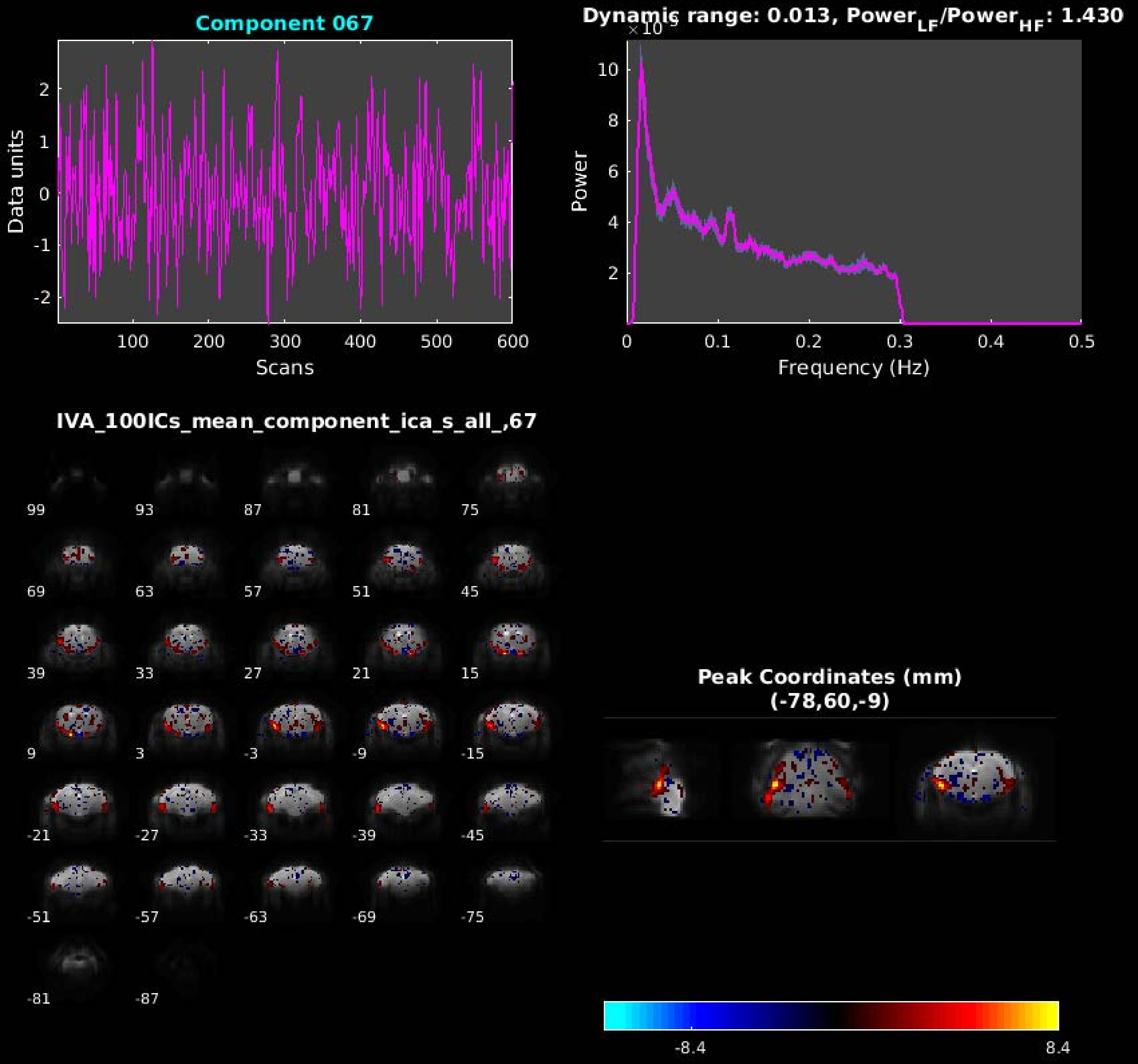

Component 067
Data units
Scans
Dynamic range: 0.013, Power_LF/Power_HF: 1.430
Power
Frequency (Hz)
IVA_100ICs_mean_component_ica_s_all_,67
99 93 87 81 75
69 63 57 51 45
39 33 27 21 15
9 3 -3 -9 -15
-21 -27 -33 -39 -45
-51 -57 -63 -69 -75
-81 -87
Peak Coordinates (mm)
(-78,60,-9)
-8.4
8.4

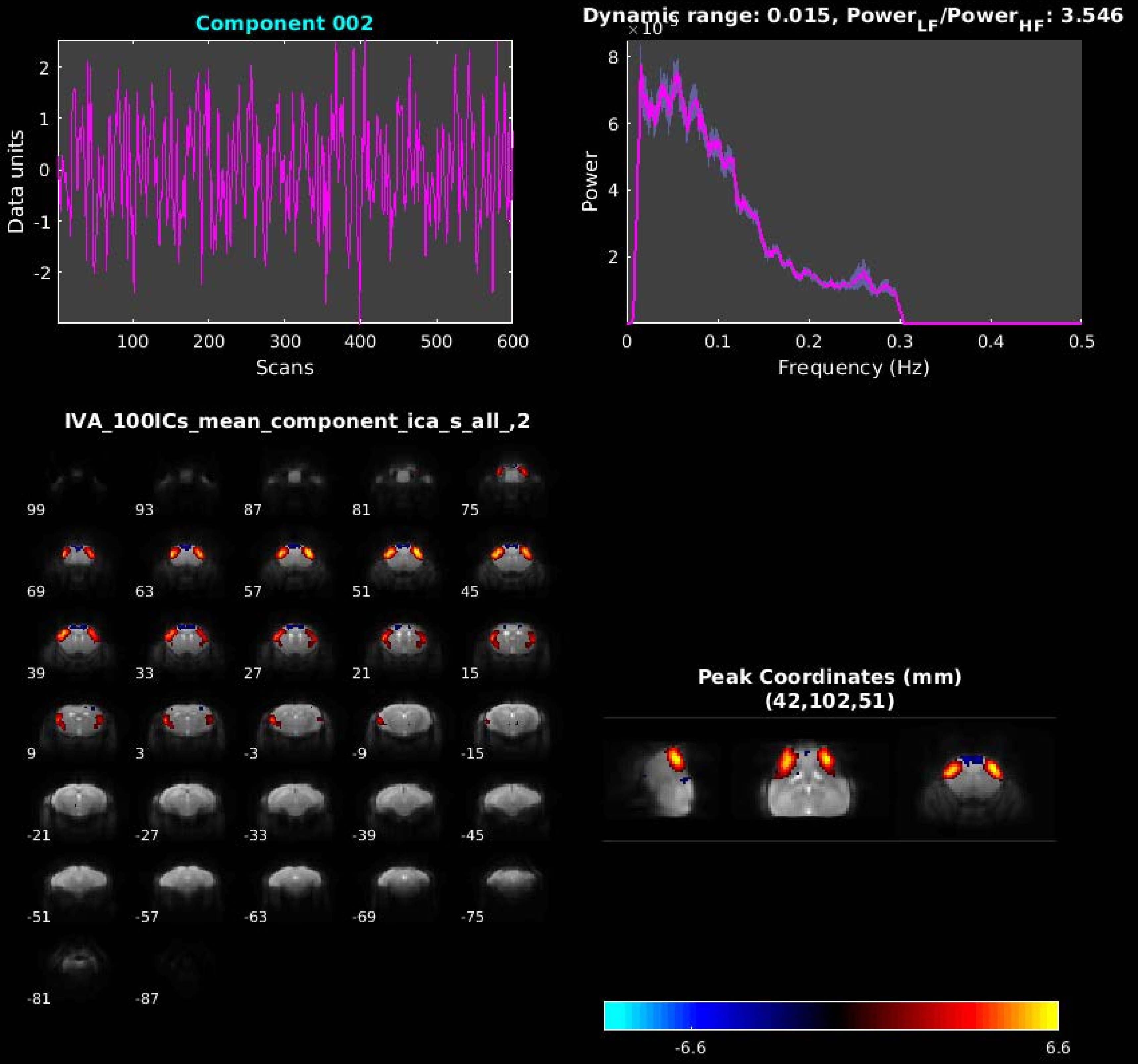
Component 002
Data units
Scans
Dynamic range: 0.015, Power$_{LF}$/Power$_{HF}$: 3.546
$\times 10^{-5}$
Power
Frequency (Hz)
IVA_100ICs_mean_component_ica_s_all_,2
99
93
87
81
75
69
63
57
51
45
39
33
27
21
15
9
3
-3
-9
-15
-21
-27
-33
-39
-45
-51
-57
-63
-69
-75
-81
-87
Peak Coordinates (mm)
(42,102,51)
-6.6
6.6

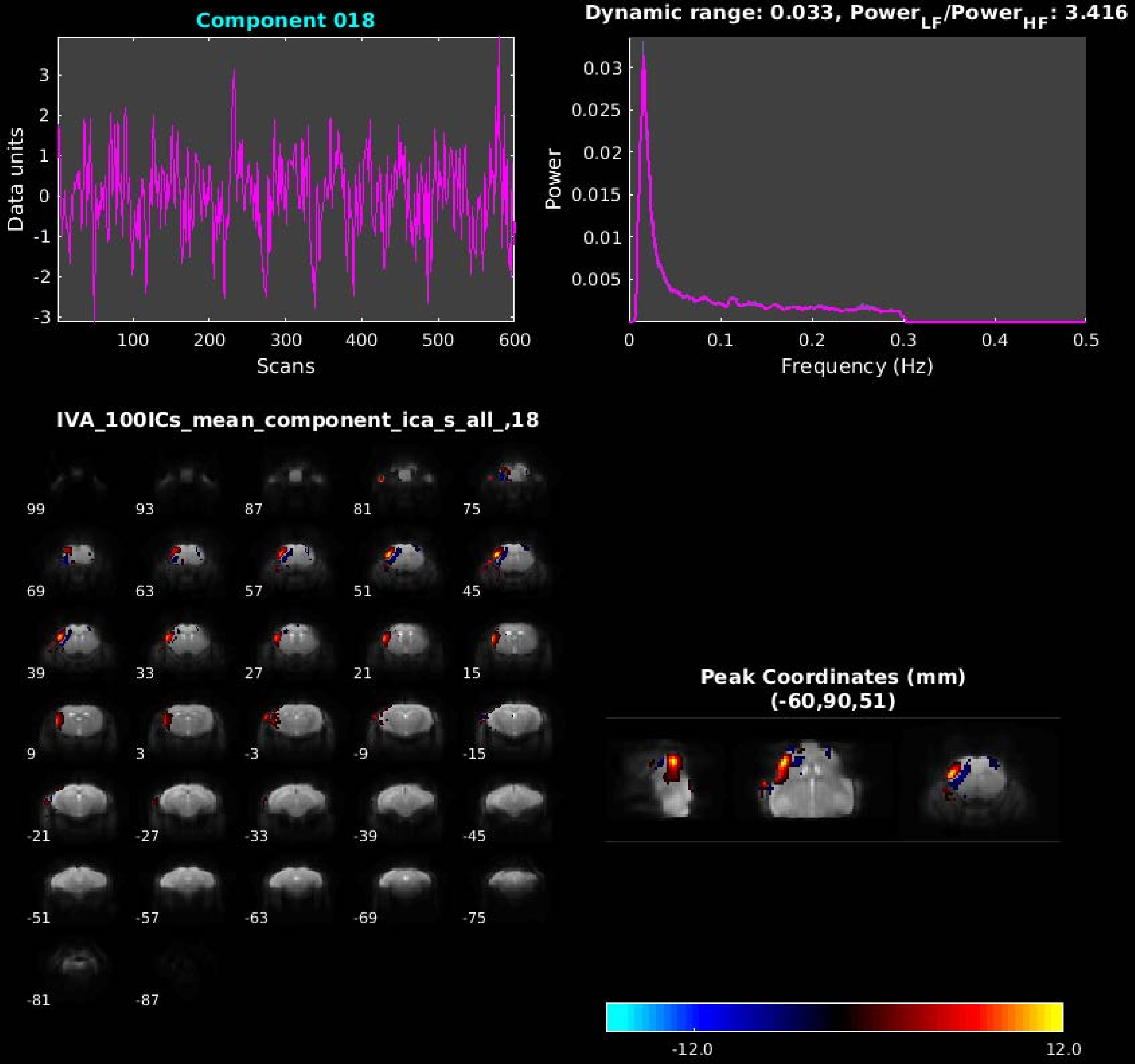

Component 018
Data units
Scans
Dynamic range: 0.033, Power_LF/Power_HF: 3.416
Power
Frequency (Hz)
IVA_100ICs_mean_component_ica_s_all_,18
99
93
87
81
75
69
63
57
51
45
39
33
27
21
15
9
3
-3
-9
-15
-21
-27
-33
-39
-45
-51
-57
-63
-69
-75
-81
-87
Peak Coordinates (mm)
(-60,90,51)
-12.0
12.0

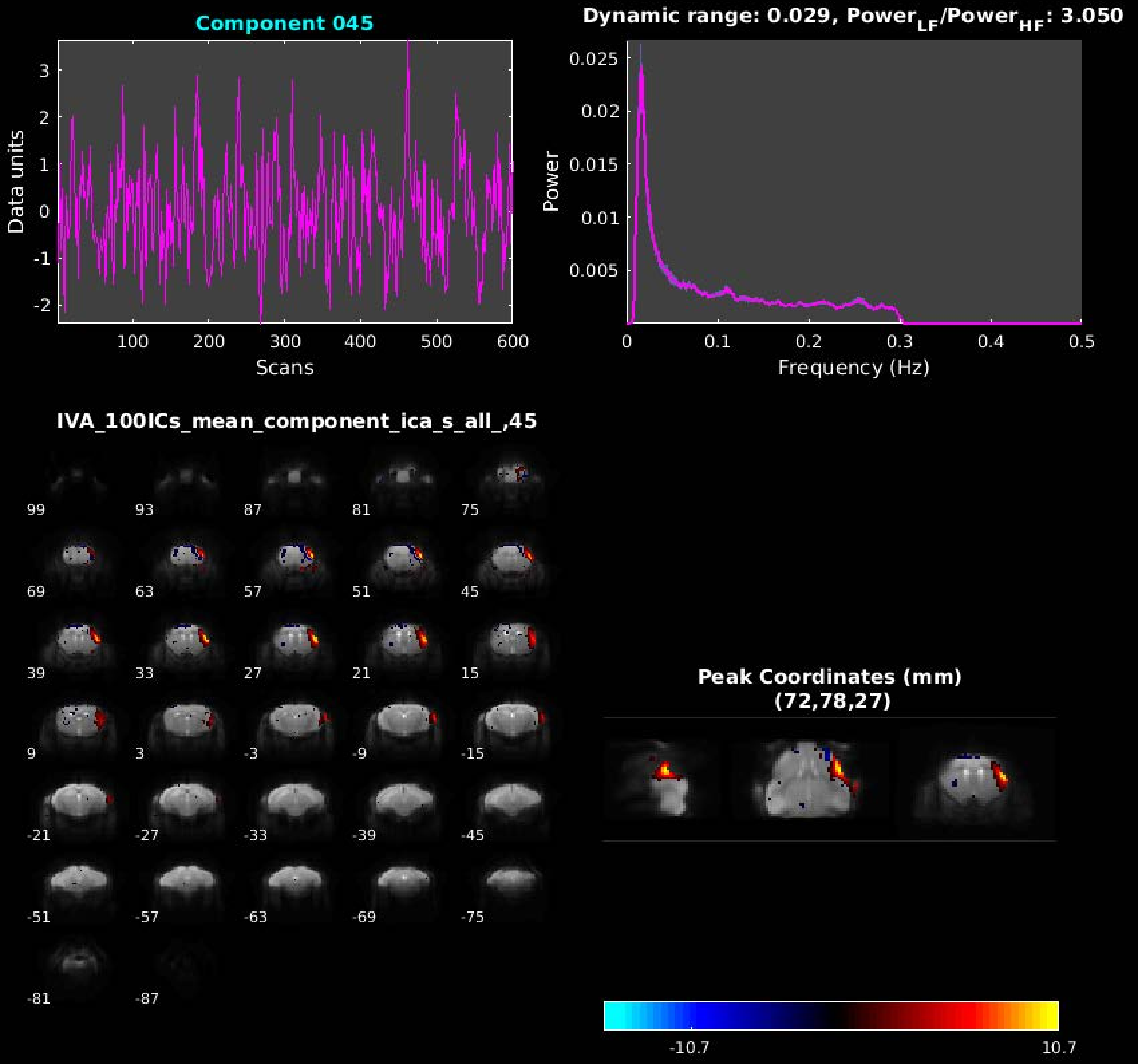

Component 045
Data units
Scans
100
200
300
400
500
600
3
2
1
0
-1
-2
Dynamic range: 0.029, Power_LF/Power_HF: 3.050
Power
Frequency (Hz)
0.025
0.02
0.015
0.01
0.005
0
0.1
0.2
0.3
0.4
0.5
IVA_100ICs_mean_component_ica_s_all_,45
99
93
87
81
75
69
63
57
51
45
39
33
27
21
15
9
3
-3
-9
-15
-21
-27
-33
-39
-45
-51
-57
-63
-69
-75
-81
-87
Peak Coordinates (mm)
(72,78,27)
-10.7
10.7
