# Supplementary figures and images for: A roadmap of brain recovery in a mouse model of concussion: insights from neuroimaging
Source: Acta Neuropathol Commun. 2021 Jan 6;9:2. doi: 10.1186/s40478-020-01098-y (PMC7789702; doi:10.1186/s40478-020-01098-y)

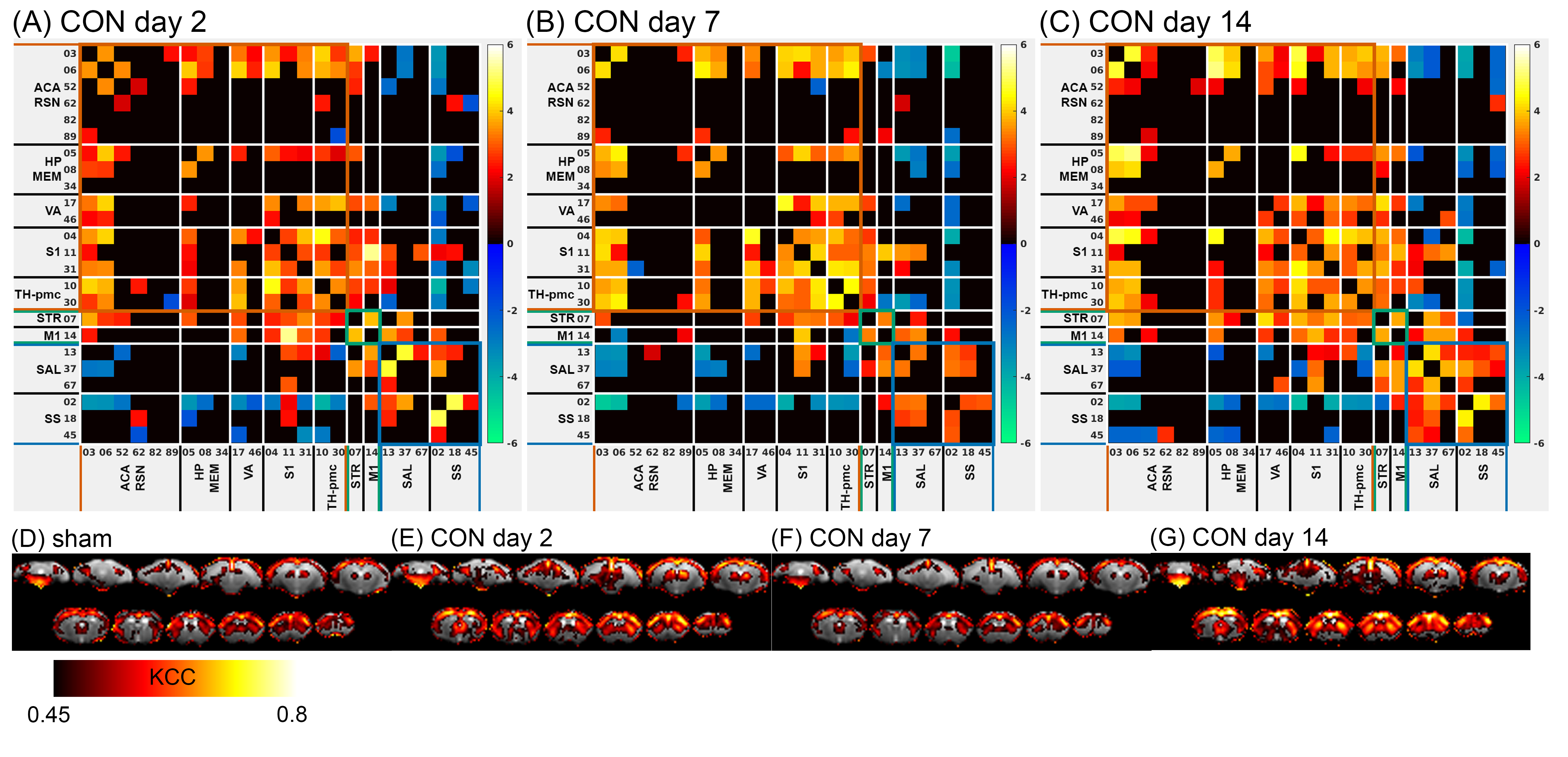

Supplement: Supplementary file 2 — Additional file 2: Figure S2. Group averaged resting-state MRI functional connectivity. (A–D) Average functional network connectivity (FNC) matrices among Independent Components (ICs) identified by IVA-GL of (A) sham (n = 14), (B) CON day 2 (n = 9), (C) CON day 7 (n = 10), and (D) CON day 14 (n = 10) cohorts. Colour scaled by z test statistics; non-black cells were defined as component-component connectivity deemed statistically significant. One sample t-tests, permutation-tested, and FDR-corrected (q-value < 0.05, two-tailed). (E–H) Regional Homogeneity analysis’s local intrinsic functional connectivity represented as averaged seven-voxels-neighbourhood Kendall’s Coefficient of Concordance (KCC) maps of (E) sham, (F) CON day 2, (G) CON day 7, and (H) CON day 14 cohorts. [file 40478_2020_1098_MOESM2_ESM.png]

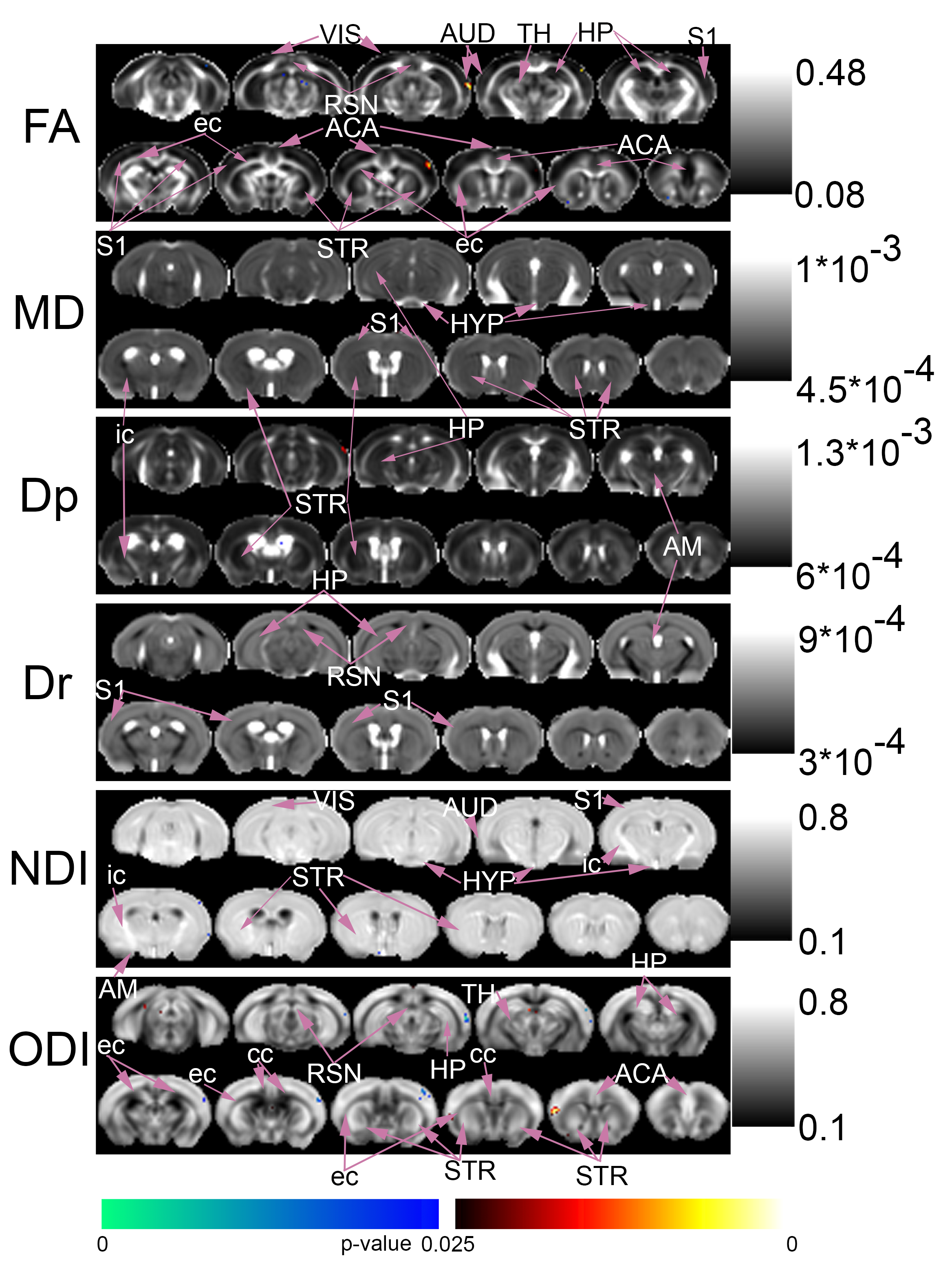

Supplement: Supplementary file 3 — Additional file 3: Figure S3. Randomise reproducibility test of DTI and NODDI metrics. Voxel-by-voxel statistical analysis results of Diffusion Tensor Imaging (FA = Fractional Anisotropy, MD = Mean Diffusivity, Dp = Parallel Diffusivity, Dr = Radial Diffusivity) and Neurite Orientation Dispersion and Density Imaging metrics (NDI = Neurite Density Index, ODI = Orientation Dispersion Index) and Tensor-based Morphometry with Jacobian Index (JI) reproducibility test. Statistical map thresholded at P value < 0.05 (two-tailed), unpaired two sample t-test, implemented as permutation tested for the General Linear Model, corrected for multiple comparisons with mass-based FSL’s Threshold-free Cluster enhancement (TFCE). Statistical maps were overlaid on the averaged and registered DTI and NODDI metrics maps corresponding to the statistical maps (DTI and NODDI results) and structural template (TBM results). Corresponding grey scale map for each averaged DTI and NODDI metrics maps were provided; units for Dp, Dr, and MD were in mm/s2. ACA = Anterior Cingulate Area, AM = Amygdala, AUD = Auditory Area, cc = corpus callosum, ec = external capsule, HP = Hippocampus, HYP = Hypothalamus, ic = internal capsule, INS = Insula, MB = Midbrain, PAL = Palladium, S1 = Primary Somatosensory Cortex, RSN = Retrosplenial Area, STR = Striatum, TH = Thalamus, VIS = Visual Area. [file 40478_2020_1098_MOESM3_ESM.png]

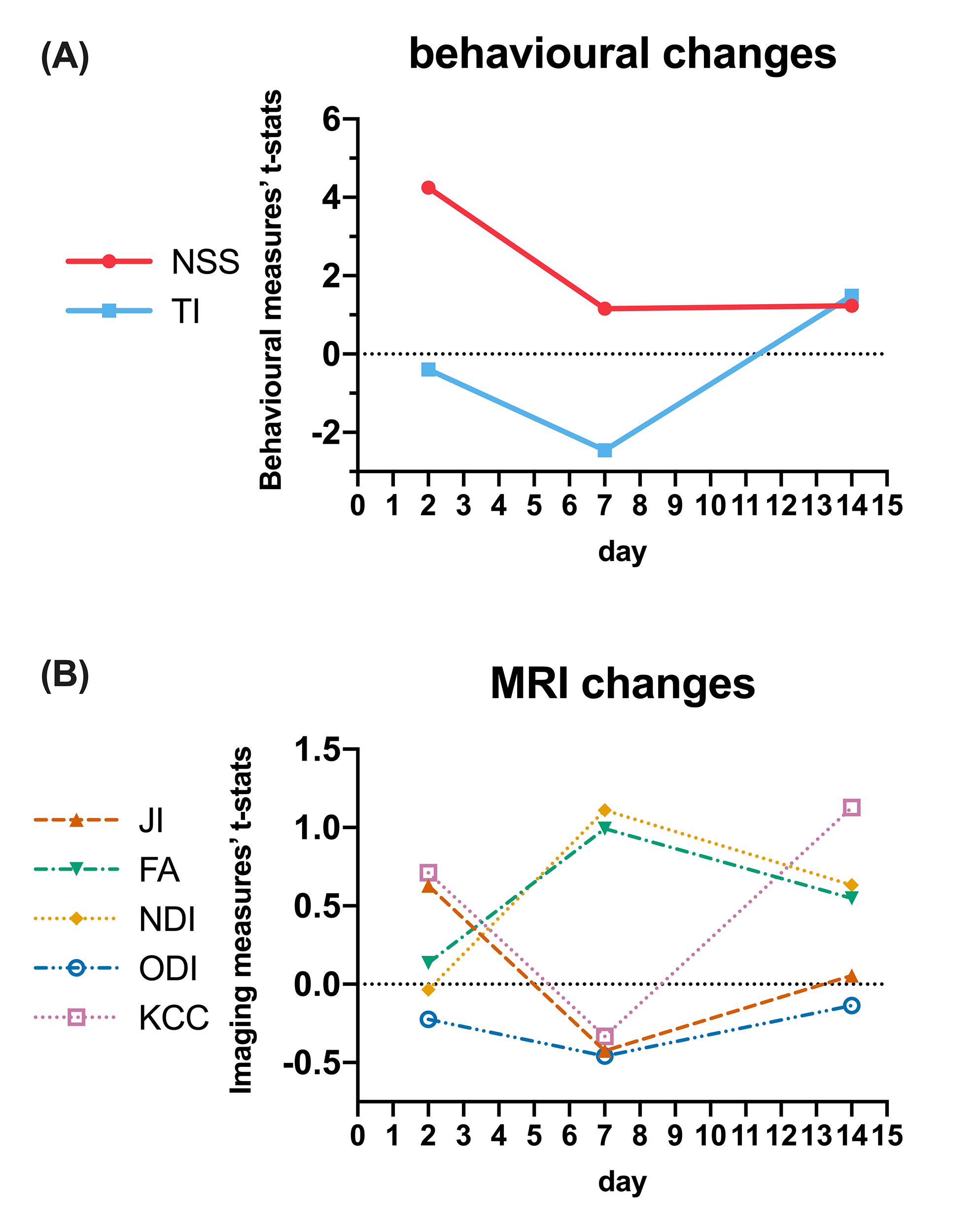

Supplement: Supplementary file 4 — Additional file 4: Figure S4. Deficit and recovery trajectories of behavioural and Magnetic Resonance Imaging (MRI) markers post-concussion. Relative changes of (A) behavioural and (B) MRI metrics of concussed cohorts relative to the sham cohorts (horizontal black dotted line through 0). Behavioural measurements (NSS and TI) scaled as t-statistics of the corresponding CON vs. sham comparison. MRI biomarkers (JI, FA, NDI, ODI, KCC, and stim-fMRI) scaled as whole-brain averaged t-statistics of the corresponding CON vs. sham comparison; whole-brain t-statistics were used as a biomarker proxy that incorporated both extents and degrees of change. NSS = Neuro Severity Score, TI = Thigmotaxis Index, JI = Jacobian Index, FA = Fractional Anisotropy, NDI = Neurite Density Index, ODI = Orientation Dispersion Index, KCC = Kendall’s Coefficient of Concordance (resting-state functional Magnetic Resonance Imaging Regional Homogeneity), and stim-fMRI (stimulus-evoked functional Magnetic Resonance Imaging). [file 40478_2020_1098_MOESM4_ESM.png]
